# COVID-19 pandemic spread against countries’ non-pharmaceutical interventions responses: a data-mining driven comparative study

**DOI:** 10.1186/s12889-021-11251-4

**Published:** 2021-09-01

**Authors:** Konstantinos F. Xylogiannopoulos, Panagiotis Karampelas, Reda Alhajj

**Affiliations:** 1 Department of Computer Science, University of Calgary, Calgary, Alberta Canada; 2 Hellenic Air Force Academy, Dekelia Air Base, Attica, Greece; 3 Department of Health Informatics, University of Southern Denmark, Odense, Denmark

**Keywords:** COVID-19, NPIs, Non-pharmaceutical interventions, Clustering, Data analysis, Seasonal infections

## Abstract

**Background:**

The first half of 2020 has been marked as the era of COVID-19 pandemic which affected the world globally in almost every aspect of the daily life from societal to economical. To prevent the spread of COVID-19, countries have implemented diverse policies regarding Non-Pharmaceutical Intervention (NPI) measures. This is because in the first stage countries had limited knowledge about the virus and its contagiousness. Also, there was no effective medication or vaccines. This paper studies the effectiveness of the implemented policies and measures against the deaths attributed to the virus between January and May 2020.

**Methods:**

Data from the European Centre for Disease Prevention and Control regarding the identified cases and deaths of COVID-19 from 48 countries have been used. Additionally, data concerning the NPI measures related policies implemented by the 48 countries and the capacity of their health care systems was collected manually from their national gazettes and official institutes. Data mining, time series analysis, pattern detection, machine learning, clustering methods and visual analytics techniques have been applied to analyze the collected data and discover possible relationships between the implemented NPIs and COVID-19 spread and mortality. Further, we recorded and analyzed the responses of the countries against COVID-19 pandemic, mainly in urban areas which are over-populated and accordingly COVID-19 has the potential to spread easier among humans.

**Results:**

The data mining and clustering analysis of the collected data showed that the implementation of the NPI measures before the first death case seems to be very effective in controlling the spread of the disease. In other words, delaying the implementation of the NPI measures to after the first death case has practically little effect on limiting the spread of the disease. The success of implementing the NPI measures further depends on the way each government monitored their application. Countries with stricter policing of the measures seems to be more effective in controlling the transmission of the disease.

**Conclusions:**

The conducted comparative data mining study provides insights regarding the correlation between the early implementation of the NPI measures and controlling COVID-19 contagiousness and mortality. We reported a number of useful observations that could be very helpful to the decision makers or epidemiologists regarding the rapid implementation and monitoring of the NPI measures in case of a future wave of COVID-19 or to deal with other unknown infectious pandemics. Regardless, after the first wave of COVID-19, most countries have decided to lift the restrictions and return to normal. This has resulted in a severe second wave in some countries, a situation which requires re-evaluating the whole process and inspiring lessons for the future.

**Supplementary Information:**

The online version contains supplementary material available at 10.1186/s12889-021-11251-4.

## Background

Historically, the humanity has faced a number of global disasters, epidemic and pandemics. These include the two world wars, the American civil war, the Spanish epidemic, influenza pandemics [1], and most recently SARS, MARS, N1H1 pandemics, among many others. However, COVID-19 may be described as the pandemic which has spread by far the most to the extent that it is almost impossible to locate a region on earth not affected by the virus. It swiftly enforced global and local economic recessions and global horror with uncertain future. More dangerous is that the virus has not yet been precisely linked to any season or region; its center and main concentration has been rapidly moving with some South American countries, India and Russia recently identified as new major hot spots. All declarations and explanations related to the pandemic seem to be nothing beyond speculations based on partial discoveries and predictions. Officials, experts and even normal people continue to debate whether the virus is natural or manufactured, whether it vanishes in the summer due to hot weather, whether there will be other waves in the winter and beyond, etc.

### Related work

It did not take the research community long to realize the need to get involved in the global effort to understand the new novel virus COVID-19. Researchers contributed from different perspectives to address various aspects related to COVID-19, including genetic, pharmacological, spreading power, health and economic effect, a model to study its lifetime, and the success of various countries in their fight against the virus. For instance, He et al. [2] conducted a comparative analysis of the transmission patterns of COVID-19 in China, South Korea, Italy and Iran. Kouba [3] developed a dashboard for comparative data analytics to study various aspects of COVID-19 spreading as compared to other viruses which affected humans in the previous decades.

Yu et al. [4] investigated how clinical decision and policy making can highly benefit from systematic reviews (SRs) which have been conducted on the COVID-19 outbreak and its predecessors. Fong et al. [5] investigated observational and simulation studies covering the effectiveness of multiple mitigation measures introduced by governments worldwide. Cirrincione et al. [6] tried to develop a vision to prevent the spread of COVID-19. For this, they considered the measures introduced by various countries. Indeed, social distancing has been addressed by various research groups as an effective measure to deal with different types of viruses, e.g., [5, 7, 8]. Other research groups addressed the containment issue of the influenza pandemics, e.g., [9, 10], which may be considered as having some common symptoms and the spreading power with COVID-19, though to a limited extent. Castorina, Iorio and Lanteri [11] applied some statistical models of curve fitting to study the effectiveness of containment on the spread of COVID-19. They traced their fitted curves by considering the daily announced numbers. Lai et al. [12] investigated the effectiveness of the reproduction of COVID-19.

Grassly and Fraser [13] described mathematical models of infection disease transmission. Herzog, Blaizot and Hens [14] conducted a systematic review of mathematical models which could guide study design or surveillance systems in infection diseases. Li et al. [15] covered the early transmission dynamics of COVID-19 in Wuhan. Jung et al. [16] handled the risk of death from COVID-19 with a real time estimation based on inference from exported cases. Shereen et al. [17] studied the characteristics of COVID-19 by highlighting its origin and spread potential. Riou and Althaus [18] discussed the early human to human transmission pattern of COVID-19.

Linto et al. [19] conducted a statistical analysis of publicly available event-date data to study the incubation period and other time intervals that govern the epidemiological dynamics of COVID-19. Li et al. [15] studied the early transmission dynamics of COVID-19 in Wuhan. The transmission of COVID-19 in Wuhan was also investigated by Nishiura et al. [20]. Hua and Shaw [21] reported that there was an initial delay in response to the need to properly dealing with COVID-19. For this, they studied the effect of the various measures introduced by the Chinese officials to combat the COVID-19 virus. They based their study on local newspapers, social media and data captured from other digital platforms. Lin et al. [22] developed a model which was inspired from the 1918 influenza pandemic in London, United Kingdom by considering the common factors applicable to COVID-19, including NPI’s. They applied their model to study the virus outbreak in Wuhan. The same authors reported other research effort where their work is data driven which also tried to estimate the unreported number of COVID-19 in China at early stage [23, 24].

Arianna and Giudici [25] utilized a Poisson autoregressive model to understand COVID-19 Contagion Dynamics. Barro, Ursúa, and Weng [26] investigated how it is possible to benefit from the measures taken against the Spanish Flu in fighting COVID-19. Markel et al. [27] described the NPIs applied by various US cities during the 1918–1919 influenza pandemic. Yang and Zeng [28] utilized a modified SEIR model and estimated the trend of COVID-19 in China. Tuite and Fisman [29] reported on various aspects of COVID-19, including its growth and reproduction numbers. Anderson et al. [30] investigated the effect of the introduced measures on the spread or containment of the virus. Saunders-Hastings, Reisman and Krewski [31] studied the benefit of early intervention on the containment of pandemic influenza transmission.

To sum up, the outbreak of COVID-19 has attracted world-wide attention from all sectors of the community from officials to the general public. Researchers have been heavily involved in efforts to address various aspects associated with the virus from genetics to statistics. However, we are not aware of any comprehensive study which has covered the analysis of COVID-19 associated data in the details and the scope to the level addressed in this paper. Indeed, one of the main targets of this study is to have a single unique and comprehensive reference which will enlighten, and guide all stakeholders involved in understanding the effectiveness of the announced diverse NPI measures and steps implemented by different countries to deal with COVID-19.

### Problem definition

The COVID-19 pandemic took by surprise both scientists and politicians since SARS-Cov-2 was a new virus with unprecedent contamination rate not matching other viruses of the same family. As a result, epidemiologists and decision-makers were not initially in a position to identify which measures should be taken and when in order to protect the general population of their countries and, consequently, control the spread of the virus. Several countries followed different containment strategies. Indeed, there is a lot of public discussion regarding the most appropriate strategy to limit the number of deaths caused by the virus while minimizing the economic cost.

World Health Organization (WHO) announced SARS-Cov-2 as a pandemic on March 11, 2020. Motivated by the different Non-Pharmaceutical Intervention (NPI) policies, adopted by various countries and the respective time of their application, in this paper we attempt to compare them based on several parameters, such as confirmed cases, deaths, population density, etc. to discover possible associations. To achieve this, a number of issues related to the data have to be addressed beforehand. First, there is a plethora of data sources, both governmental and institutional, providing daily updates on the pandemic facts (cases and deaths) related to the virus worldwide. Because of the different adopted reporting strategies --something that makes the validation of the data challenging-- we had to select an official and reliable main source such as the European Centre for Disease Prevention and Control [32]. Additionally, we have used national institutional and governmental sources to cross-validate, clean, and prepare the dataset in the most reliable way possible. Second, the collection of NPIs policies had to be manually collected, checked, and translated, because to the best of our knowledge there is no single source that could provide such information reliably. The specific dataset was prepared by acquiring the data from the official governmental gazettes and national health system announcements regarding the applied NPIs.

Our main hypothesis is that NPIs can significantly control the spread of an infectious disease. Our objective is to compare the effectiveness of NPI measures in relation to the contagiousness and mortality of COVID-19 since it is not clear which measures may or may not be affective. Using data mining and machine learning approaches, for the time series corresponding to the confirmed cases and deaths and the corresponding NPIs, we expect to discover possible relations between disease spread and mortality trends and NPIs. Furthermore, we attempt to use a novel research approach which depends on geographical and social attributes for each country. More precisely, we used urban population density to explore whether the confirmed cases and deaths are also related to the NPI measures taken to control the spread of the disease.

To sum up, we attempt to validate our hypothesis by addressing the following research questions with regards to the effectiveness of the NPI measures against the spread of COVID-19:

- Which countries share similar trend of confirmed cases and deaths?
- Is there any correlation between the NPI measures and the evolution of the confirmed cases and deaths in the investigated countries?
- If yes, what were the NPI measures which influenced mostly the evolution of the confirmed cases and deaths in countries that have the same trend?
- Is there any indication that a specific NPI measure is more effective than the other measures?
- What positive and negative lessons could be learned from the experience of the various countries regarding the implementation of the NPIs and how these lessons can help in better shaping the action plans for more effective prevention or containment of future outbreaks?

By answering all these questions, our study will contribute to a better understanding of the implications of NPIs in the contagiousness and mortality of the virus. Furthermore, we will systematically collect data related to NPIs and provide significant and detailed source references that the scientific community could easily access for further study. The novelty of this approach can be articulated as follows: by applying data mining and clustering analysis methods, which can be agnostic to the underlying raw data and unbiased in contrast to early epidemiological studies, we can observe the impact of enforcing the NPI measurements at the very early stages of the pandemic.

## Methods

Our research study follows the standard phases of the data mining and analytics process. The first phase consists of defining and categorizing the data to be used in the analysis. The second phase comprises data gathering and acquisition from reliable sources. The collected datasets require to be cleaned and prepared for the next phase which is data analytics. In this phase, we apply different techniques from data mining, time series analysis, pattern detection, machine learning, data clustering, etc. Then, we visualize the findings in the most appropriate way to illustrate any possible information of interest for acquiring useful knowledge. Lastly, based on the results, we discuss the questions stated in the previous section and figure out how they could be addressed effectively. More specifically, our work starts by exploring the potential NPI measures proposed by various Health Organizations, including the World Health Organization (WHO), the European Centre for Disease Prevention and Control (ECDC), the US Center for Disease Control and Prevention (CDC), etc. We have selected and categorized the considered NPI measures under specific categories which can be easily monitored and analyzed later in the study.

Due to the lack of a single source of NPIs records, data related to the specific policies had to be manually collected from the official governmental gazettes and the national health system announcements regarding the NPIs applied in each country. Additionally, different sources of data reporting SARS-CoV-2 confirmed infected and death cases were examined, and then it was decided to use only official and reliable sources, such as ECDC and governmental institutions, to collect the pre-defined data. Consequently, we performed pre-processing to clean, normalize, transform, and prepare the data for the analysis phase. In this phase, advanced data mining techniques have been used to analyze the time series of the confirmed infected and death cases. The process involves using multivariate data structures (LERP-RSA) that allow the ARPaD algorithm [33–35] to detect common patterns in the aforementioned series, feeding them to the GPSC algorithm [36] to detect commonalities between the series, which are clustered using the DBSCAN algorithm [37]. To provide a more comprehensive view of the findings, the final results have been visualized using a variety of alternatives, including tables, plots, diagrams and novel bubble plots.

### Non-pharmaceutical intervention measures

As the transmission of the COVID-19 virus culminated among travelers from China and progressively to other countries, WHO created several guidelines to support governments to plan and manage the pandemic [38]. Other organizations, such as ECDC and CDC created a number of guidelines for different settings, including educational institutions, workplaces, communities, etc. based on the experience drawn from similar pandemics, e.g., influenza and ebola [32, 39]. The guidelines thoroughly present the different scenarios based on the state of transmission of the virus and the suggested measures for the health systems, high-vulnerability settings, workplaces, and the general community.

In this paper, our focus is on the public health and social measures (PHSM) suggested by WHO [40, 41], the public health measures mentioned by ECDC [42] or NPIs specified by CDC [5], and how these measures have impacted the transmission and the mortality of COVID-19 in a number of countries.

Based on the toolbox prepared by ECDC, the main tools that governments can use, fell under the following categories:

- Travel measures that aim to limit the transmission of the virus from external sources.
- Personal protective measures aiming at restricting the possibility of virus contamination for persons who operate in a high-risk environment.
- Social distancing measures to eliminate the mobility of contaminated people and the transmission of the virus to the general population.
- Antiviral medicine when available.
- Vaccines when available.

Since at the time of the specific study, there was no known effective antiviral or vaccine, the study has been limited to the three other public health measures, namely, travel, personal and social distancing measures that were implemented by various governments in order to face the COVID-19 pandemic. As per WHO statement, such measures are not yet assessed in terms of the social and economic impact. Thus, WHO proposes for the governments to carefully “balance the benefits and potential harms” of the specific measures to mitigate the transmission of COVID-19 [40]. The proposed measures with a cost-effectiveness analysis are listed in Table 1.

**Table 1:** Measures with their associated cost and effectiveness (adopted from [42])

Category	Measure	Effectiveness	Direct cost	Secondary effect
Travel measures	Travel advice	Minimal	Small	Large
	Entry screening	Minimal	Large	Moderate
	Border closure	Minimal unless rapid	Massive	Massive
Personal protective measures	Regular hand-washing	Self-evident	Moderate	None
	Respiratory hygiene	Self-evident	Small	Small
	General mask-wearing	No evidence	Massive	Small
	Mask-wearing in healthcare settings	Unknown	Moderate	Small
	Mask-wearing in other high-risk situations	Unknown	Moderate	Small
	Mask-wearing by people with respiratory symptoms	Unknown	Moderate	Perverse effects
	Voluntary isolation of cases not requiring hospitalization	Self-evident	Moderate	Moderate
	Voluntary quarantine of household contacts	Self-evident	Massive	Massive
Social distancing measures	Internal travel restrictions	Minor delaying effect	Major	Massive
Educational measures	Reactive school and day care closures	Positive effects	Moderate	Massive
	Proactive school and day care closures	Positive effects	Moderate	Massive
Workplace and public place measures	Reactive workplace closures	No evidence	Massive	Major
	Home working	No evidence	Variable to moderate	Variable to moderate
	Cancelling public gathering, events	Positive effects	Major	Major

#### Travel measures

For highly contagious viruses, traveling acts as the main gate to rapidly spreading worldwide, and thus governments should closely monitor the situation in other countries or areas, especially in the early stages of a potential epidemy. Travel monitoring does not necessarily mean immediately imposing strict measures, such as border closure or passengers’ scrutinizing. Instead, the process may start issuing travel advice or warnings against traveling, by targeting a specific destination, a specific continent, or worldwide.

Entry screening is a less disturbing and intruding measure for a traveler. It is considered appropriate for early stages of an epidemy. This can be applied to the major entries of a country, including airports, seaports, and land border stations, to screen incoming travelers for symptoms related to the potential contagious disease. Traveling measures can be elevated to travel bans, forbidding the local population to travel abroad, and border closure that will prevent any incoming contaminated traffic of persons into the country. Travel measures can also be applied at a territory level. This means some high-risk areas can be isolated from the rest of the world, or some healthy areas can be shielded against travelers coming from territories with high contamination rates of the virus. Other forms of implementing travel measures are possible based on need and coverage. The ultimate goal is to isolate a given region as part of the effort to prevent the spread of a transmittal disease.

#### Personal protective measures

Personal protective measures can be distinguished as measures related to various aspects of the daily life. One important measure under this category is personal hygiene, such as regular hand-washing and proper disposal of potentially infected materials, e.g., tissues, cutlery, etc. Mask-wearing in various settings is essential, depending on the risk and personal isolation, when there is a potential to be exposed to a crowd which brings up a risk that some persons have been exposed to the virus. Similarly, as in travel measures, personal protective measures can be escalated, e.g., for the general population or for a specific group of high-risk individuals.

#### Social distancing measures

Social distancing measures usually refer to the increase in physical distancing between people to avoid transmission from one individual to another. There is a large number of measures that fall under this specific category. However, their effectiveness is debatable and must be examined individually [42]. Social distancing has been studied in closed environments, such as schools and workplaces. Based on the findings, it seems that social distancing reduces the possibility of virus transmission [8]. Social distancing is usually combined with other measures, such as restrictions in public gatherings or organized events.

#### Educational measures

Another important set of measures in the attempt to contain a highly contagious disease is related to schools (educational institutions) closure. This is important because a school is a spot of large gatherings. Social distancing is hard to respect inside and outside a classroom. Indeed, students are not in a position to always pay attention to personal hygiene measures or social distancing. In this sense, closing schools will prevent the virus from contaminating to other families and associated communities, and thus will limit its activity. This set of measures can also be expanded to other forms of educational institutions, including universities or day-care schools. In this case, university closure can also be considered as an important mean of imposing the social distancing measure [5], since all academic stakeholders remain isolated during the closure.

#### Workplace and public place measures

This category of measures includes not only the closure of workplaces, but it is also combined with home working, teleconferencing, closure of shops and malls, i.e., general places where a lot of people may gather. Transportation systems, places of worship, cultural places and sport arenas are covered by this specific category of measures. These measures are usually combined with personal hygiene measures, such as frequent cleaning and sanitizing of work benches, special configuration of air-filtering systems, etc. [6].

### Categorization of the selected NPIs

From the beginning of January 2020, when the COVID-19 virus started to spread in China, several countries closely monitored its evolution and impact. Several governments resorted to their national crisis management units and their epidemiologists to devise their strategy against the new virus. Based on the available tools suggested by WHO to handle a highly contagious disease, different strategies were formulated globally. In this paper, an attempt has been made to collect and compare the different measures taken by a number of countries as part of their effort to fight COVID-19. The measures are then projected to the number of confirmed cases in an attempt to analyze the effectiveness of the measures based on hard facts.

As most of the countries tried to slow down the spread of respiratory illnesses such as COVID-19 by introducing measures that were available in the WHO toolbox, a more granular data collection was conducted using the five categories of Public Health and Social Measures proposed by WHO and ECDC [40–42].

More specifically, four different measures were recorded for the category of travel measures:

- *Travel advice for China*. This measure is very important since China was the origin of the new virus. It is interesting to figure out how early each country took the specific measure, and how this measure contributed to the evolution of infected and death cases in countries that took this measure.
- *Travel advice to avoid traveling abroad*. This measure is equally important since limiting traveling to insecure areas with a lot of confirmed cases of COVID-19 may hinder the contamination of the local population.
- *Border closure for passengers from and to all destinations but excluding goods or medical supplies transportation*. This measure is stricter with several consequences. It is interesting to investigate whether this measure was adopted by various countries and when.
- *Suspension of visa services*. In most cases, this coincided with border closure. Thus, this was not finally included in the study and was left out as superfluous information.

As mentioned above, the second category of personal hygiene measures includes measures that cannot be closely monitored, such as wearing a mask, hand-washing and proper disposal of tissues, etc. These are generic recommendations that were made before the beginning of the first cases in all countries. Thus, these measures were not included in the study. In this category, the following measure adopted by several countries was monitored:

- *Personal isolation of a potential virus carrier*. This is a measure that was announced by several countries and was monitored as a 14-day quarantine of all incoming travelers.

The third category of measures, namely social distancing is too generic, especially when it pertains to physical distancing, and thus the specific measure cannot be recorded or imposed by the governments. However, other measures that aimed at applying social distancing were recorded. These include:

- *Bars and restaurants closure*. The specific measure hinders people from socializing in a restaurant or bar, and thus tries to impose keeping distance.
- *Regulations on citizens’ movements*. In this case, citizens had to stay at home isolated, or in some cases they were allowed to leave their houses for extraordinary circumstances, e.g., to visit grocery stores for shopping, to visit doctors for emergency medical cases, necessary personal training, etc.
- *Complete lockdown of a country*. A strict measure that aims at limiting the social interaction of citizens, and thus protects the general population from contaminating by the virus, especially when the virus is already active in a country. It is very interesting to explore when it is the appropriate time to take such a measure, and what is its effect on the evolution of the virus spreading.

The fourth category of toolset contains measures oriented towards educational institutions, and thus the following was recorded:

- *Schools and Universities closure*. As most of the countries took this measure, it is interesting to explore at what stage of the pandemic governments decide to close schools and universities.

The fifth category of measures is related to restrictions to the workplaces and public places. Among other measures, homeworking is proposed from WHO. However, it was not easy to study this measure because there are several different jobs in which homeworking is not possible. Thus, the following measures were recorded related to public and private workplaces:

- *Closure of entertainment and cultural places*. This includes the closure of theaters, cultural centers, cinemas, museums, etc.
- *Sporting facilities closure*. This refers to the closure of gyms, parks, swimming pools, ski resorts, wellness centers, etc.
- *Sport events suspension*. Events such as football games, basketball games, tennis, etc. in several countries were canceled or suspended.
- *Religious services suspension*. This aims at restricting people from participating in religious events, including masses, funerals, and other ceremonies.
- *Cancelation of events with more than 5000, 1000, 500, 100 and 10 persons*. Several countries applied social distancing measures in public places by forbidding gatherings of sizes deemed risky.

### Data collection and preprocessing

Appropriate data are required to effectively tackle the aforementioned research questions. A variety of sources were investigated to collect the most precise data that will lead to more accurate results. Despite the fact that most countries usually follow a specific protocol to report confirmed infected and death cases, yet, the available data suffers from severe discrepancies, regardless the source used by the specific organization (WHO [43], CDC [44], ECDC [45], etc.). Independent institutions, such as Johns Hopkins University [46], worldometers.info [47], ourworldindata.org [48] have also collected data from various sources. However, even in these cases, same date data differ not only among these sources, but also compared to the official data reported to WHO. In many cases, several countries across Europe and Worldwide misreported or failed to follow the standard reporting protocol leading to inconsistencies. For example, UK reported deaths only from hospitalized cases; deaths from retirement homes were not reported at the beginning [49]. In other situations, such as Italy or Spain, the healthcare system was overwhelmed, and confirmed infected and death cases were reported days after they occurred. In other occasions, data monitoring organizations revised the pre-recorded values and changed them to match the actual numbers reported later by the officials [50]. As a result, the official data reported by the world organizations showed in some occasions zero or even negative numbers as infected or death cases in order to align the aggregated totals. This happened with the data published by ECDC about the geographic distribution of COVID-19 cases worldwide where the confirmed cases of Spain on 19/4/2020 were initially reported as − 713 cases, and the confirmed cases of Lithuania on 29/4/2020 were initially reported as − 105 cases [45].

Unfortunately, these inconsistencies in the data recording lead to highly noisy data. To overcome this problem, the time series collected for various countries were filtered using the Butterworth filter [51]. The filtering produced smooth time series that better represent the underlying actual data by applying any possible functionality, such as low-pass, high-pass, etc. This is depicted in the appendices where the actual data is presented with red dotted lines, while the filtered data is presented with blue continuous lines, and the cumulative time series are presented with gray bars. In the green description at the upper left side of each subplot, we can see the country code and the date of the first incident (infected or death). At the upper right side, we can see the cumulative incidents (infected or death) projected as a ratio per 1 million inhabitants or 1000 km^2^ of urban land area, and the cardinality of the time series appears under that. Additionally, there are other factors that increased noise in the dataset, like the detection of several cases on a ship and several asymptomatic carriers in a refugee camp in Greece [52, 53], the use of more reliable tests, etc.

Another important step in data preparation is to determine the size of the two time series categories (confirmed and death cases). Because confirmed cases are longer than death cases time series, for our analysis we have used two phases of data collection. The first one has a cut-off date of May 18th and the second has a cut-off date of May 29th. The reason for this double phase data collection was to have: (1) enough data to perform the time series and NPIs analysis, and (2) additional data that would allow us to properly align the confirmed and deaths cases time series for visualization purposes based on NPIs Measures Comparison Section for countries which have their time series shorter than the standard cut-off date of May 18th. For the confirmed cases time series, we have used a 70-days interval, while for the death cases time series we have used a 56-days interval based on the first cut-off date. For countries which have their time series shorter than 70 data points at the first cut-off date, the second cut-off date has been used to expand the time series and make their diagrams comparable in terms of the number of data points.

Moreover, because of the aforementioned data inconsistencies, we had to find a way to determine the date of the first case in each country to be able to align the time series as much as possible for clustering purposes. For this reason, we have chosen as the first confirmed case date, the first day of seven continuous days in which there was at most 1 day out of the seven without a confirmed case. On the other hand, other researchers used as first day of counting, the day that the 100th confirmed case occurred. This was necessary especially for countries where there were sporadic cases at the beginning of the pandemic, e.g., Belgium, mainly because of visitors or tourists who travelled back from China. They were isolated very early, but they weren’t considered as they didn’t contribute to the general population infection, which started to happen several days later [54]. However, such an assumption may not be precise because it was found later that the actual symptoms of COVID-19 may appear within 14 days from the infection. For those countries, all incidents reported before the first date, as defined above, were summed up and appear before the first confirmed case day, leading to a confirmed cases time series with an overall length of 71 days. Another reason that led us to select the specific time interval could be justified by the need to select as many countries as possible without having very short time series since some countries started reporting the cases very late, e.g., Russia and Brazil.

**Fig. 1:**
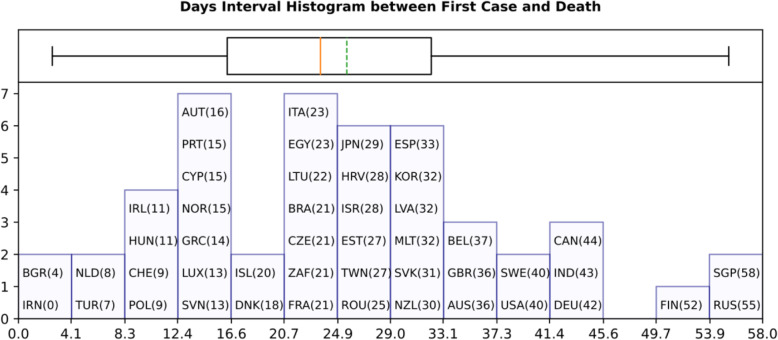
Days Interval Histogram between First Case and Death

As shown in Fig. 1 which presents the time interval between the first (actual) confirmed infected and death cases, there should be several cases of probable misreporting. For example, Iran reported the first confirmed infected and death cases in the same day, having a zero-time interval between confirmed cases and deaths. On the other hand, Singapore has a time interval of 58 days because of incoming tourists’ who were timely screened and isolated. Additionally, Fig. 1 reports the existence of two main groups of countries with time intervals of around 13 days between the first confirmed case and the first death case. This coincides with the estimated time between symptoms to death according to WHO [16]. Another group of countries reported the first death case around 26 days after the first confirmed infected case. Finally, all values for the confirmed infected and death cases were projected either per one million of population or 1000 km^2^ of urban land area to be comparable among different countries.

A range of different sources were examined to collect the measures and their corresponding application dates. The sources were identified manually, and the data were collected by only one of the researchers to apply a common rationale for all the countries regarding the type of applied measures and the corresponding dates. The travel measures were mainly collected from the official pages of the Ministry of Foreign Affairs in each country, while the rest of the measures were collected either using the official gazette of each government which was automatically translated to English, or by the official sites of the authorities designated to handle the health crisis in each country. This was possible because most countries have created a website dedicated to COVID-19; the website includes information for the public regarding the infected cases, the death cases, and the measures. If none of the above-mentioned official websites noted the timeline of the measures or lacked information regarding a specific taken measure, a broader search was carried out and the proper information was extracted either from news reports or from other websites that cumulatively reported such measures, e.g., by UNESCO.

Here, it is important to note a discrepancy which may occur in the data. This is related to the difference between the date when the measures were announced by some countries and the actual date when the measures were implemented. In some cases, it was not clear when the measures were exactly applied since for example, a spokesperson of a government may have announced the measures on a specific date, but the actual implementation might have started some days after the official announcement. When the dates were not mentioned in the official decrees of the governments, the dates reported by the news sites were used. Table 2 summarizes the sources used for each of the countries considered in this study.

**Table 2:** Sources of measures data per country

Country	Sources	Country	Sources
Australia	AUS1, AUS2, AUS3, AUS4	Japan	JPN1, JPN2, JPN3, JPN4
Austria	AUT1, AUT2, AUT3	Latvia	LVA1, LVA2, LVA3, LVA4, LVA5, LVA6
Belgium	BEL1, BEL2	Lithuania	LTU1, LTU2, LTU3, LTU4, LTU5, LTU6, LTU7
Brazil	BRA1, BRA2	Luxembourg	LUX1, LUX2, LUX3, LUX4, LUX5, LUX6, LUX7, LUX8, LUX9, LUX10, LUX11, LUX12, LUX13, LUX14
Bulgaria	BGR1, BGR2, BGR3, BGR4, BGR5, BGR6	Malta	MLT1, MLT2, MLT3, MLT4, MLT5, MLT6
Canada	CAN1, CAN2, CAN3, CAN4	Netherlands	NLD1, NLD2, NLD3, NLD4, NLD5, NLD6
China	Not included	New Zealand	NZL1, NZL2, NZL3, NZL4
Croatia	HRV1, HRV2, HRV3, HRV4	Norway	NOR1, NOR2
Cyprus	CYP1, CYP2, CYP3, CYP4	Poland	POL1, POL2, POL3
Czechia	CZE1, CZE2	Portugal	PRT1, PRT2, PRT3, PRT4, PRT5, PRT6
Denmark	DNK1, DNK2	Romania	ROM1, ROM2, ROM3, ROM4
Egypt	EGY1, EGY2, EGY3	Russia	RUS1, RUS2, RUS3
Estonia	EST1, EST2	Singapore	SGP1, SGP2, SGP3
Finland	FIN1, FIN2	Slovakia	SVK1, SVK2, SVK3
France	FRA1, FRA2, FRA3, FRA4, FRA5, FRA6	Slovenia	SVL1, SVL2
Germany	DEU1, DEU2, DEU3	South Africa	ZAF1, ZAF2, ZAF3, ZAF4, ZAF5
Greece	GRC1, GRC2, GRC3, GRC4, GRC5, GRC6, GRC7	South Korea	KOR1, KOR2, KOR3, KOR4
Hungary	HUN1, HUN2, HUN3	Spain	ESP1, ESP2, ESP3
Iceland	ISL1, ISL2, ISL3	Sweden	SWE1, SWE2
India	IND1, IND2, IND3	Switzerland	CHE1, CHE2, CHE3, CHE4, CHE5
Iran	IRN1, IRN2	Taiwan	TWN1, TWN2, TWN3, TWN4
Ireland	IRL1, IRL2, IRL3, IRL4, IRL5, IRL6	Turkey	TUR1, TUR2, TUR3
Israel	ISR1, ISR2	United Kingdom	GBR1, GBR2, GBR3
Italy	ITA1, ITA2, ITA3	United States of America	USA1, USA2, USA3, USA4
Global sources	GLR1, GLR2, GLR3, GLR4, GLR5, GLR6, GLR7, GLR8		

The aforementioned measures have been collected for the period between middle of March 2020 and end of May 2020, going retrospectively from January 1st to the 18th of May 2020. The rationale behind the selection of the countries was initially to record how different countries in the European Union had reacted to the pandemic and whether the European Commission managed to coordinate the different governments to react to the pandemic in a similar and effective way. However, later the study was expanded to other countries that had either adopted a different strategy to handle the pandemic or where the confirmed cases and deaths were going out of control. Progressively, other countries with special characteristics, i.e., isolated, or islandic countries such as New Zealand, Australia, Iceland, etc. were covered in the study. As a result, a selection of 48 countries was studied (Table 3).

**Table 3:** Countries in the dataset

Country	Code	Continent	Country	Code	Continent
Australia	AUS	OC	Japan	JPN	AS
Austria	AUT	EU	Latvia	LVA	EU
Belgium	BEL	EU	Lithuania	LTU	EU
Brazil	BRA	AM	Luxembourg	LUX	EU
Bulgaria	BGR	EU	Malta	MLT	EU
Canada	CAN	AM	Netherlands	NLD	EU
China	CHN	AS	New Zealand	NZL	OC
Croatia	HRV	EU	Norway	NOR	EU
Cyprus	CYP	EU	Poland	POL	EU
Czechia	CZE	EU	Portugal	PRT	EU
Denmark	DNK	EU	Romania	ROU	EU
Egypt	EGY	AF	Russia	RUS	AS
Estonia	EST	EU	Singapore	SGP	AS
Finland	FIN	EU	Slovakia	SVK	EU
France	FRA	EU	Slovenia	SVN	EU
Germany	DEU	EU	South Africa	ZAF	AF
Greece	GRC	EU	South Korea	KOR	AS
Hungary	HUN	EU	Spain	ESP	EU
Iceland	ISL	EU	Sweden	SWE	EU
India	IND	AS	Switzerland	CHE	EU
Iran	IRN	AS	Taiwan	TWN	AS
Ireland	IRL	EU	Turkey	TUR	AS
Israel	ISR	AS	United Kingdom	GBR	EU
Italy	ITA	EU	United States of America	USA	AM

Two major groupings have been created for the analysis of the confirmed infected and death cases. The first grouping covers the ratio of confirmed and death cases per 1 million population. When we need to compare countries, this is much more reliable than using the absolute numbers since it is crucial to check the impact that the infection has on the general population using a standardized metric. For example, USA has approximately 10 times more deaths than Belgium, 89,562 and 9052 (May 18th 2020), respectively, yet, USA has approximately 33 times larger population, 327.2 million in USA compared to 11.4 million in Belgium. Therefore, the ratio of deaths per one million of population is practically 3 times more for Belgium compared to USA, 792.7 and 273.75 respectively. This shows that for the period considered in this study the pandemic has a much heavier impact in Belgium compared to USA.

The second grouping is based on population density per 1000 km^2^. This is important to be able to compare countries that have approximately the same population but with considerably different distribution. For example, Germany has significantly larger number of cases compared to France, 174,697 and 142,411 respectively. However, France has slightly more cases per 1 million compared to Germany, 2125.94 and 2106.61, respectively. However, if we compare countries based on their density, Germany has significantly higher cases ratio per 1000 km^2^ compared to France, 2800.79 and 1647.07, respectively.

Here, it is essential to mention that we have used another density measure instead of the standard population density as measured by many organizations such as World Bank, OECD etc. More precisely, population density per urban land area has been used because urban areas are form the focus for implementing the NPIs measures where population density is higher compared to rural areas where the measures may not be implemented at all due to the sparse distribution of the population or may have no effect on reducing infection rates. However, such diversification significantly changes the country rankings per population density. For example, very large countries in area, but with small population, such as Australia and Canada have standard density of 3.25 and 3.71 persons per square kilometer, respectively. However, if we take into consideration only urban population and urban land area, their density is 585.01 and 238.48, respectively; the same is true for many other countries like Russia and Brazil. This happens because according to World Bank data [55], Australia and Canada have a ratio of urban population of 85.2 and 80.9%, respectively, while their urban land areas are 36,745.70 and 126,511.16 km^2^, respectively in comparison to 7,692,024 and 9984,670 total surface area, respectively. Therefore, to calculate the density of the urban land area, first the urban population is calculated based on the total population and the urban population ratio, then the urban population density is calculated based on the urban land area. For countries where the urban land area is missing [55] (Austria, Czech Republic, Hungary, Slovakia, and Taiwan), we have made an approximate calculation based on data available in [56]. More specifically, the agricultural and forest land has been subtracted from the total area, and the ratio between urban and rural land has been calculated based on the urban population ratio.

### Data mining, time series analysis and visualization

After cleaning and curating the gathered data, we conducted data analytics to cluster the time series. Two types of clustering have been performed. The first is based on the time series of the infected cases (1 + 70 days) and the death cases (56 days). This type of clustering is important to identify the trends, how the infected and death cases evolve per country, and how countries may end up clustered together.

Clustering has been achieved using the General Purpose Sequence Clustering (GPSC) Algorithm [36], which is based on the Longest Expected Repeated Pattern Reduced Suffix Array (LERP-RSA) data structure [33, 34] and the All Repeated Patterns Detection (ARPaD) Algorithm [34, 35]. One advantage of the GPSC algorithm is that it allows the clustering of a very large number of time series, regardless of their length. Furthermore, because of the unique attributes of LERP-RSA and ARPaD, GPSC can cluster sequences while eliminating, as much as possible, data points which could be identified as noise or outliers. The second type of clustering is for confirmed cases per 1 million population and per 1000 km^2^ urban land area. This is important to directly compare the impact of the infection on countries, and to identify whether and how the implemented NPI measures affected the number of cases. This type of clustering is significantly easier than the first one and can be performed using any standard clustering algorithm. For our purpose, DBSCAN [37] has been used. This clustering and the correlation with the implemented NPI measures will be discussed in detail in the next section.

The GPSC algorithm is a shape-based similarity clustering algorithm. To work properly, the first step requires standardizing the time series by Z-Scoring. This transformation allows to reform each time series to a new time series with mean zero and standard deviation one, while maintaining the shape of the underlying time series.

The next step is to discretize a time series of real numbers using a predefined alphabet which divides the time series boundaries into classes. There are many approaches for determining the alphabet by considering the distribution (e.g., same width or same frequency) and size (e.g., Sturges’, Scotts’ or Freedman/Diaconis’ rules) [57, 58]. In our case, we have chosen the Sturges’ rule, which gives seven classes of the same width, and accordingly an alphabet of size seven. Based on the considered alphabet, all the time series were discretized, and a corresponding string representation was created for each one.

The discretization process is important to create the LERP-RSA data structure. More precisely, Multivariate LERP-RSA has been used. It is a variation of LERP-RSA that allows to create a single data structure for all strings representing the discretized time series. Then, the ARPaD algorithm is executed over the LERP-RSA data structure. It has the unique ability not only to detect all repeated patterns that exist in a sequence, but as in our case, it also detects all repeated patterns that exist among different sequences in a set of series. These patterns were filtered based on the position where they occurred and, therefore, presented patterns that exist at the same position in different sequences. In our case these represent the same time intervals between the time series. Although, one could claim that it is similar to any distance-based clustering algorithm, there are several important differences. First, GPSC allows to cluster all the time series at once by calculating the distance without performing a one-to-one comparison between the time series. A one-to-one comparison is practically impossible for large numbers of time series due to the associated computational complexity. Further, another attribute of GPSC, based on ARPaD, is that it can match long patterns amid the whole set of time series. For example, if we use single character sized patterns as the comparison measure, the process becomes similar to the distance-based algorithms, i.e., comparing the distance between two data points of two time series. However, GPSC can perform the same with longer patterns of more than one character and multiple time series. This is very important because it can eliminate noise by matching time series based on long and continuous regions and, therefore, it excludes any single data point similarities which could occur because of the noisy data.

Finally, some rich and diverse visualization methods have been used to provide a more comprehensive view of the findings. More specifically, the following forms have been used: (a) several types of tables to depict discrete information, (b) boxplots to present descriptive statistics, (c) maps to illustrate the clustering of countries, (d) line plots to present trends and polynomial fittings, (e) sequence commonality matrices representing countries commonalities on confirmed cases and deaths (f) heatmaps illustrating measures taken per week and country, (g) two dimensional distance plot of countries’ confirmed infected and death cases, (h) bubble plots per major measure grouped per country over time in relation to death cases, and (i) combined bar-line plots for all countries per confirmed infected and death cases.

## Results

### Time series curve clustering with GPSC algorithm

**Fig. 2:**
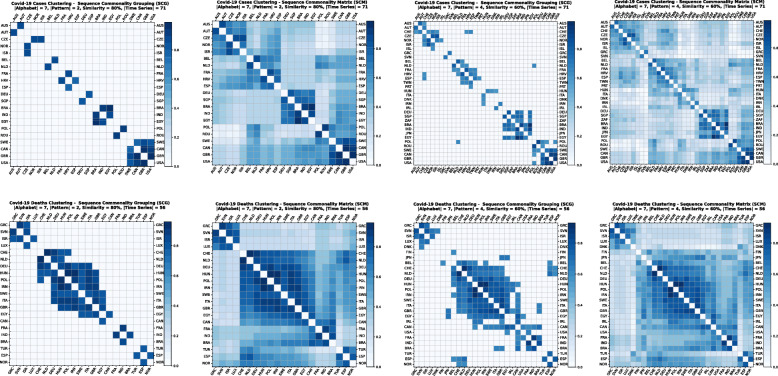
COVID-19 Cases Clustering – Sequence Commonality Grouping (a) and (c)/Matrix (b) and (d) for 80 and 60% similarity

Using the GPSC algorithm, it is possible to set different similarity percentages (scores) that reveal the closeness similarity of the time series. The algorithm returns two matrices. The first is a Sequence Commonality Grouping (SCG) Matrix [Fig. 2(a), (c)] which shows only commonalities between sequences that are equal to or above the percentage threshold. The other Sequence Commonality Matrix (SCM) [Fig. 2 (b), (d)] presents the full commonalities among all selected sequences that passed the threshold and appeared in SCG. By starting with a high similarity percentage and lowering it step by step, we can observe how various clusters are formed. For data analytics and clustering purposes, we have used a similarity percentage between 85 and 55% for confirmed cases, and between 90 and 70% for death cases. To reduce the noise for low percentage rates, a longer pattern threshold has been used, varying from 2 up to 4 letters (Fig. 2).

**Fig. 3:**
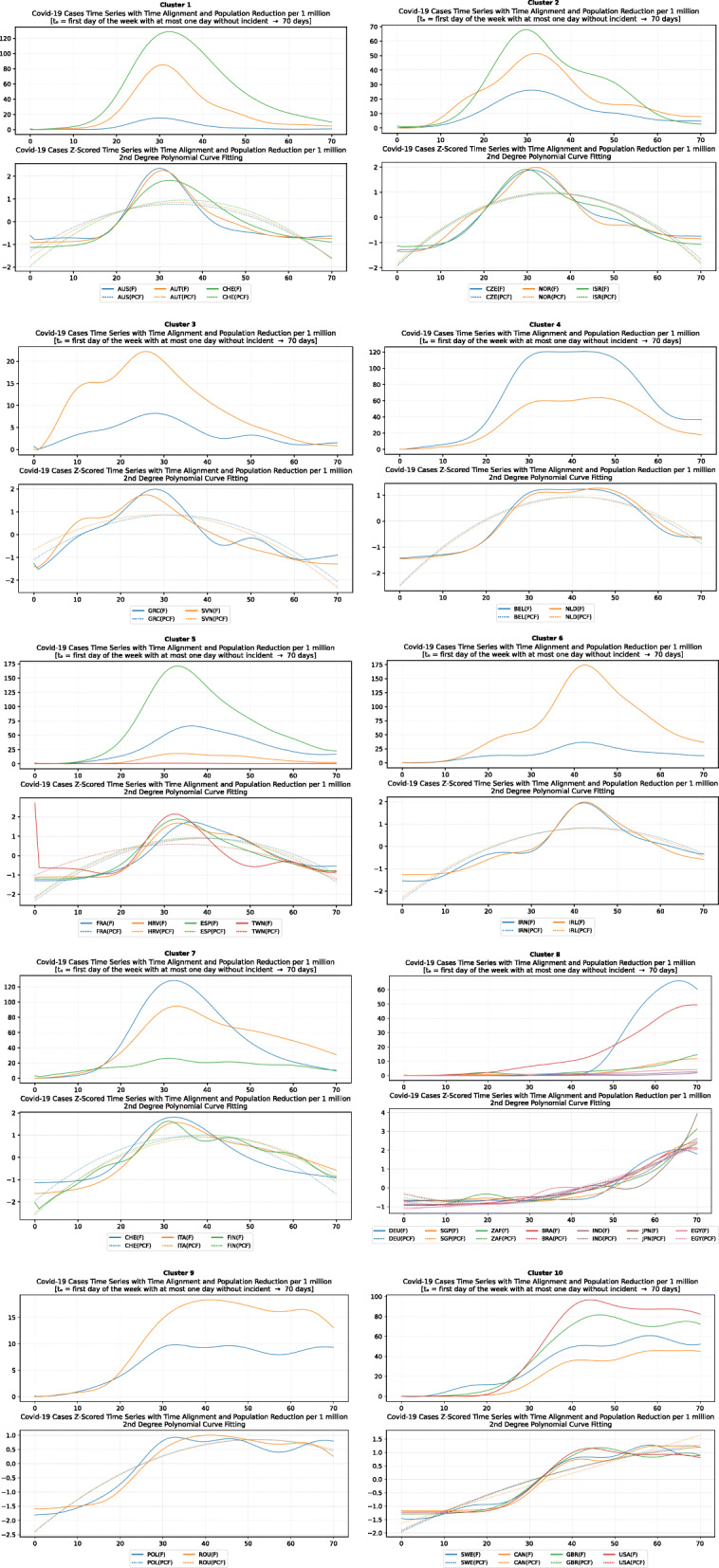
COVID-19 Cases Original/Z-Scored Time Series with Time Alignment and Population Reduction per 1 Million with Second Degree Polynomial Curve Fitting (a)-(j)

Based on curve clustering, there are ten clusters as shown in Fig. 3, where both the original and the standardized (Z-Scored) curves are plotted. Additionally, a second-degree polynomial curve fitting has been performed to show the general trend for each country. Based on polynomial fitting, it can be observed that there are clusters where the countries have practically reduced the cases to zero; this is visible in the first three clusters shown in Fig. 3 (a)-(c). Other clusters, such as six and nine [Fig. 3 (f) and (i)] show countries which have managed to stabilize the infected numbers and started to reduce them. Finally, there are clusters like eight and ten [Fig. 3 (h) and (j)] where the infections continue to show a highly growing pace.

**Fig. 4:**
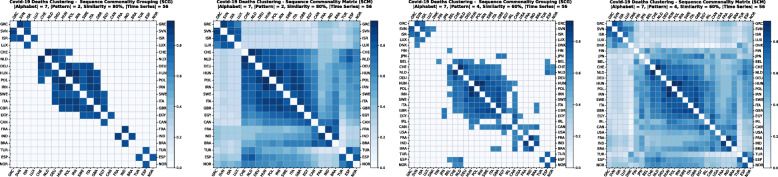
COVID-19 Deaths Clustering – Sequence Commonality Grouping (a) and (c)/Matrix (b) and (d) for 80 and 60% similarity

**Fig. 5:**
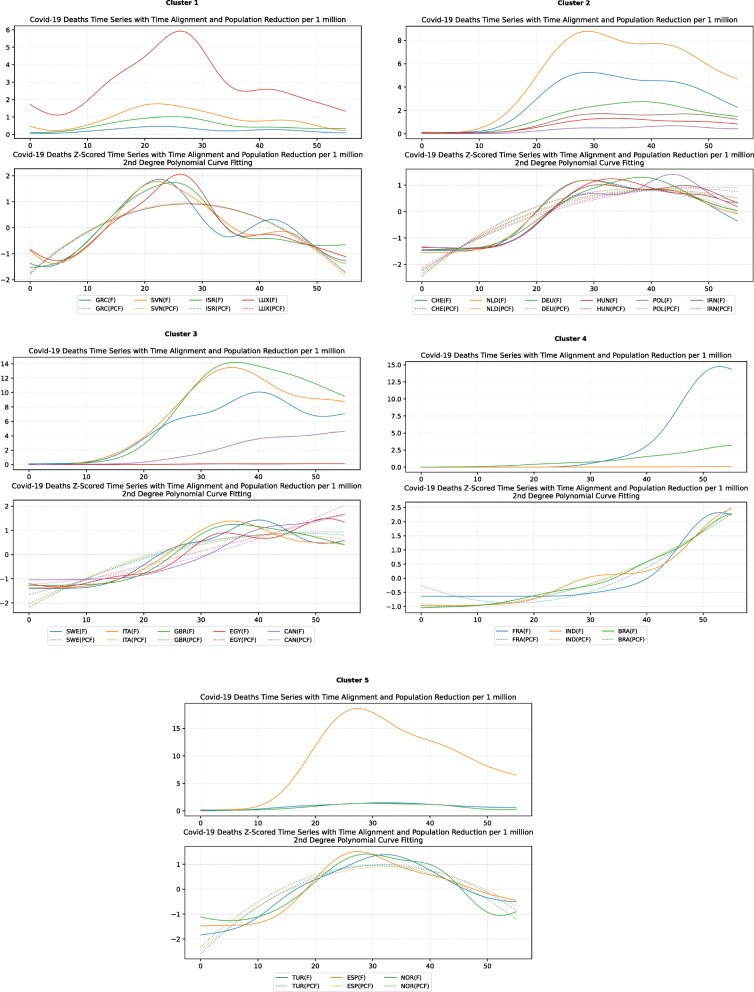
COVID-19 Deaths Original/Z-Scored Time Series with Time Alignment and Population Reduction per 1 Million with Second Degree Polynomial Curve Fitting (a)-(e)

The clusters created based on the death cases time series (Figs. 4 and 5) vary based on the cases (Figs. 2 and 3). This is due to the spread of the first confirmed-death as described earlier. This skews the time series in some cases and accordingly distributed countries into different clusters. Again, we encountered clusters of countries that have managed to practically eliminate deaths, e.g., cluster one shown in Fig. 5 (a). Cluster two shows countries which just managed to stabilize the number of deaths, and potentially they will start to reduce them [Fig. 5 (b)]. Finally, there is cluster four [Fig. 5 (d)] where the increase in the number of deaths is significant; they will need more time to stabilize the process.

### Accumulated cases clustering with DBSCAN algorithm

**Fig. 6:**
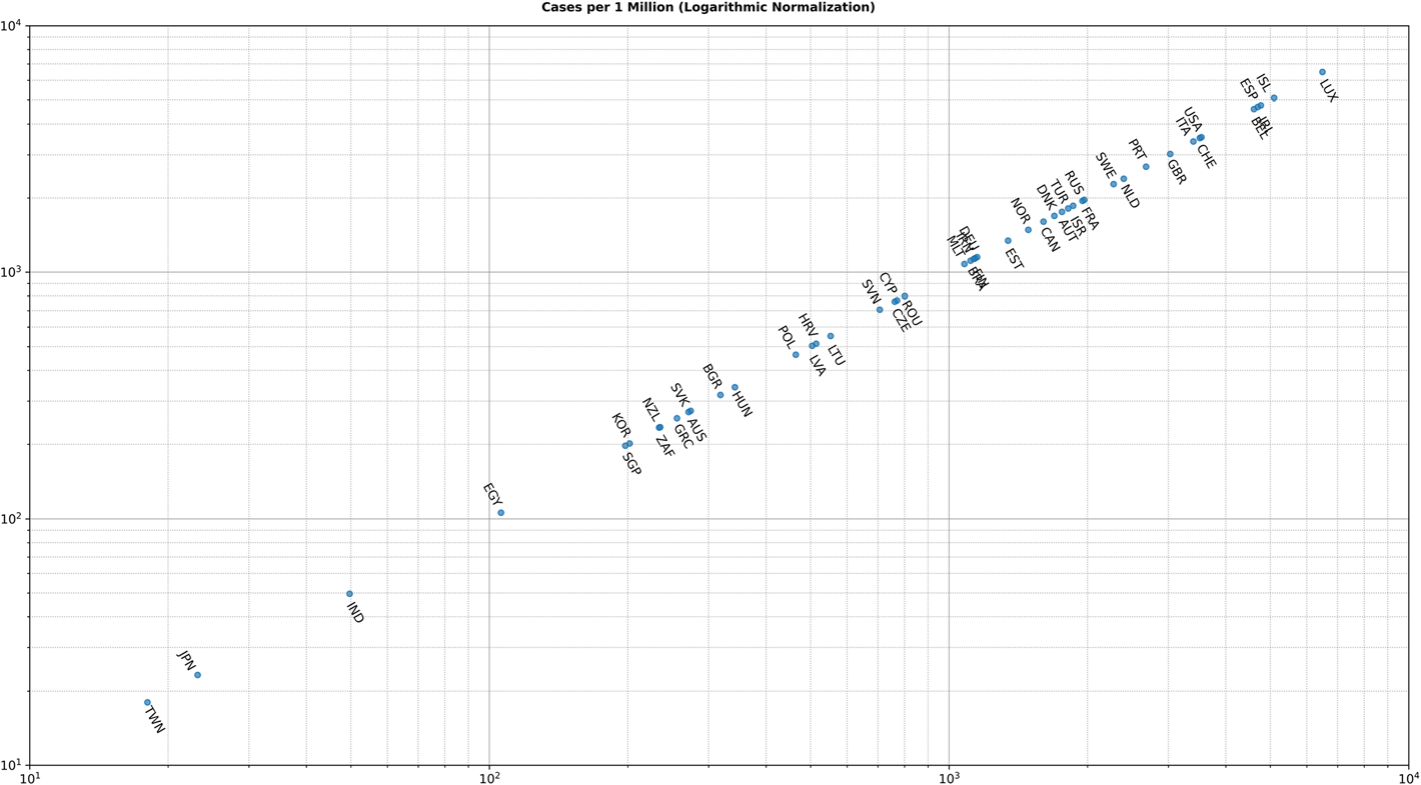
Cases per 1 Million (Logarithmic Normalization)

**Fig. 7:**
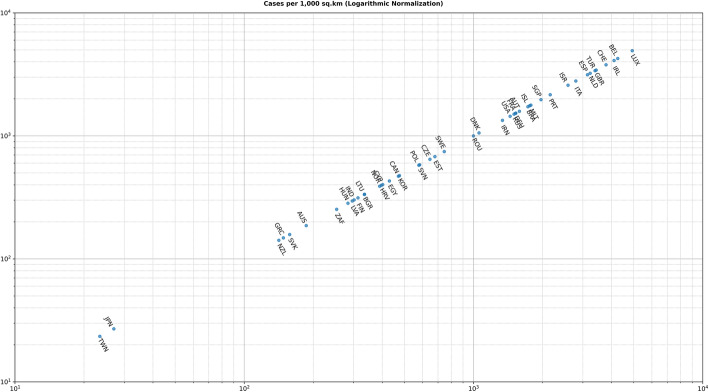
Cases per 1000 Square Kilometers (Logarithmic Normalization)

The second type of clustering is based on the number of cases either per one million of the population or per 1000 km^2^ of the urban land area (1 + 70 days). In most cases, there are no unexpected major differences between the two different approaches. However, there are some very important and noticeable changes as shown in Fig. 6 and Fig. 7. For example, although Iceland is very high in the number of infected cases per one million (5096.43 cases), its ranking has been considerably altered and demoted after using the urban land area (1759.07 cases). Singapore forms another extreme example, while it has a very low ranking per one million (197.56 cases), it ranks very high per urban area (1969.73 cases).

Table 4 reports the results of DBSCAN clustering with epsilon 0.03; it produced 11 clusters and 12 outliers. The DBSCAN algorithm produces the clustering based on the distance of the data points (countries) after normalization as shown in Figs. 6 and 7. The countries in each cluster will be further discussed in the following section to compare the NPI measures implemented by the countries in the same cluster. However, some countries in some clusters will be discussed separately as special cases. For example, Brazil in cluster 3 will not be compared to Iceland and Malta since Brazil is in the southern hemisphere where the disease spread later compared to the north hemisphere. In addition, the outbreak is still in its early stages of evolution with very steep uptrend. Similarly, Egypt and India will not be discussed in clusters 8 and 9 while cluster 11 will not be analysed because both Canada and South Korea were considered special cases. More precisely, South Korea is very close to the origin of the disease. It already has a very strict protocol in place for such outbreaks because it was affected by the previous SARS pandemic in 2002, yet it took very few NPI measures as we will discuss later. Canada, on the contrary, applied all the NPI measures proposed by WHO, however, with considerable delay as it will also be discussed separately as a special case.

**Table 4:** DBSCAN Algorithm Clustering Results for epsilon 0.03

Cluster	Countries
1	BEL, IRL
2	ESP, GBR, NLD, TUR
3	BRA, ISL, MLT
4	AUT, DEU, FRA, RUS, USA
5	DNK, ROU
6	CZE, EST
7	POL, SVN
8	CYP, EGY, HRV, NOR
9	BGR, FIN, HUN, IND, LTU, LVA
10	GRC, NZL, SVK
11	CAN, KOR
Outliers	AUS, IRN, ISR, ITA, JPN, LUX, PRT, SGP, ZAF, SWE, CHE, TWN

### NPIs measures comparison

**Fig. 8:**
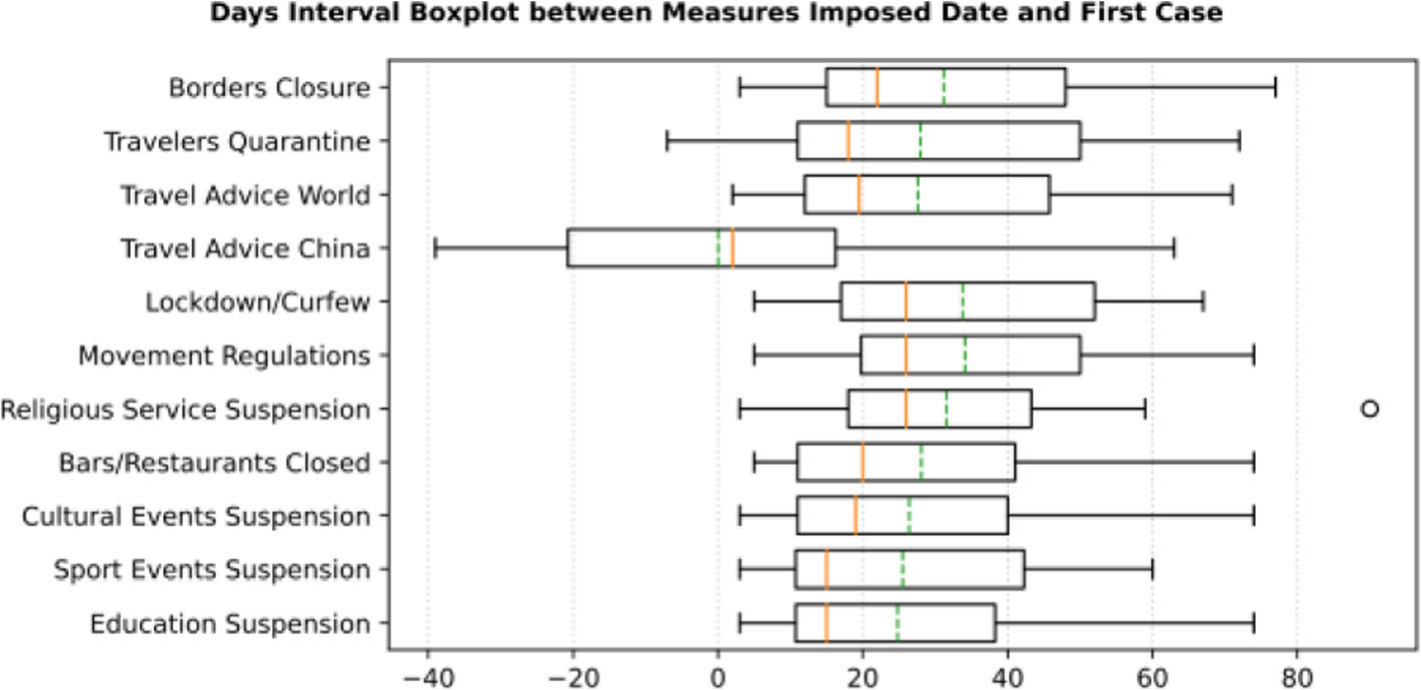
Days Interval Boxplot between Measures Imposed Date and First Case

**Fig. 9:**
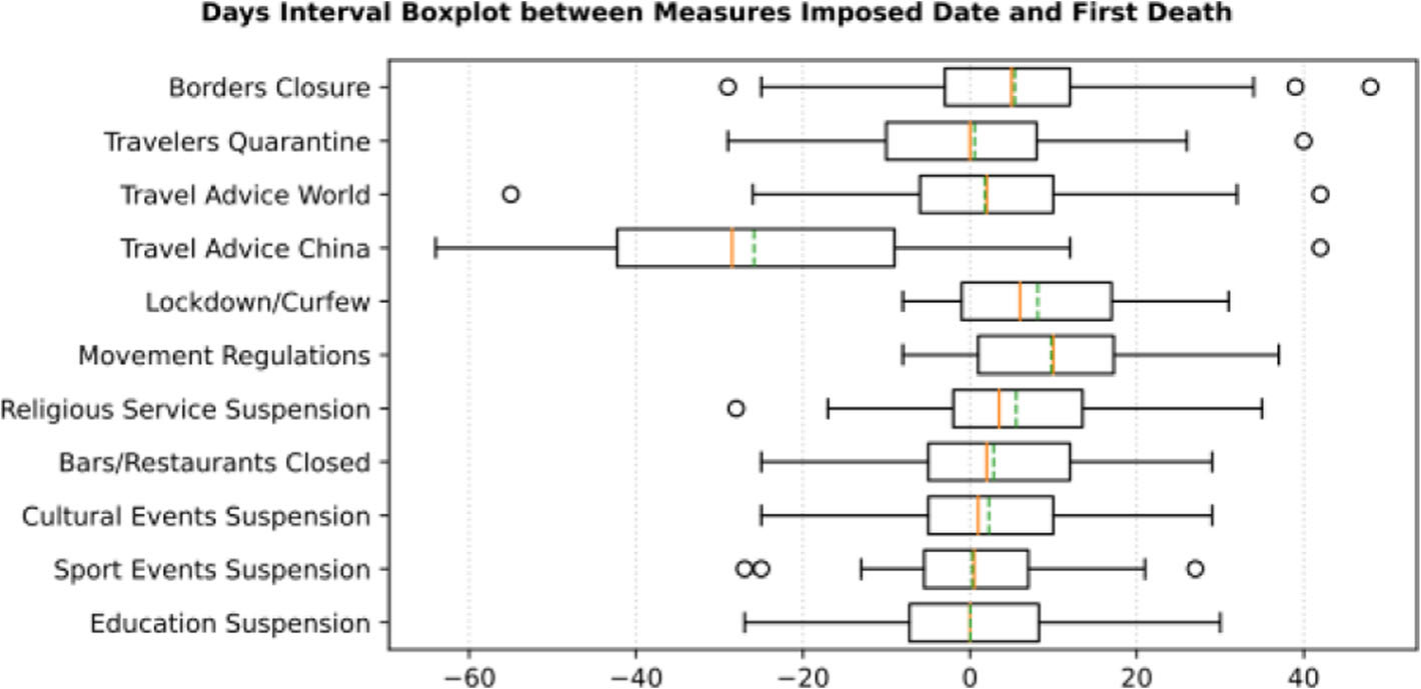
Days Interval Boxplot between Measures Imposed Date and First Death

Based on the manually collected data, the recorded measures are reported in Table 5. For each of the countries considered in this study, the table contains country name, its continent, the date of the first case, and the date of the first death reported according to the world data provided by ECDC [45]. The following columns present for every country the number of days passed from the first confirmed case until each measure was announced. The entry is marked with an “N” in case the country did not announce a measure or in case the specific date was not found during the data collection phase. The next column contains the calculated number of cases per 1000 km^2^ of urban land area on the 18th of May; it is the last day when our data was updated. Finally, the last column presents the cluster of every country where special cases and outliers are denoted by S and O, respectively; this will be discussed in Section 3.2.2. The countries have been sorted by the highest number of cases per 1000 km^2^. This illustrates the impact of the COVID-19 virus on the cases in each country based on the population of urban areas. In addition, Figs. 8 and 9 present a boxplot per measure; they show the interval between the first positive case or the first death and the announcement of the corresponding measure by all the countries in the dataset.

**Table 5:** Number of days after the first reported case per country each NPI measure was taken

Countries	Continent	first case date	first death date	Education Suspension	Sports Events Suspension	Cultural Events Suspension	Restaurants / Bars Closed	Religious Services Suspension	Movement Regulations	Lockdown / Curfew	Travel Advice China	Travel Advice world	Travelers Quarantine	Borders Closure	Cases per Urban 1000 km^2^	Cluster
Singapore	AS	24-Jan	22-Mar	74	49	74	74	56	74	**N**	5	3	56	59	49,575.66	O
Luxembourg	EU	1-Mar	14-Mar	15	14	12	15	12	16	15	−30	15	**N**	22	4924.58	O
Belgium	EU	4-Feb	12-Mar	38	38	38	38	38	**N**	43	25	39	50	45	4476.39	1
Ireland	EU	1-Mar	12-Mar	11	11	23	23	23	26	26	−26	12	35	**N**	4276.42	1
United Kingdom	EU	31-Jan	7-Mar	49	42	**N**	49	46	52	52	33	46	**N**	**N**	4151.62	2
Switzerland	EU	26-Feb	6-Mar	16	5	19	19	27	**N**	**N**	21	16	**N**	28	3862.17	O
Netherlands	EU	28-Feb	7-Mar	17	14	17	17	18	**N**	**N**	**N**	18	**N**	19	3436.31	2
Turkey	AS	12-Mar	19-Mar	4	7	4	4	4	29	**N**	−9	−1	4	16	3389.31	2
Spain	EU	1-Feb	5-Mar	39	40	40	41	41	42	51	**N**	47	**N**	45	3318.81	2
Italy	EU	31-Jan	23-Feb	33	33	36	36	36	49	36	0	30	**N**	57	3065.44	S
Germany	EU	28-Jan	10-Mar	45	47	54	54	54	55	54	**N**	49	**N**	47	2800.79	4
Israel	AS	22-Feb	21-Mar	19	19	22	22	22	32	32	−23	4	16	25	2596.80	O
Portugal	EU	3-Mar	18-Mar	9	7	10	10	10	17	17	**N**	9	17	13	2274.71	O
Malta	EU	7-Mar	9-Apr	5	5	9	9	15	**N**	**N**	−10	12	5	3	1885.18	3
United States of America	AM	21-Jan	1-Mar	70	50	**N**	**N**	59	**N**	67	10	51	**N**	59	1853.69	4
Brazil	AM	26-Feb	18-Mar	**N**	**N**	**N**	**N**	**N**	**N**	**N**	**N**	33	20	20	1786.02	S
Iceland	EU	29-Feb	20-Mar	13	13	24	24	**N**	**N**	**N**	−36	19	18	17	1760.05	3
Iran	AS	20-Feb	20-Feb	3	3	3	**N**	25	**N**	**N**	**N**	**N**	**N**	**N**	1735.89	S
France	EU	25-Jan	15-Feb	51	48	50	50	50	58	52	63	51	**N**	52	1647.07	4
Austria	EU	26-Feb	13-Mar	13	15	19	19	19	19	19	13	18	19	19	1644.56	4
Russia	AS	1-Feb	27-Mar	51	45	45	52	75	58	52	19	46	47	51	1502.37	4
Denmark	EU	27-Feb	16-Mar	15	14	15	20	20	**N**	13	−20	15	11	16	1175.56	5
Romania	EU	27-Feb	23-Mar	13	15	18	18	23	23	23	30	15	11	24	1081.84	5
Sweden	EU	1-Feb	12-Mar	**N**	47	**N**	**N**	**N**	**N**	**N**	16	42	**N**	47	967.60	S
Estonia	EU	28-Feb	26-Mar	16	16	24	20	22	**N**	27	−33	17	32	20	678.48	6
Czech Republic	EU	2-Mar	23-Mar	8	11	11	11	11	13	13	−23	15	11	11	676.05	6
Canada	AM	26-Jan	10-Mar	52	47	52	52	52	**N**	52	3	47	59	52	608.57	S
Poland	EU	4-Mar	13-Mar	8	8	8	8	27	20	8	−39	11	11	11	607.48	7
Slovenia	EU	5-Mar	18-Mar	11	11	11	11	11	15	**N**	−27	25	**N**	12	584.26	7
South Korea	AS	20-Jan	21-Feb	34	35	35	**N**	41	**N**	**N**	**N**	56	72	**N**	504.74	O
Egypt	AF	15-Feb	9-Mar	27	27	27	27	39	38	38	20	28	**N**	31	503.87	O
India	AS	30-Jan	13-Mar	46	43	49	49	49	54	54	−9	43	54	48	431.85	O
Croatia	EU	26-Feb	25-Mar	19	15	20	20	20	26	20	23	41	16	22	419.78	8
Norway	EU	27-Feb	13-Mar	15	14	14	14	16	**N**	14	−10	16	19	18	404.15	8
Cyprus	EU	10-Mar	25-Mar	3	6	6	6	14	13	13	6	6	6	5	401.68	8
Bulgaria	EU	8-Mar	12-Mar	5	5	5	5	**N**	5	5	9	7	**N**	12	334.63	9
Lithuania	EU	28-Feb	21-Mar	14	**N**	14	**N**	**N**	**N**	**N**	−36	13	**N**	17	334.21	9
Finland	EU	30-Jan	22-Mar	46	47	46	46	48	**N**	**N**	43	42	46	49	316.52	9
Latvia	EU	3-Mar	4-Apr	10	**N**	**N**	**N**	26	**N**	**N**	−32	10	9	14	307.46	9
Hungary	EU	5-Mar	16-Mar	11	6	11	**N**	**N**	23	**N**	6	5	6	11	299.76	9
South Africa	AF	6-Mar	27-Mar	12	20	20	20	20	20	20	9	9	**N**	20	290.22	O
Australia	OC	25-Jan	1-Mar	**N**	57	58	58	58	64	64	7	48	51	55	191.72	O
Slovakia	EU	7-Mar	7-Apr	6	6	6	6	3	32	**N**	−9	5	9	6	159.65	10
Greece	EU	27-Feb	12-Mar	12	12	14	14	18	25	25	−14	20	18	19	153.03	10
Japan	AS	15-Jan	13-Feb	47	42	45	**N**	**N**	**N**	**N**	17	71	29	77	150.03	S
New Zealand	OC	28-Feb	29-Mar	24	26	26	26	26	26	26	−26	26	15	20	141.57	10
Taiwan	AS	21-Jan	17-Feb	12	37	**N**	**N**	37	**N**	**N**	−16	59	53	66	24.05	S

### General observations

An interesting observation reflected in Table 5 is related to the number of cases per 1000 km^2^. Singapore appears in the top 10 rows of the table with a very high number of cases which can be easily explained since Singapore has one of the highest population densities worldwide with 7804.40 people, and its surface area is 723 km^2^. As a small island, Singapore has been developed as an urban area, and thus the population per urban area is higher and equal to 9970.08 persons per square kilometer. Consequently, the 49,575.66 cases per 1000 km^2^ is not a surprise. It is rather an expected outcome due to the highly dense populated area. The second country in the list is Luxembourg which is a very small country with 801 km^2^ surface area, but with a very lower population density compared to Singapore. Accordingly, the number of cases per 1000 km^2^ is lower than that of the first country on the list. The rest of the countries in the top 10 list seem to have taken measures very late except Turkey; it is a special case which will be discussed later in the cluster analysis section. Comparing the measures taken by countries, the majority of them did not quarantine incoming travelers or did that very late, e.g., Belgium and Ireland. Some other countries, such as Switzerland, Netherlands and Belgium did not impose restrictions on movement, or they imposed restrictions only in specific high-risk areas. It is also interesting that all countries are in Europe except Singapore and Turkey.

Studying now the last 10 rows of the table in terms of the number of cases per 1000 km^2^ of urban areas, it appears that the majority of these countries are not in Europe. However, many of them have issued a travel advice against traveling to China several days before the first reported case which seems to be effective, since the first case was reported several weeks after the travel advice. Another remarkable observation is that most of these countries are neighbors of China which means they have probably capitalized their previous experience from the coronaviruses of SARS in 2002 and MERS in 2012 that affected Asia and Arabic Gulf correspondingly. Taiwan, New Zealand, Japan and Australia are very close to China and very early responded to the possibility of incoming cases of contaminated travelers from China. Even countries that did not issue travel advice against traveling to China such as Japan, very early (from early February) took other preventive measures such as passenger screening on arrival in the main entrances of the country [59].

Cross-analysis of the findings from Table 5 and the two boxplots presenting the days interval between the date of the first infected case reported and the date of the first death reported (Figs. 8 and 9, respectively), very interesting conclusions may be drawn regarding the response of all the analyzed countries to COVID-19 pandemic. More specifically, it appears that the most widespread restriction measure that was taken too early was the issuing of travel advice against traveling to China. Checking the mean (green dotted line) and the median (red solid line) in Fig. 8, we can see that most countries took the specific measure when the first case in their country was reported, while some countries delayed few days since the median comes after the mean. This can be attributed to the special relationship between specific countries and China as it can be seen in Table 5 and Fig. 12, where France seems to have issued a travel advice against traveling to China very late in comparison to the other EU countries. However, this could be correlated to the fact that there is a very large French student community of about 10,000 students, the largest student group from Europe in China, and these students were given the option to return to their families in France [60]. Similarly, in Fig. 9 where the first death cases from COVID-19 are illustrated against dates of implementing the specific measures, it is obvious that very few countries issued the specific travel advice after the first death. It is apparent that such a measure is totally justified by the fact that China was the origin of SARS-COV-2, and as the virus was proved to be extremely contagious, majority of the countries issued the specific travel advice very early, even though WHO issued a travel advisory for China on the 24th of January 2020. They updated the advisory on the 27th of January 2020 without explicitly recommending avoiding traveling, but instead recommending entry and exit screening and not travel restrictions: “*WHO advises against the application of any restrictions of international traffic based on the information currently available on this event.*” [61, 62].

Focusing on Fig. 8, it can be seen that the batch of NPI measures related to social distancing such as closure of bar/restaurants, suspensions of cultural and sports events are almost taken by the majority of the countries where each country implemented the measures two or 3 weeks after its first confirmed infected case or just after its first death case was reported (see Fig. 9). Another noteworthy point is that sport events and education suspension seem to have been decided as preventive measures for 50% of the countries less than a week before or less than a week after the first death (Fig. 9). If we compare the specific measures with the dates of the first confirmed case which appeared in a country (Fig. 8), it can be easily realized that all countries waited a week or more to apply these measures. More interestingly, the first 50% of the countries that took the measures imposed the specific measures in a range of 10 days, while the rest of them adopted the measures with a delay of at least 4 weeks. Regarding European countries, this can be easily justified because Italy and Spain were initially affected by the pandemic and all the rest made their decisions around mid-March when it was evident that the pandemic was out of control in these two countries. During the same period, the European Union leaders under the auspices of the European Commission decided on a coordinated response against the pandemic. This justifies why most of the measures were implemented at the same period [63].

Figures 8 and 9 cofirm the further delay in the implementation of movement restrictions or complete lockdown by all countries. As mentioned earlier, restriction on movement is a rather strict measure which is sometimes considered to limit personal freedom. Therefore, many countries had second thoughts before applying such restrictive measures. As a final note on the analysis of the measures regarding their implementation date in comparison with the first day of a confirmed infected case or a death case in a specific country, it is interesting to point out that religious service suspension was applied at the same period when movement restrictions or lockdown were decided, if applied at all.

**Fig. 10:**
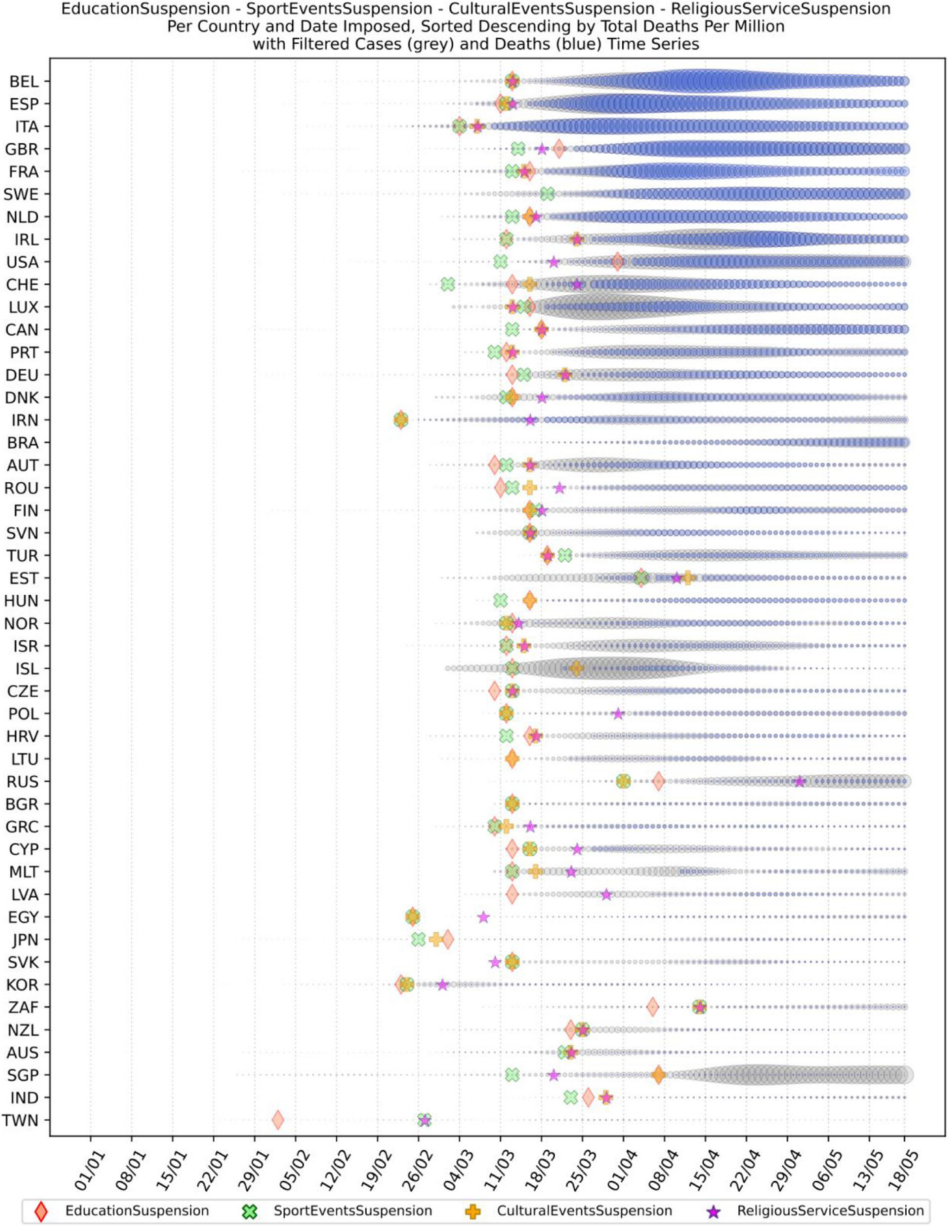
Educational measures and workplace and public place measures

**Fig. 11:**
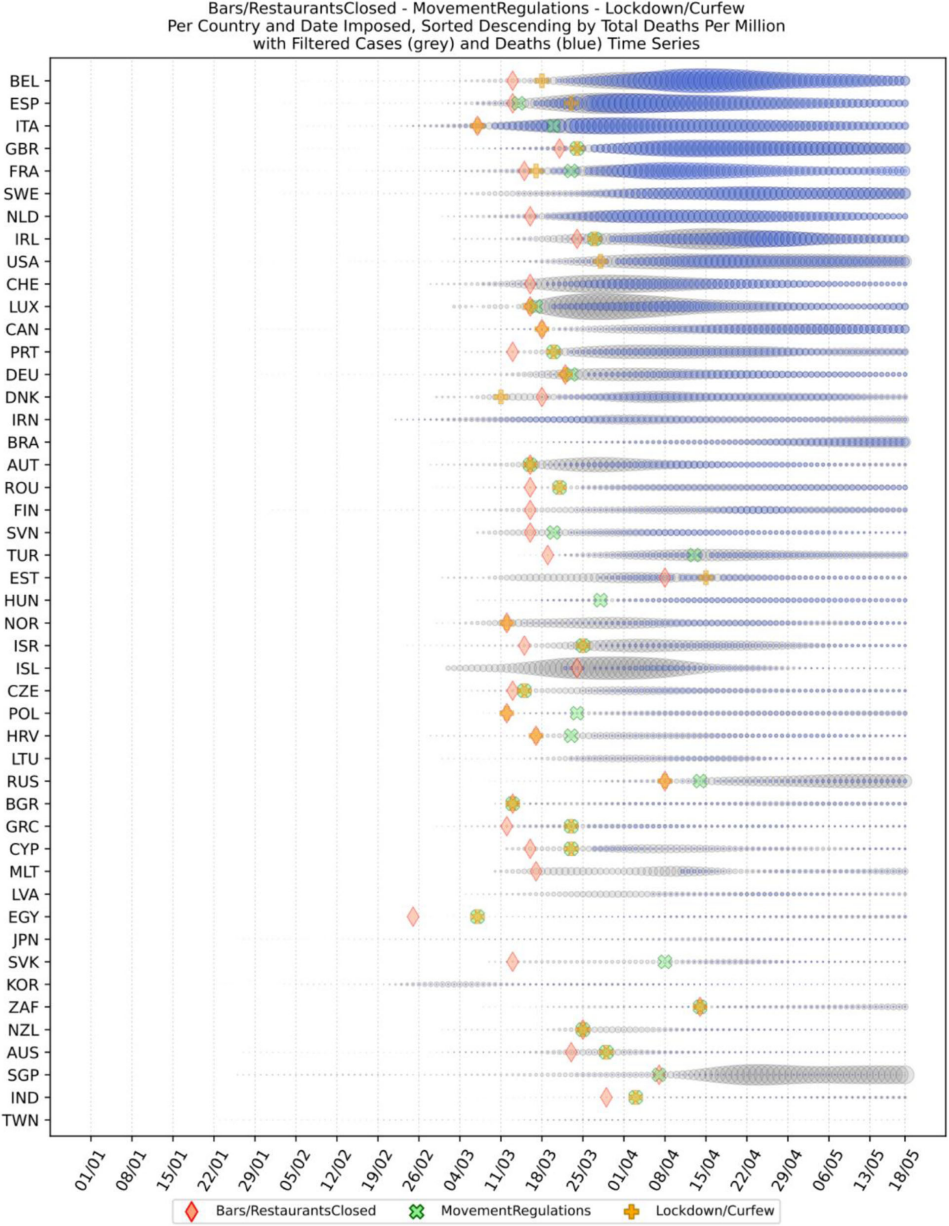
Social distancing and closure of restaurants

**Fig. 12:**
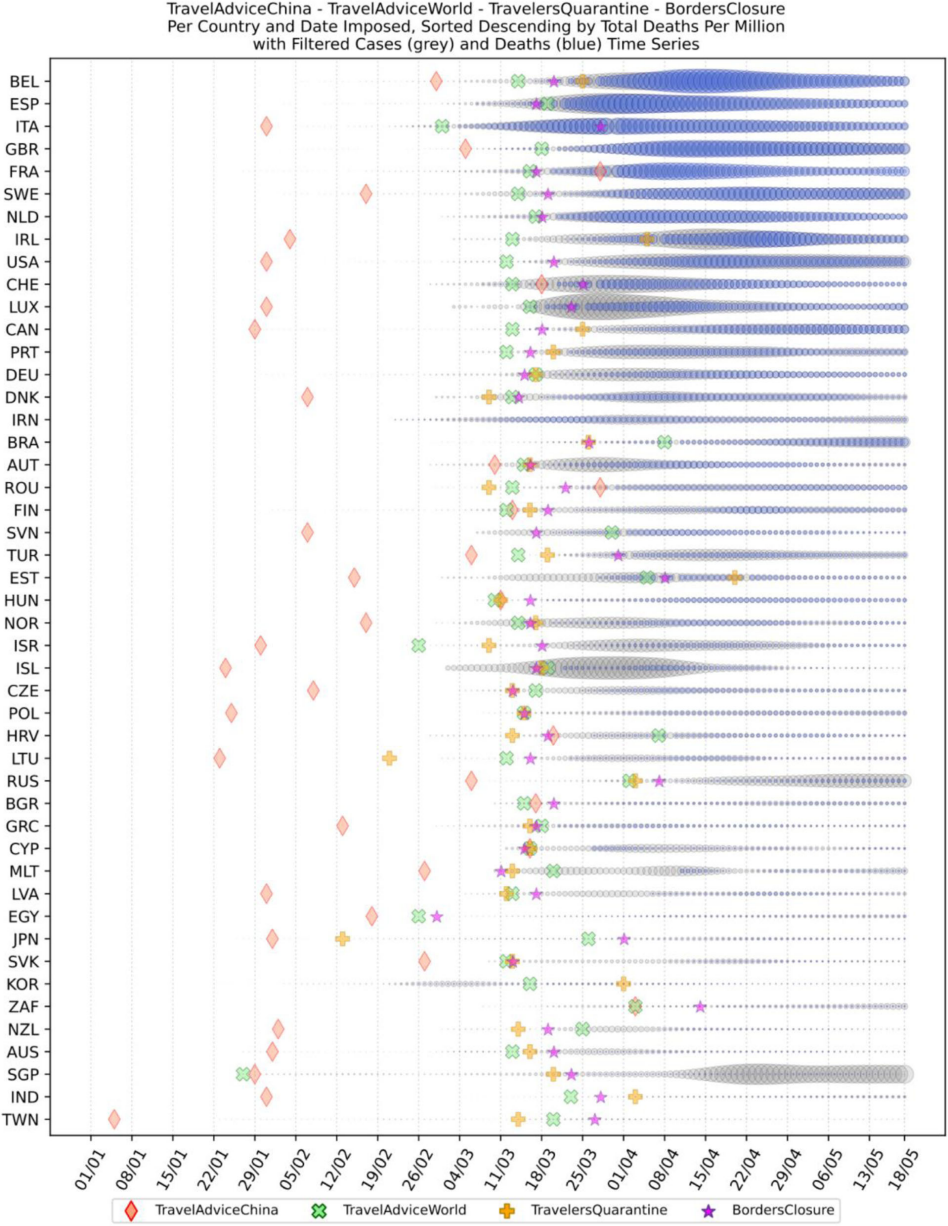
Travel measures

The list of implemented measures per category and country can be seen in Figs. 10, 11, 12. The countries in the diagrams have been sorted by the total number of deaths per one million of the countries’ population. This also illustrates the dates when each measure was taken, and the trend of the daily number of infected and death cases reported over the period from January 1st to May 18th. Figure 10 presents the dates when schools and universities were closed, and cultural and sports events as well as religious services were suspended. All these measures belong to the two categories of measures proposed by WHO in response to a pandemic crisis under educational, workplace and public place measures. Another noteworthy observation in the specific figure is that the suspension of sport events is the first measure that was applied almost by all the considered countries. This is important because in sport arenas usually a large crowd gathers in a very close distance, and thus it becomes hard to avoid the transmission of a respiratory disease.

The next more popular measure imposed by most countries after closing sport arenas was the closure of schools and universities. This is mainly justified, because in case of schools, young students are not guaranteed to apply and respect social distancing as needed, and thus it would be better to stay at home. For university students, class attendance usually entails commuting and socializing before and after the classes, and thus it becomes easier to be exposed to the virus. As depicted in Fig. 10, from the analyzed countries, Sweden, Brazil, and Australia did not close their schools and universities at least by the time this paper was written. In Sweden the absence of measures seems to have affected the number of total infected and death cases since it appears that these numbers are relatively higher than other countries which took the specific measure. For Brazil, where the government did not take any national measure, the number of cases and deaths was initially low, but progressively seems to have increased rapidly. Australia which did not close schools and universities, seems to have no significant increase in the number of deaths and infected cases because schools and universities academic year was planned to start in early February [64]. However, all the other measures for suspension of public events and gatherings were applied relatively early. These may have affected the total number of infected and death cases in Australia.

Another remarkable point that can be observed in the specific diagram is the different times that the countries decided to suspend the religious services if at all suspended. Several countries, such as Sweden, Brazil, Hungary, Iceland, Lithuania, Bulgaria, and Japan did not suspend the religious services. This is attributed either to the political disposition of a country against COVID-19 as in the case of Sweden and Brazil, or to the political power of the local church as in the case of Hungary and Bulgaria. Another group of countries waited for more than 15 days from the first reported death to suspend religious services, e.g., Russia, France, Iran, Australia, USA, Switzerland, Poland, South Africa, India, and Egypt. Other countries such as Italy, Ireland, Germany, United Kingdom, Netherlands, Taiwan, and South Korea suspended religious services in the second week after the first reported death case. The third group of countries, namely Spain, Canada, Greece, Austria, Denmark, Belgium, Norway and Turkey decided to suspend religious services in the first week after the first reported death case, while the rest 14 countries suspended religious services a week or more earlier than the first reported death case. There is evidence that religious services contributed to the rise of the numbers of COVID-19 infections in South Korea. Indeed, it was reported in early March more than 2000 cases originated from a congregation meeting held in Daegu [65]. However, as it can be understood by the late suspension of the religious services in most countries, there were political implications, and the suspension came after the other NPI measures.

A second batch of measures is related to social distancing and the closure of public places where people usually gather, such as bars and restaurants. The dates when the restaurants and bars were closed in the analyzed countries are presented in Fig. 11 along with the dates when the countries decided to enact lockdown or pose movement restrictions. An interesting observation is that the closure of bars and restaurants preceded by one or 2 weeks the restrictions on movement or general lockdown in most of the countries.

A very representative example of late decision for either restrictions of movement or lockdown is the cases of Italy and Spain. From the diagram, it is apparent that in Spain the regulation on movements at a national level started 10 days after the decision of the closure and after the lockdown had been decided by the government. Similarly, while the closure of bars and restaurants in Italy was decided very late, i.e., 2 weeks after the first reported death case, the movement restriction was imposed 26 days after the first reported death case. The delay seems to be analogous to the number of infected cases who need to be hospitalized, and as a result many from those people did not make it since the hospitals were already full.

Another interesting case is Belgium where there was no restriction on movement, however, the restaurants and bars were closed at the same time when all the other NPI measures were taken. The lockdown was decided a week later without imposing any restrictions on movement. Judging by the number of infected and death cases due to COV-SARS-2 in Belgium, it seems that not getting any restriction on movement led to a very large number of contaminations, and consequently to a very large number of death cases. Belgium, however, is a particular case since there are several European Union agencies and offices in Brussels. Thus, a lot of people travel from all over Europe to Belgium for meetings. That seemed to have happened at the beginning of the pandemic in early March when people from Italy and Spain travelled to Belgium without yet knowing that they were contaminated with the COV-SARS-2 virus.

Other countries which did not take any restrictions on movement are all placed in the top 10 of the list with larger number of deaths per million, e.g., Sweden, Netherlands, Switzerland and United States. Similarly to Italy, the United States started enacting local lockdowns in regions or states, and as shown in the diagram (Fig. 11) this measure was not enough to restrict the contamination. On the contrary, this caused the widespread of the virus to other areas. Though in Italy there was a different approach than in the United States; the government decided to put restrictions on movement a month after the first reported death case. As a result, the number of contaminations started to decline progressively. This did not happen in the United States, and it appears that even during the days when this paper was written there has been a constant rate of contamination without any reduction in the number of new infected or death cases in the country. Another interesting observation in Fig. 11 is that some countries such as South Korea, Japan and Taiwan did not take any of these social distancing measures. However, there was no increase in the number of cases or deaths in these countries. It is obvious that the other measures taken were very effective and did not allow the widespread of the virus. It is also argued that the population mentality of using personal protective equipment such as masks in these countries played an important role in controlling the spread of the virus [66]; the success is attributed to border control measures as well; these are discussed next.

It is also interesting to observe how countries have imposed travel measures in the era of COVID-19. From Fig. 12, it appears that a very small number of countries did not issue a travel advice against traveling to China; these are Brazil, Iran, Germany, Netherlands, South Korea, Spain, and Portugal. From these countries, only Iran seems not to have imposed any travel restrictions, while others delayed two or more weeks to implement such a measure. The latter countries include Japan, Taiwan, France, South Korea, Brazil, and Spain. The combination of all the measures taken in some of these countries seem to be effective, e.g., Japan, South Korea, and Taiwan. For other countries like France, Brazil, and Spain, the combination of the measures and the dates taken seem to have led to an uncontrolled spread of COVID-19.

Another measure that was suggested by WHO in the batch of measures intended to reduce the impact of a pandemic is to put on quarantine for two weeks all passengers coming from other countries. The period of two weeks is not random, and it is specifically recommended for COV-SARS-2 because this is the incubation period of the virus as reported by WHO [67]. Most of the countries applied such a measure a week or after the first reported death case in the country. However, some countries like Iran, France, Spain, Slovenia, United Kingdom, United States, Netherlands, Switzerland, South Africa, Italy, Bulgaria, Sweden, Luxembourg, and Estonia did not apply such a measure. In many cases, this is justified by the fact that some countries closed their borders at the same time, and thus they did not accept incoming travelers. However, all countries organized repatriation flights for expats and that may have affected the local spread in the country if no strict quarantine measures were followed by returning citizens. The effectiveness of this measure lies on how it is applied. If for example, there is only a recommendation for an individual travelling back home to stay 14 days in quarantine without monitoring, then it is very possible that he/she may violate the quarantine and infect local people in case of contamination.

Border closure was another travel measure that was taken, however, not so early as it should have been, and in some cases, it was not considered at all. For example, Iran, South Korea, United Kingdom, and Ireland did not close their borders. Each of these countries has its own special reasons. UK and Ireland are two countries which share the same border and are isolated from the rest of the world. Thus, it is easier to screen entrance and exit to such countries since the only way to travel is by sea or by air. At their common border, however, there was no control while allowing the transfer of their citizens across the border. Based on the total number of cases per 1000 km^2^, both countries paid a high toll of 4276.42 and 4151.62 cases, respectively. Similarly, South Korea as an isolated country, being in the Korean peninsula, was also able to screen incoming travelers easily. Further, analyzing countries that lately closed their borders, in Europe Switzerland, Italy and France delayed border closure from twenty days to one month from the day of the first reported death. This might have been a main reason for very high number of deaths per million. Other islandic countries that delayed border closure seem not to be affected by that, e.g., Japan, Australia, and Taiwan; this can be again attributed to the screening measures at the airports and seaports.

Studying all travel measures together, the top 10 countries with the higher number of deaths per million did not put in quarantine incoming travelers except for Ireland and Belgium which took the measure but delayed by 24 and 13 days, respectively. With the evolution of the disease and the impact on the deaths per million, the quarantine of incoming travelers seems to be the most effective measure in the specific batch.

**Fig. 13:**
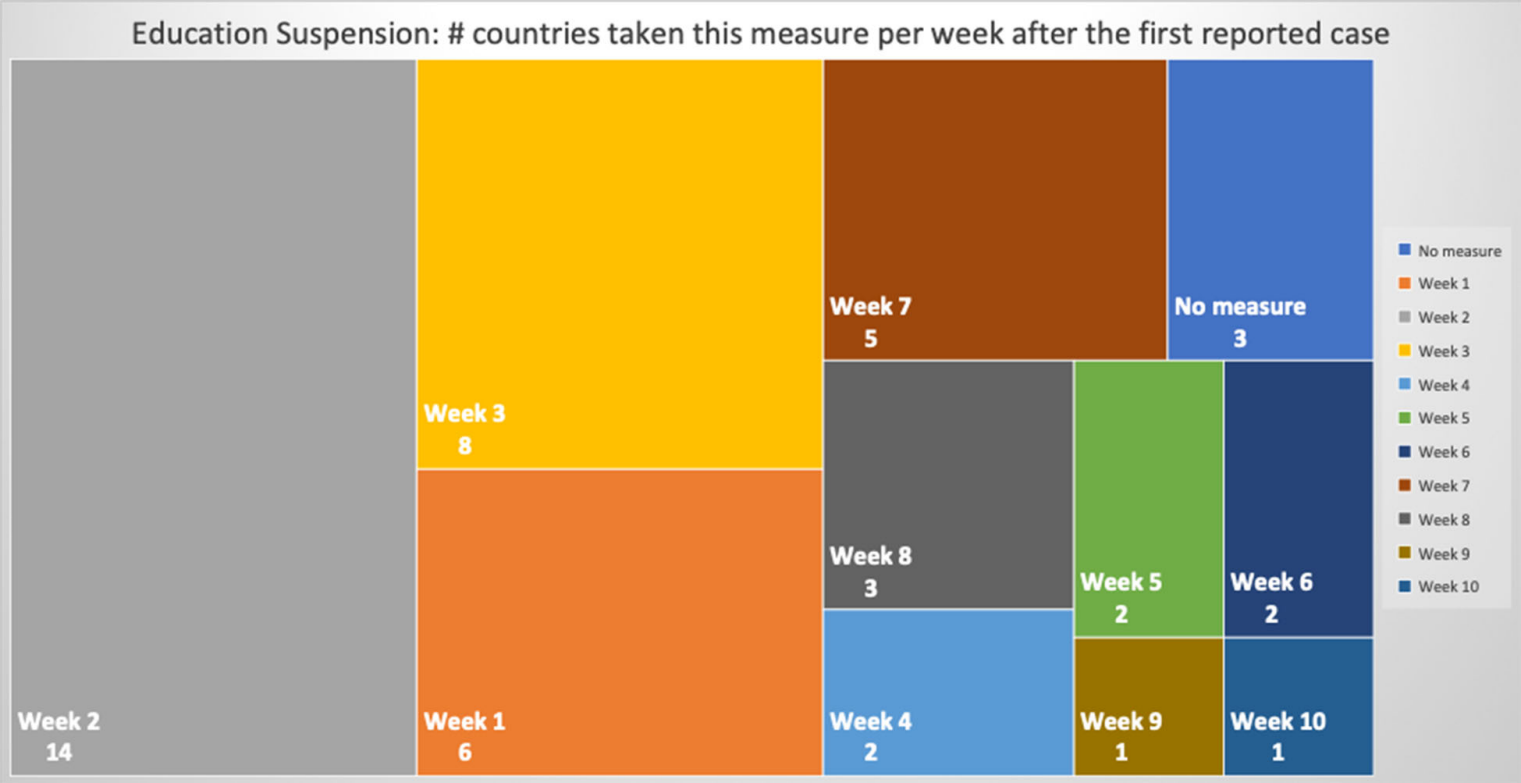
Number of countries per week which enabled education suspension

Further analyzing the NPI measures, and more specifically illustrating the number of countries versus the weeks when they took the measures after the first reported case, we can see how quickly these countries responded to the pandemic. In Fig. 13, we can see that more than half of the countries (28 out of 48) closed schools and universities in the first 3 weeks after the appearance of the first COVID-19 case. Most of them (14) took the measures in the second week while only six responded fast and took the measures in the first week.

**Fig. 14:**
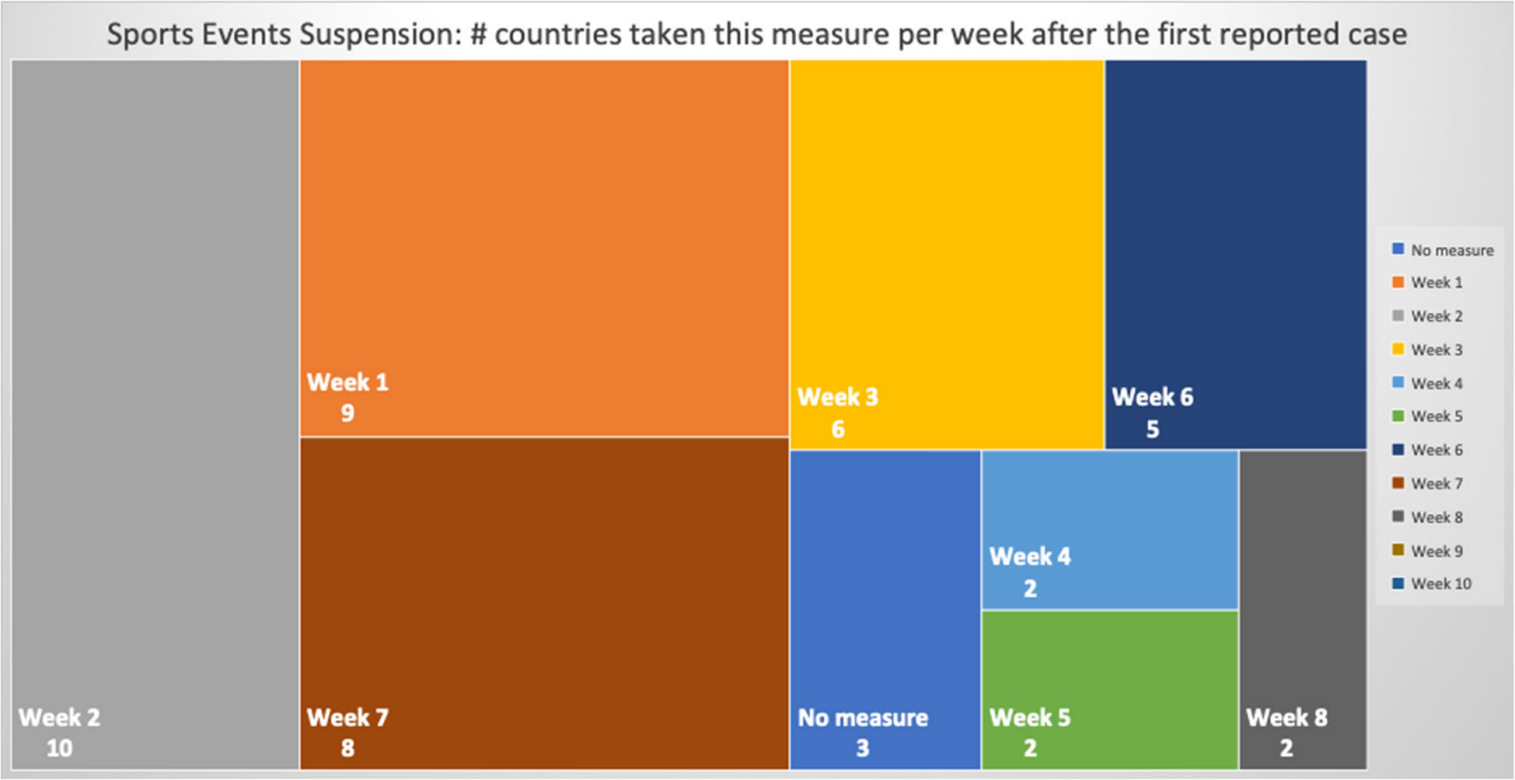
Number of countries per week which enabled sports events suspension

**Fig. 15:**
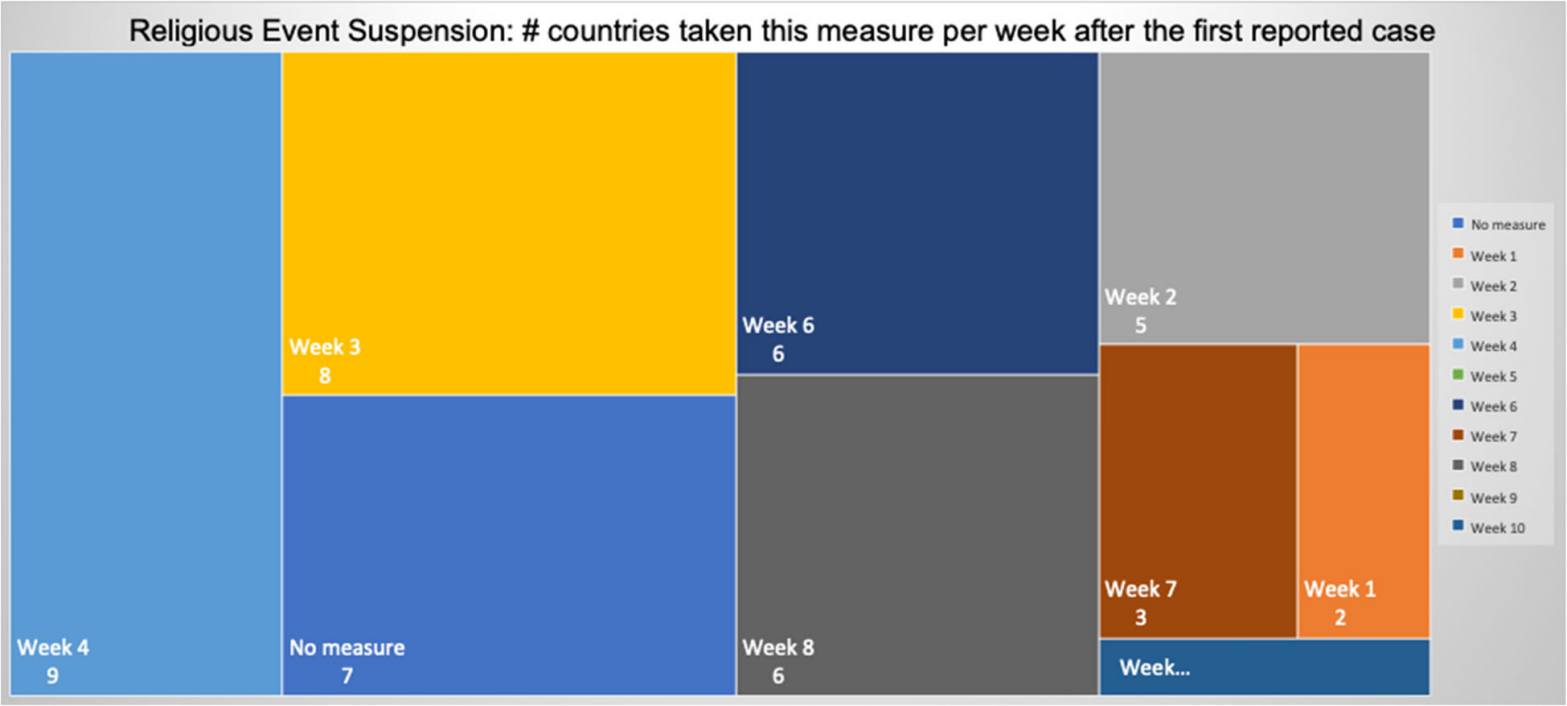
Number of countries per week which enabled religious events suspension

Figure 14 shows that 19 countries suspended sports events in the first 2 weeks after their first reported case of the virus; this means that sports events suspension was considered by these countries an important measure that could be easily applied at an early stage. In Fig. 15, religious events suspension is illustrated against the weeks that the measure was applied. Here, it is evident that the decision to suspend religious services came after the previously mentioned measures. We can observe that most of the countries took this measure very late and more specifically after the third week of their first case of COVID-19. Another important finding is that 7 countries did not suspend religious services at all since as mentioned before is a rather sensitive issue.

**Fig. 16:**
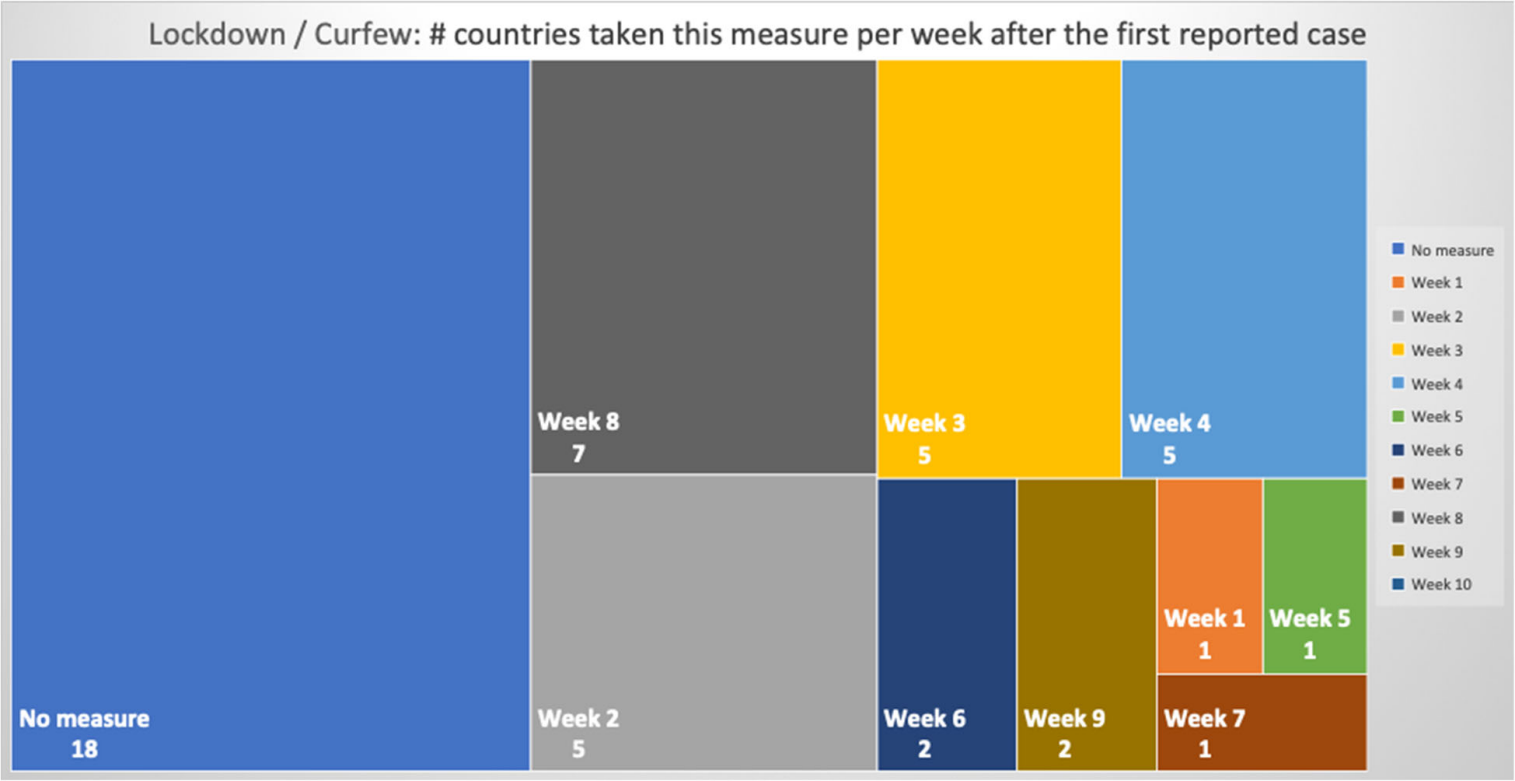
Number of countries per week which enabled lockdown

It is also interesting to study Fig. 16 which presents the lockdown measures applied based on the week applied. Many countries (18) did not apply any national lockdown measure, and the rest of the countries which took the measure were rather late. From the above, we can conclude that most of the governments followed a varied approach in applying the measures recommended by WHO in their effort to contain the COVID-19 pandemic. Most of the time, it seems that their decisions were possibly based more on the potential economic cost associated with a measure, e.g., the lockdown or the political cost as in the case of religious services suspension that were taken very late after the other social distancing related measures.

### Cluster analysis based on NPIs measures

As mentioned in the methodology section, besides the time series clustering which mainly presents the trends and classifies the countries based on them, a second clustering has been performed based on the number of infected cases per 1000 km^2^ of the urban land area. The clusters of the latter analysis will be presented in this section. It is important to mention that we only care for confirmed infected cases and not death cases because the NPI measures affect the spread of the disease while death cases could be correlated to other factors like the number of ICUs, available pharmaceutical supplies, health care personnel, etc. Besides, the NPI measures are essential to avoid the transmission because after the contamination of a person, the evolution of the personal health state of a contaminated person is irrelevant to the NPI measures. In other words, it is very interesting to investigate whether there are significant similarities or differences in the NPI measures applied by the various countries in the same clustered.

As it was previously mentioned, the clustering mainly depicts the number of cases per 1000 km^2^ in the urban areas of the countries. The specific type of case reporting has been considered since as mentioned in the previous section, large countries such as Canada and Australia have very low density by considering their total land area. However, most of their population live in the largest cities such as Toronto, Montreal, Vancouver, and Calgary in Canada or Sydney, Melbourne, and Brisbane in Australia. Thus, the cases were projected to the 1000 km^2^ of the urban land area, and not the absolute number of cases. Consequently, we are interested in the way that the cases developed in line with the NPI measures taken after the appearance of the first case in each country. In the rest of this section, we will discuss the countries in each cluster separately to comment on their specific decisions regarding the NPI measures and to investigate their commonalities.

### Cluster 1: Belgium and Ireland

**Fig. 17:**
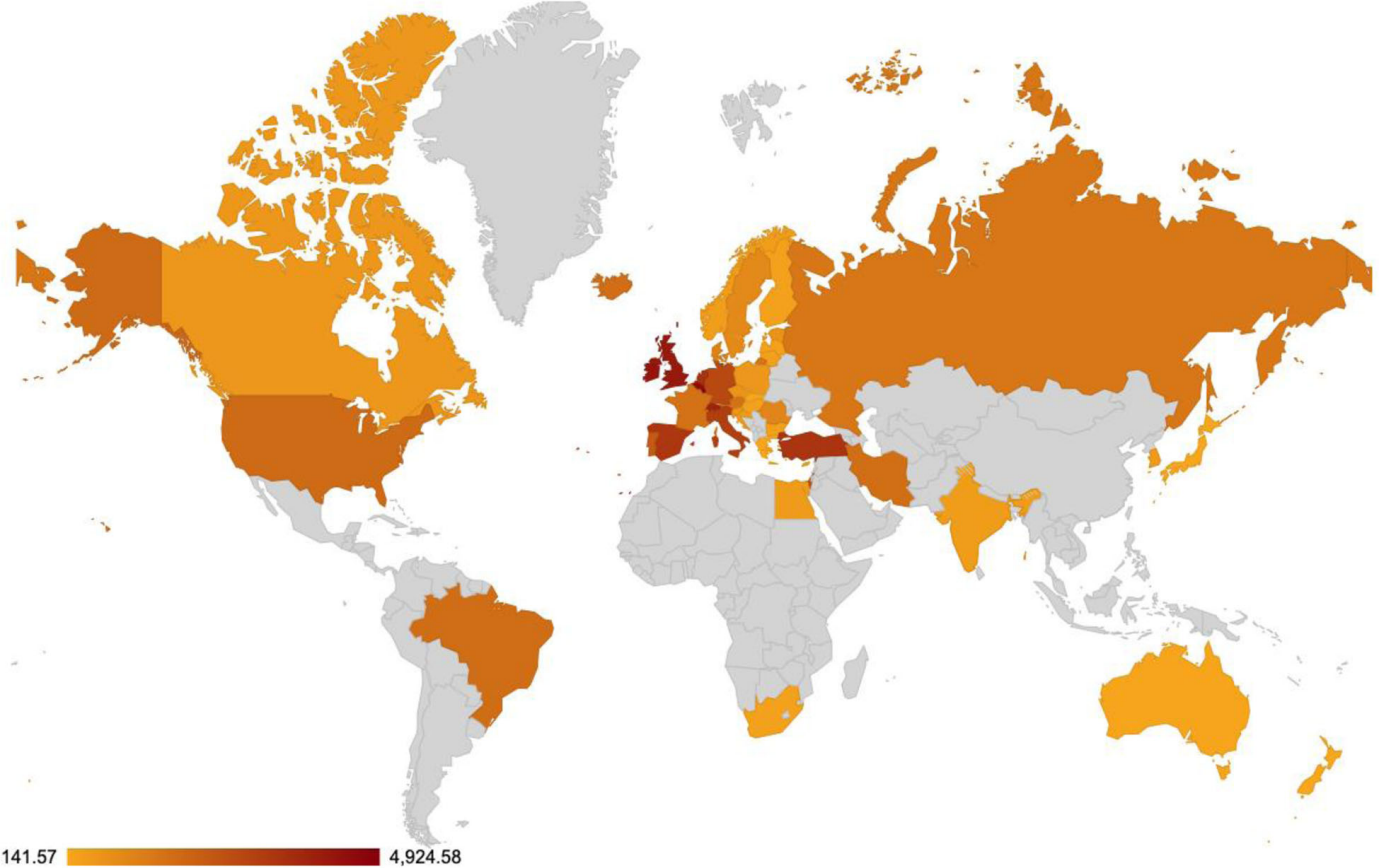
World map with clusters [Chart developed using Google GeoChart (https://developers.google.com/chart/interactive/docs/gallery/geochart)]

The first cluster contains only two countries, namely Belgium and Ireland (Fig. 17). Belgium has more than double the population of Ireland with almost double urban population density per square kilometer (see Table 6). The total number of cases in Belgium was also double the cases in Ireland when taken as an absolute number. This is justified by the population of the two countries which is more than double in Belgium compared to Ireland. However, the number of cases per 1000 km^2^ were very close (4839.75 and 4967.96, respectively) and, therefore, they formed the specific cluster. It is interesting to examine the similarity of their NPI measures to understand whether the measures have any impact on the number of cases. For death cases, Belgium has a higher toll than Ireland with more than 792.50 deaths per million compared to 317.91 up to 18th of May, respectively.

**Table 6:** Countries metrics related to COVID-19 on May, 18th

Cluster	1		2				3		4					5		6		7		8			9					10			SC						
Country	Belgium	Ireland	Netherlands	Spain	Turkey	United Kingdom	Iceland	Malta	Austria	France	Germany	Russia	United States of America	Denmark	Romania	Czech Republic	Estonia	Poland	Slovenia	Croatia	Cyprus	Norway	Bulgaria	Finland	Hungary	Latvia	Lithuania	Greece	New Zealand	Slovakia	Italy	Brazil	Iran	Japan	Taiwan	Sweden	Canada
Country Code	**BEL**	**IRL**	**NLD**	**ESP**	**TUR**	**GBR**	**ISL**	**MLT**	**AUT**	**FRA**	**DEU**	**RUS**	**USA**	**DNK**	**ROU**	**CZE**	**EST**	**POL**	**SVN**	**HRV**	**CYP**	**NOR**	**BGR**	**FIN**	**HUN**	**LVA**	**LTU**	**GRC**	**NZL**	**SVK**	**ITA**	**BRA**	**IRN**	**JPN**	**TWN**	**SWE**	**CAN**
Population (millions)	11.422	48.535	17.231	46.724	82.320	66.489	0.354	0.484	8.847	66.987	82.928	144.478	327.167	5.797	19.474	10.626	1.321	37.979	2.067	4.089	1.189	5.314	7.024	5.518	9.769	1.927	2.790	10.728	4.886	5.447	60.431	209.469	81.800	126.529	23.780	10.183	37.059
Surface Area km^2^ (×1000)	30.5	69.8	41.5	506.0	783.6	242.5	103.0	0.3	83.9	551.5	357.6	17,098	9833.5	42.9	238.4	78.9	45.2	312.7	20.3	56.6	9.3	323.8	110.4	336.9	93.0	64.6	65.3	132.0	268.1	49.0	302.1	8515.8	1628.8	377.9	36.2	438.6	9984.7
Urban Land Area 2010 km^2^	12,349	5638	12,803	69,795	44,090	58,699	1024	293	9823	86,463	62,374	187,538	802,054	9295	15,595	12,536	2615	30,501	2509	5303	2280	20,282	6679	20,052	11,793	3279	4611	18,519	8116	9358	73,541	134,981	69,243	108,678	18,299	31,152	126,511
Population Density	374.15	69.51	414.78	92.34	105.06	274.19	3.43	1535.02	105.48	121.46	231.91	8.45	33.27	135.03	81.69	134.72	29.21	121.46	101.98	72.26	128.56	16.41	63.64	16.38	105.01	29.84	42.73	81.3	18.22	111.08	200.06	24.6	50.22	334.8	657.05	23.22	3.71
ICUs (per 100,000 people)	15.9	6.5	6.4	9.7	32.85	6.6	9.1	20.68	21.8	11.6	43.18	8.3	30.29	6.7	21.4	11.6	14.6	6.9	6.4	14.7	11.4	8	12.2	6.1	13.8	9.7	15.5	6	3.58	9.2	8.43	21.06	4.6	7.3	28.5	5.8	9.5
Date of first case	04-Feb	01-Mar	28-Feb	01-Feb	12-Mar	31-Jan	29-Feb	08-Mar	26-Feb	25-Jan	28-Jan	01-Feb	21-Jan	27-Feb	27-Feb	02-Mar	28-Feb	04-Mar	05-Mar	26-Feb	10-Mar	27-Feb	08-Mar	30-Jan	05-Mar	03-Mar	28-Feb	27-Feb	28-Feb	07-Mar	31-Jan	26-Feb	20-Feb	15-Jan	21-Jan	01-Feb	26-Jan
Date of first death	12-Mar	12-Mar	07-Mar	05-Mar	19-Mar	07-Mar	20-Mar	09-Apr	13-Mar	15-Feb	10-Mar	27-Mar	01-Mar	16-Mar	23-Mar	23-Mar	26-Mar	13-Mar	18-Mar	25-Mar	25-Mar	13-Mar	12-Mar	22-Mar	16-Mar	04-Apr	21-Mar	12-Mar	29-Mar	07-Apr	23-Feb	18-Mar	20-Feb	13-Feb	17-Feb	12-Mar	10-Mar
Total number of cases	55,280	24,112	43,995	231,635	149,435	243,695	1802	553	16,154	142,411	174,697	281,752	1,486,757	10,927	16,871	8475	1774	18,529	1466	2226	916	8197	2235	6347	3535	1008	1541	2834	1149	1494	225,435	241,080	120,198	16,305	440	30,143	76,991
Total number of deaths	9052	1543	5680	27,709	4140	34,636	10	6	629	28,108	7935	2631	89,562	547	1097	298	63	925	104	95	17	232	110	298	462	19	56	163	21	28	31,908	16,118	6988	749	7	3679	5782
Cases per Million	4840	4968	2553	4958	1815	3665	5097	1144	1826	2126	2107	1950	4544	1885	866	798	1343	488	709	544	770	1542	318	1150	362	523	552	264	235	274	3730	1151	1469	129	19	2.960.08	2078
Deaths per Million	792.5	317.91	329.64	593.04	50.29	520.93	28.28	12.41	71.1	419.6	95.69	18.21	273.75	94.35	56.33	28.05	47.7	24.36	50.31	23.23	14.29	43.66	15.66	54	47.29	9.86	20.08	15.19	4.3	5.14	528	76.95	85.43	5.92	0.29	361.28	156.02
Urban Population Density per km^2^	906.43	543.77	1231.33	537.71	1402.98	944.66	323.98	1559.54	525.07	623.24	1027.88	573.43	335.53	548.07	674.3	625.47	347.97	747.81	449.38	439.16	348.42	215.51	788.86	234.96	591.05	400.42	409.45	457.96	520.9	312.71	578.82	1343.41	884.81	1066.64	1013.65	285.8	238.48
Cases per Urban 1000 km^2^	4476	4276	3436	3319	3389	4152	1760	1885	1645	1647	2801	1502	1854	1176	1082	676	678	607	584	420	402	404	335	317	300	307	334	153	142	160	3065	1786	1736	150	24	968	609
Deaths per Urban 1000 km^2^	733	273.66	443.65	397.01	93.9	590.06	9.77	20.45	64.04	325.09	127.22	14.03	111.67	58.85	70.34	23.77	24.09	30.33	41.45	17.92	7.45	11.44	16.47	14.86	39.18	5.8	12.15	8.8	2.59	2.99	433.88	119.41	100.92	6.89	0.38	118.1	45.7

**Fig. 18:**
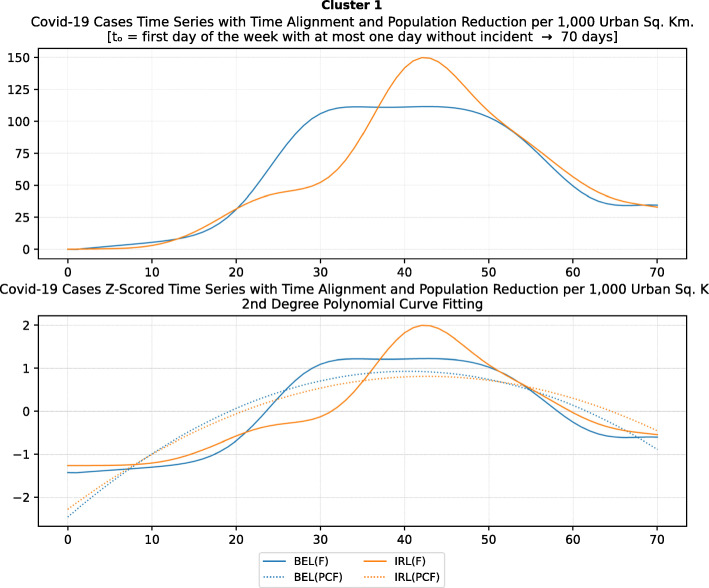
The trend of the cases from the day of the first reported case for the countries of the first cluster

AS shown in Fig. 18, both countries had the same trend in the evolution of their cases starting from the day of the first adjusted reported case up to 71 days after. From the diagram, it can also be deduced that after the first 45 to 50 days, the number of cases started to decline, and thus, the evolution of the cases is very similar. Examining the NPI measures, it seems that both countries waited four to five weeks to start applying social distancing and other measures.

**Fig. 19:**
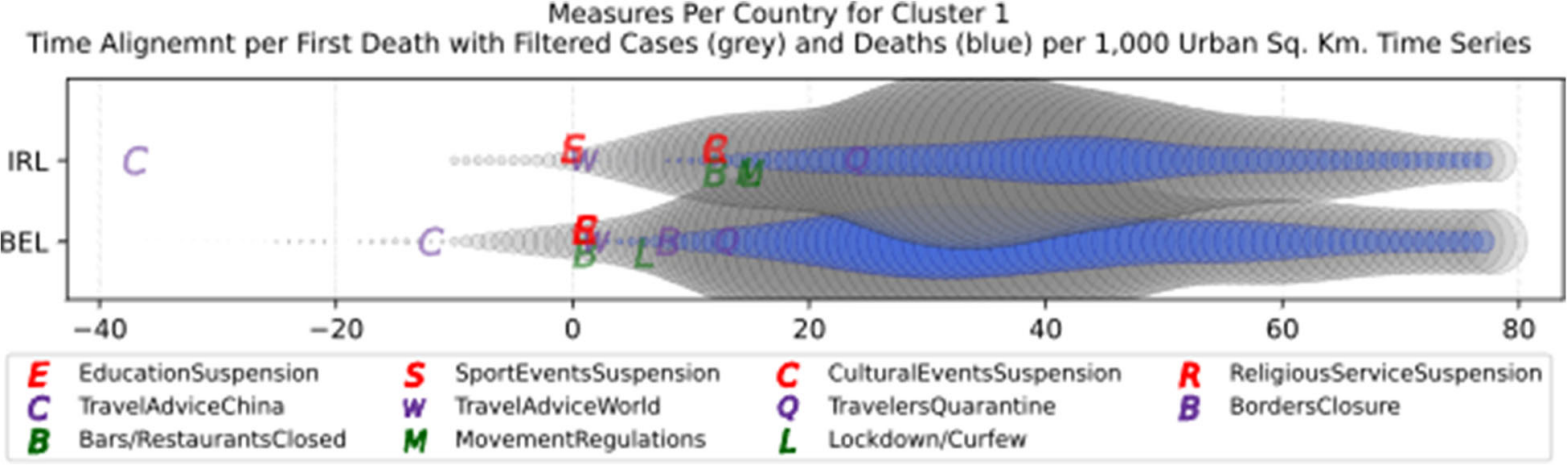
Measures first day aligned by the date of the first death in each country

Obviously, Belgium took several measures very late, and the government did not apply any restriction on movement (Table 5). This can be explained by the fact that Brussels is the Headquarter of the European Commission which requires officials traveling back and forth from all member countries as it was early mentioned. Travelers from European countries with high contamination rates, such Italy and Spain, obviously brought the virus to Belgium which was then seriously affected since no self-isolation and social distancing regulations were imposed. On the other hand, Ireland delayed taking the NPI measures 3 weeks while the government closed schools and suspended sports events a week after its first reported case. However, as it was found later, children appear to be asymptomatic to the virus [68], and thus the impact of the specific measure do not seem to contribute to the elimination of the cases. Ireland also never closed the borders with Northern Ireland and thus people from United Kingdom were free to travel back and forth from Ireland. Since United Kingdom was not able to control its number of cases, it seems that the open border with Ireland affected both countries and led to a very high number of cases per 1000 km^2^ for Ireland. Another interesting observation, comparing the dates when the different NPI measures were taken as reported in Fig. 19 is that both countries took most of the measures close to the date of the first reported death or several weeks after.

### Cluster 2: Netherlands, Spain, Turkey and United Kingdom

The second cluster contains Netherlands, Spain, Turkey, and United Kingdom (Fig. 17). The population of the countries is between 17 million and 82 million, and their population density is rather high especially for Netherlands which has the smallest population and the largest population density of 414.78 as reported Table 6. Concerning the density in urban areas per square kilometer, Turkey and Netherlands lead with 1402.98 and 1231.33 persons, respectively, followed by UK with 944.66 persons and Spain with 537.71 persons. The number of cases per 1000 urban area square kilometers were 3436.31 for Netherlands, 3318.81 for Spain, 3389.31 for Turkey and 4151.62 for United Kingdom. An interesting observation is that the number of deaths per million have been reported by the countries as 329.64, 593.04, 50.29 and 520.93, respectively. However, a discussion has been raised regarding the very low number of deaths reported by Turkey. This is considered by practitioners rather low compared to the numbers reported by other countries in the same cluster or in general [69].

**Fig. 20:**
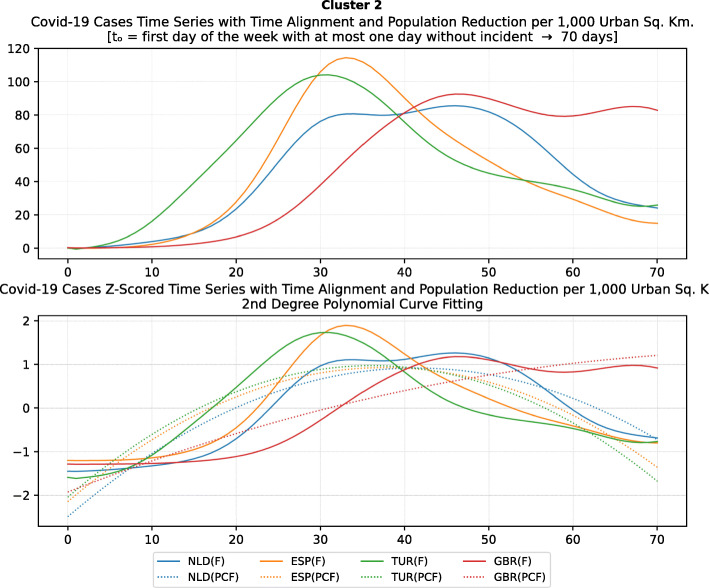
The trend of the cases from the day of the first reported case for the countries of the second cluster

AS depicted in Fig. 20, it seems that all the four countries in this cluster have turned down the curve of the cases after 70 days from the first reported case except for United Kingdom which is still in an uptrend, and thus they can be compared based on the number of persons contaminated per 1000 km^2^.

**Fig. 21:**
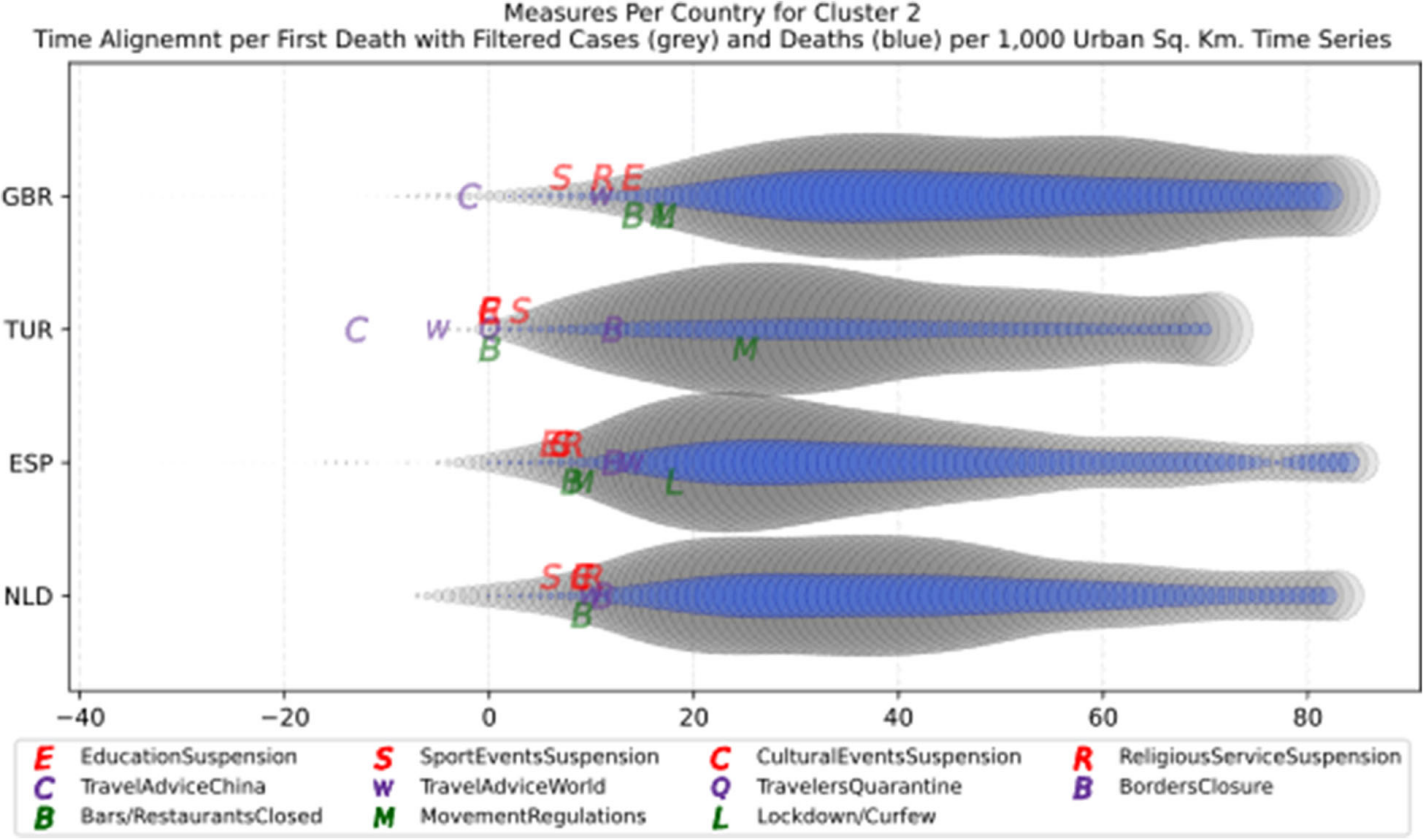
Measures first day aligned by the date of the first death in each country

Studying the NPIs measures that were taken by the four countries in this cluster (Table 5), we observe that Spain and UK waited more than five weeks from the first reported case to take any measure to contain COVID-19 transmission. Netherlands reacted earlier than the other two countries and they took some measures two to 3 weeks after the first reported case. However, as mentioned earlier Netherlands did not take all the measures suggested by WHO, and there was no lockdown or any restriction on the movement of the citizens. In addition, Spain, UK, and Netherlands did not impose a compulsory quarantine on incoming travelers. Thus, comparing these three countries, we can deduce that implementing some NPI measures and skipping others can lead to the same number of contamination cases as taking the NPI measures very late. It is also clear that the restriction on movement greatly affected the number of cases: (1) positively when decided on time, or (2) negatively when delayed or not applied at all. The fourth country in this cluster, Turkey, seems to have taken most of the measures on time and during the first week of the appearance of the first COVID-19 case in Turkey. The government also decided to impose restrictions on the movement of the citizens 4 weeks after the first reported case. That seems to have positively affected the number of cases per 1000 km^2^. Moreover, the restrictions applied in Turkey were not extended to cover the whole population as in most of the other countries worldwide. Instead, they referred only to senior citizens over the age of 65 and to young people below the age of 20 [70]. Another interesting difference in the way that measures were applied in Turkey compared to other countries is that the restrictions on movement were taking effect during weekends and official/religious holidays [71] for 48 to 96 h period only. This decision seems to have been taken to protect the Turkish economy since the restrictions on movement did not affect weekdays, and people were able to go out to work [72]. As it is obvious from the number of cases per 1000 km^2^ in Turkey, the specific strategy was not clearly better than the strategy of the other countries in the cluster which either delayed the restrictions by 6 weeks or did not take any restrictions on movement. Another interesting observation for this cluster is that all the countries took the measures after the first death by COVID-19 was reported; this is obvious in Fig. 21 which seems to have direct effect on the cumulative high number of cases reported.

### Cluster 3: Iceland and Malta

**Fig. 22:**
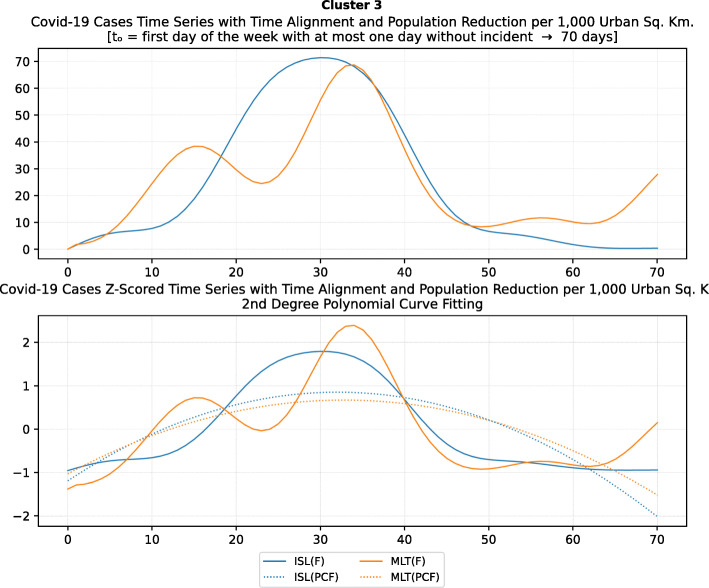
The trend of the cases from the day of the first reported case for the countries of the third cluster

An interesting case is the third cluster in which two islandic countries appear together, namely Iceland and Malta (Fig. 17). Even though they are far away, and they have very different population density (3.43 and 1535.02, respectively) and urban population density per square kilometer (323.98 and 1559.54, respectively) as reported in Table 6, both countries had a very close number of cases per 1000 km^2^ (1760.05 and 1885.18, respectively). While Iceland had a total number of cases of 5096.53 per million by the 18th of May, Malta had only 1143.67 cases per million. In both countries, the number of deaths per million is less than 30, and thus both are considered successful in handling the pandemic. This may be attributed to several speculations which need to be checked and validated, e.g., younger population, healthier people, self-respect to the applied NPI measures, and furthermore their healthcare systems might be more effective and ready compared to other countries. It is noteworthy to mention that Iceland selected to perform a considerable number of tests to its population, and thus may have reported more cases than other countries that had adopted a different policy [73]. As shown in Fig. 22, despite the slightly different shapes of time series, the trends are practically identical.

**Fig. 23:**
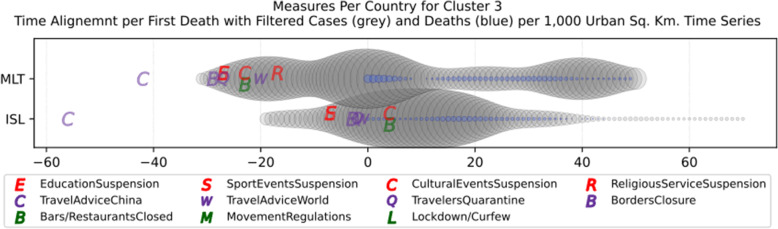
Measures first day aligned by the date of the first death in each country

Concerning the NPI measures reflected in Table 5, both countries appear to have taken all the measures early with significant similarity that both countries did not impose restrictions on movement and did not apply a total lockdown for the whole country. Regarding the implemented measures, both countries suspended educational institutions and sports events in a week or two from the date when the first case was reported. The rest of the measures were implemented the following week, but no later than 3 weeks from the first reported case. It is interesting that both countries took most of the measures before the first reported death or in the same week when the first death case was reported. The decision not to apply a strict lockdown can be justified by the nature of both countries since Iceland and Malta are relatively small islands with population less than half a million. Being islands enabled both countries to better control their borders since they had only to monitor the airports and the seaports. Based on the reports published in the press [73, 74] both countries screened incoming travelers early, and thus it was easier to apply quarantine for those who tested positive. Finally, both countries issued a travel advice against traveling to China very early before the appearance of the first case in each country (Fig. 23), however, it is interesting that Iceland took all the other measures after Malta.

### Cluster 4: Austria, France, Germany, Russia and United States of America

The fourth cluster contains Austria, France, Germany, Russia, and United States (Fig. 17). Three of the countries in this cluster have a unique feature. United States is the largest in terms of population, while Russia has the largest surface area, and Germany has the highest population density per square kilometer (Table 6). The first reported case in France, Germany and the United States was in the last week of January 2020, in Russia on the 1st of February, while in Austria the first case was reported on the 26th of February 2020. The number of cases per million persons is 1825.92, 2125.94, 2106.61, 1950.14 and 4544.33, respectively, with the United States far more ahead compared to the rest at that point of time. This can be attributed to the NPI measures they enforced, or they did not take, or the number of tests they did. All the other countries in this cluster have very similar number of cases per million. Examining the density of the population per square kilometer, Austria has 525.07 persons, France has 623.24, Germany has the largest number, with 1027.88, Russia has 573.43 and the United States has the smallest number, namely 335.53. Since this is the performance indicator used for the clustering, we will discuss how the NPI measures may affect the number of cases per 1000 km^2^.

**Fig. 24:**
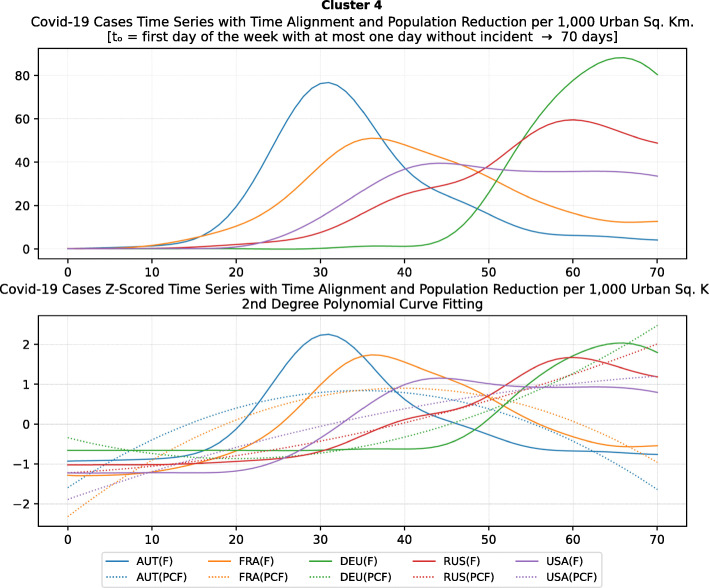
The trend of the cases from the day of the first reported case for the countries of the fourth cluster

According to Fig. 24, the trends show countries in different phases of the pandemic since Austria and France seem to have controlled the disease, Russia and USA appear to have an uptrend while Germany is significantly up. However, Germany for the first two thirds of this period seems to have a very low constant rate of cases that suddenly exploded, and it is expected to eventually stabilize very fast. This may be attributed to the testing strategy that the country adopted.

Based on Table 5, the number of cases per 1000 square meters ranges from 1644.56 for Austria to 2800.79 for Germany. Checking Table 5 for the dates when each measure was taken, it seems that apart from Austria which took the NPI measures after 3 weeks, each other country in this cluster took more or less the same measures 6–7 weeks after its first reported case. Moreover, United States did not suspend cultural or religious events and they did not impose any movement regulation at a national level. At a local level, whether state or city, the respective NPI measures may have been taken depending on the local government.

Another interesting observation is that Germany did not issue a travel advice to China; this may have led to the increased number of total cases per 1000 km^2^, 1000 more than the United States, the second ranking in this cluster. It is also noteworthy that in this cluster three out of five countries did not enforce 14 days quarantine for their travelers arriving from abroad. For the United States, this is not however the case since they decided to stop the flights with most of the countries around the world. However, this measure was taken 59 days after the first reported case.

**Fig. 25:**
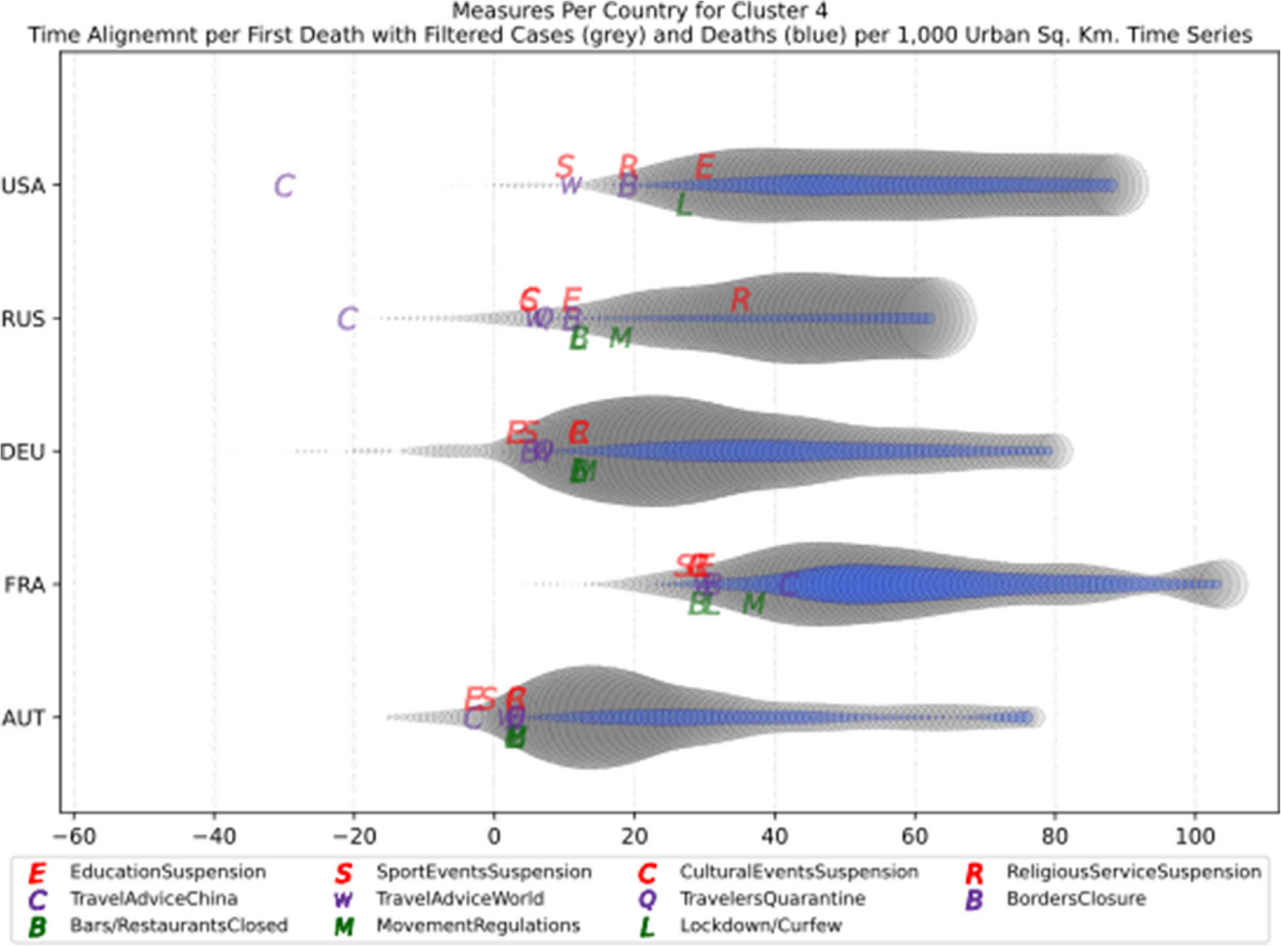
Measures first day aligned by the date of the first death in each country

Some useful findings for Austria are that the first case appeared on 26th of February, almost a month after all the other countries in the cluster. As shown in Fig. 25, Austria enabled all the NPI measures right after its first reported death from COVID-19, while all the other countries have enabled the measures at least 1 week after the first reported death case. Even though the measures were taken relatively early in Austria, an explanation of the increased number of cases per 1000 km^2^ could be linked to the fact that the period of the outbreak January–March is associated with a high holiday season for skiing in the Alps and the common borders with Northern Italy where the first most serious outbreak in Europe occurred. This can be illustrated by the case of the ski resort Ischgl in Alps [75] where Norway declared that 491 returning Norwegian travelers were infected from the virus when they stay at Ischgl.

### Cluster 5: Romania and Denmark

**Fig. 26:**
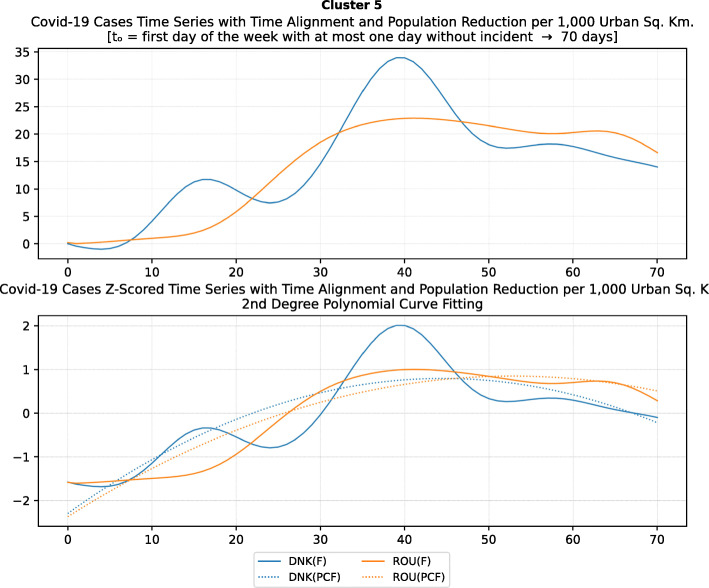
The trend of the cases from the day of the first reported case for the countries of the fifth cluster

Denmark and Romania (Fig. 17) form the fifth cluster, having 1175.56 and 1081.84, respectively, reported cases per 1000 km^2^. Denmark is smaller than Romania, however, in terms of population density, Denmark has almost double density compared to Romania. Concerning the urban population density, Romania has higher population density per square kilometer (Table 6). The virus hit both countries on 27 February 2020, and the first death from the virus was reported on 16 March in Denmark and on 23 March in Romania. After 70 days of the first reported cases, both countries appear to have controlled the disease and the curves have started turning down as shown in Fig. 26.

From the curves, it seems that both countries had some fluctuations in the number of the cases. This can be attributed to the way each country tested the population, and thus both countries are comparable. Comparing cases per million, Denmark appears to have more cases, however, this may again be attributed to the number of tests each country did during the specific period.

**Fig. 27:**
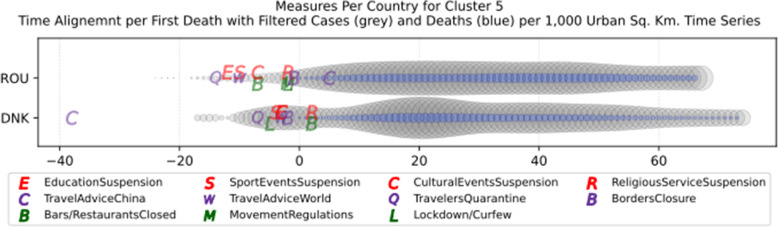
Measures first day aligned by the date of the first death in each country

Concerning the NPI measures applied by both countries, we can see from Table 5 that both countries took all the measures except for the restriction on movement in Denmark. The measures in both countries were taken after the second week, mainly after the first reported case. It is noteworthy that all the measures were taken in both countries before the first reported death case or close to this date (Fig. 27). The slight larger number of cases per 1000 km^2^ for Denmark can be attributed to this measure even though Danish people as Nordic people usually respect the personal space of others, and thus the government of Denmark may have thought that the specific measure is not necessary. However, since all the other measures were the same and were taken almost during the same period after the first reported case in both countries, it seems that the difference between the cases per 1000 km^2^ (1175.56 in Denmark and 1081.84 in Romania) may have been caused by the decision of the Danish government not to apply any restriction on the movement of the citizens. These cultural aspects may be substituted by carefully imposing rules and respecting them. Some of the new centers of COVID-19 may benefit from this experience.

### Cluster 6: Czech Republic and Estonia

**Fig. 28:**
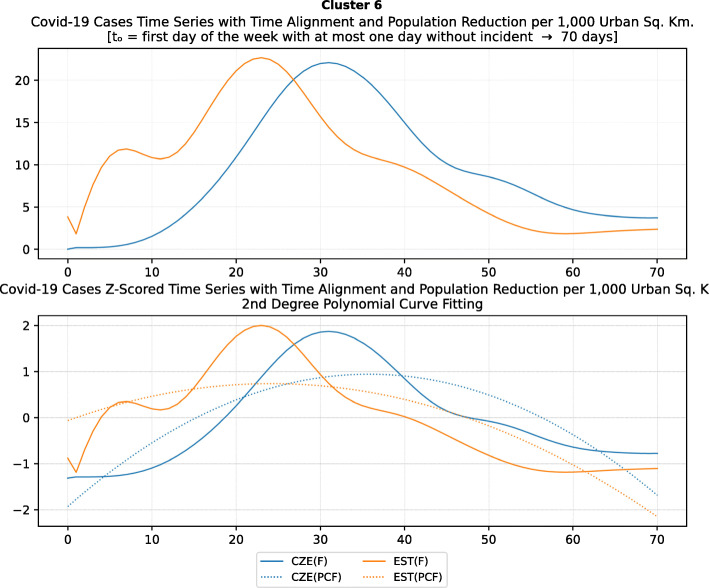
The trend of the cases from the day of the first reported case for the countries of the sixth cluster

The sixth cluster includes just two countries: Czech Republic and Estonia (Fig. 17), with Czech Republic having almost double the surface area of Estonia, but 8 times more population. COVID-19 was firstly reported at the end of February 2020 in Czech Republic and in early March 2020 in Estonia (Table 6). The number of cases per million in Czech Republic is approximately half the number of cases in Estonia. However, in terms of cases per 1000 km^2^, the two countries are very close (676.05 and 678.48, respectively). Both countries managed to turn the curve down after 70 days from the first reported case of the virus as it can be seen in the trend diagram shown in Fig. 28.

**Fig. 29:**
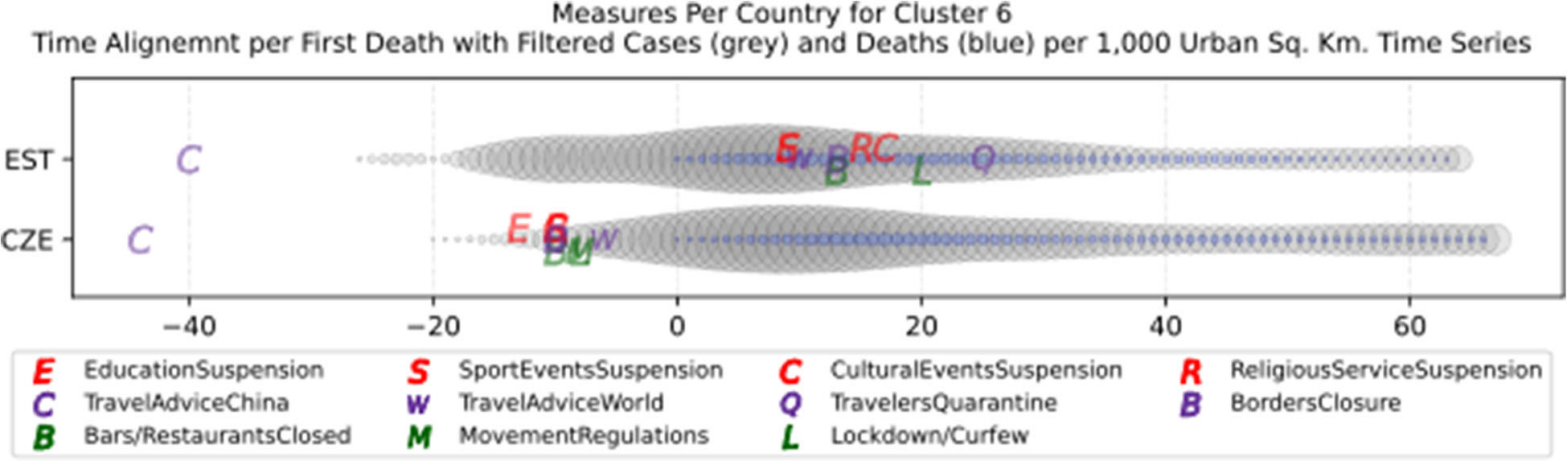
Measures first day aligned by the date of the first death in each country

Analyzing the NPI measures applied, Czech Republic took the measures in the second week after the first reported case (Table 5), while Estonia took the measures a little bit later in the context of the third week which may explain why the number of cases per 1000 km^2^ a little bit is higher than that of the Czech Republic. Another reason that may be attributed towards this little difference may be that the Estonian government did not impose strict restrictions on movement. Another interesting observation is that Estonia took all the measures after the first reported death associated with COVID-19 (Fig. 29). However, this seems not to have a serious effect on the number of deaths per 1000 km^2^. A factor that may have contributed to the comparatively low number of deaths in Estonia is that the population density per 1000 km^2^ is half that of Czech Republic. Regardless of the gap in this measure, it seems that issuing very early travel advice against traveling to China in association with the other NPI measures taken led to controlling the spread of the disease efficiently.

### Cluster 7: Poland and Slovenia

**Fig. 30:**
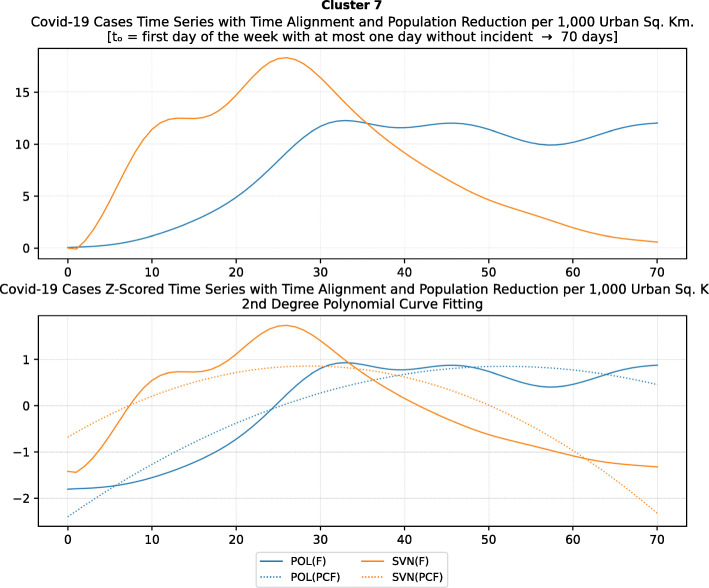
The trend of the cases from the day of the first reported case for the countries of the seventh cluster

The seventh cluster includes Poland and Slovenia (Fig. 17), two European countries with similar population density of 121.46 and 101.98 people per square kilometer, respectively (Table 6). Poland, however, is larger in terms of population and surface area. The urban population density per square kilometer is almost double in Poland with 747.81 people, while it is 449.38 in Slovenia. The first case was reported in both countries in early March 2020 with 1 day difference. The number of cases per million is lower in Poland compared to Slovenia; this may be attributed to the large difference in their population size. Concerning the number of cases per 1000 km^2^ that clustered them together, Poland has 607.48 cases while Slovenia has 584.26. As shown in Fig. 30 both countries have managed to control the evolution of the cases with Slovenia being more successful than Poland by practicably eliminating the spread of the disease after 70 days while Poland has just managed to stabilize the rate of the disease.

Table 5 presents the NPI measures applied by the two countries. It can be easily observed that both countries reacted very fast, and they took most of the measures 1 week after their first reported COVID-19 case. Interestingly, Slovenia did not apply a general lockdown; instead, it imposed restrictions on movements after 2 weeks from the first case. Another significant point is that Slovenia did not took any measure to quarantine incoming travelers, even though the borders were closed when the rest of the measures were taken. The slight delay of 3 days for Slovenia to take the measures in comparison to Poland and the delay in issuing travel advice against traveling abroad may justify the higher number of cases per million that Slovenia had (709.11) compared to Poland (487.88).

**Fig. 31:**
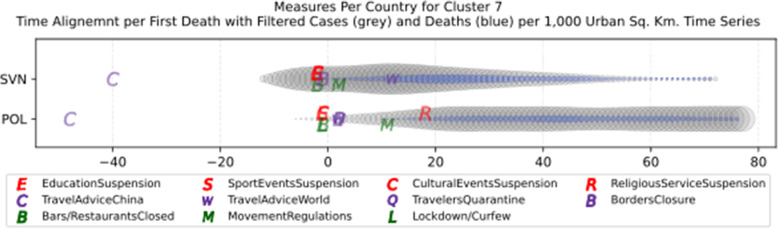
Measures first day aligned by the date of the first death in each country

From Fig. 31, we can observe that Poland delayed 4 weeks to suspend the religious service compared to Slovenia. This seems not to affect drastically the number of cases per 1000 km^2^. As many other countries did, both Poland and Slovenia took most of the measures just before the first death attributed to COVID-19, and thus it seems that decision affected the overall evolution of the transmission of the virus in both countries.

### Cluster 8: Norway, Cyprus and Croatia

**Fig. 32:**
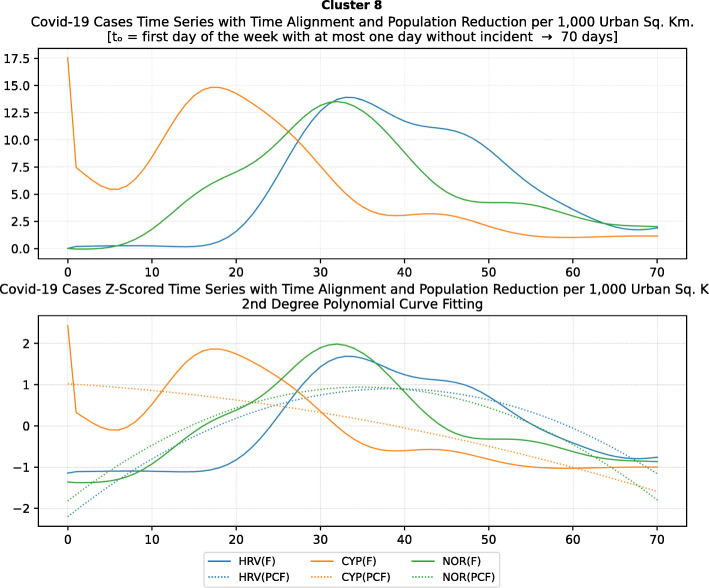
The trend of the cases from the day of the first reported case for the countries of the eight cluster

The three countries Norway, Cyprus and Croatia end up in the same cluster (Fig. 17). They all have population less than 5 million. Their urban population density is 439.16, 348.42 and 215.51, respectively (Table 6). Norway has the lowest population density because it has the largest surface area of 323,772 km^2^. In terms of cases per million, Norway had many more cases than the other two countries. Checking the number of cases per 1000 km^2^, the cases range from 401 to 420, and thus they ended up in the same cluster. Other characteristics of these three countries are the relatively small population of 5 million for Norway, 4 million for Croatia and a little above 1 million for Cyprus. The trend diagram (Fig. 32) shows that after 70 days from the first reported case these three countries have managed to handle the crisis and turned down the curve with relatively the same trend.

**Fig. 33:**
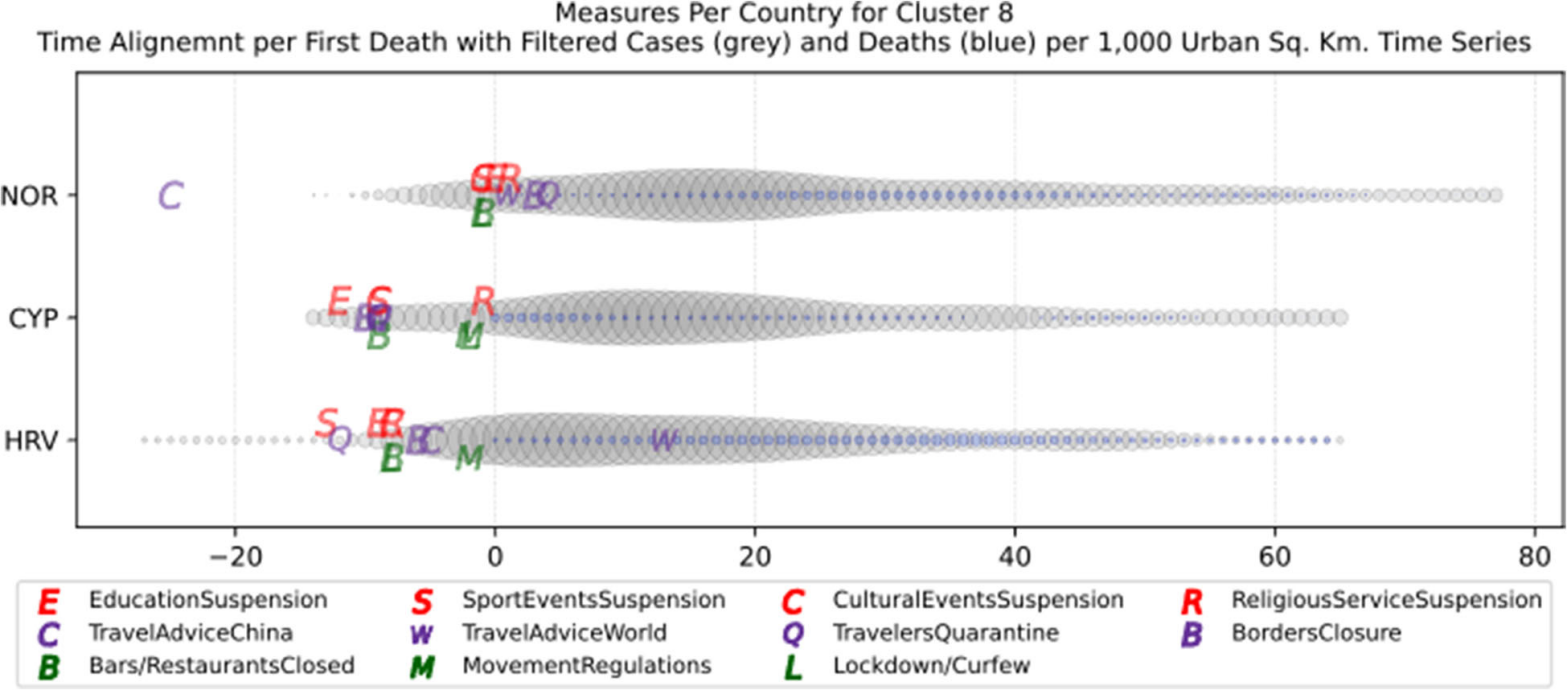
Measures first day aligned by the date of the first death in each country

Looking at the NPI measures table (Table 5), Norway and Croatia took most of the measures between the second and the third weeks of the first reported case which was almost on the same date at the end of February 2020. The first reported case in Cyprus was 2 weeks after the other countries, and thus the first measures taken by the local government were 1 week after the first case. However, Cyprus did not suspend religious services and did not initially impose restrictions on the movement of people. These were suspended later after the second week, just before the first death was reported in the country (Fig. 33). Another difference between the three countries is that in Norway the government did not impose strict regulations on citizens’ movement which may be attributed to the fact that Norwegian citizens were already used to social distancing before the pandemic as a cultural behavior that each one should respect the personal space of others [76]. In summary, all the countries in this cluster took the same measures two to three weeks after the first reported case and just before the first reported death. This has led them to have a very close number of reported cases per 1000 km^2^. Finally, the three countries ended up having the same rules respected by their citizens either by mentality like in Norway or by explicitly imposing them as it is the case with the other two countries. Indeed, Norway may form a good example for other countries to spread within their population the same style of living and respect of social means.

### Cluster 9: Bulgaria, Finland, Hungary, Latvia and Lithuania.

**Fig. 34:**
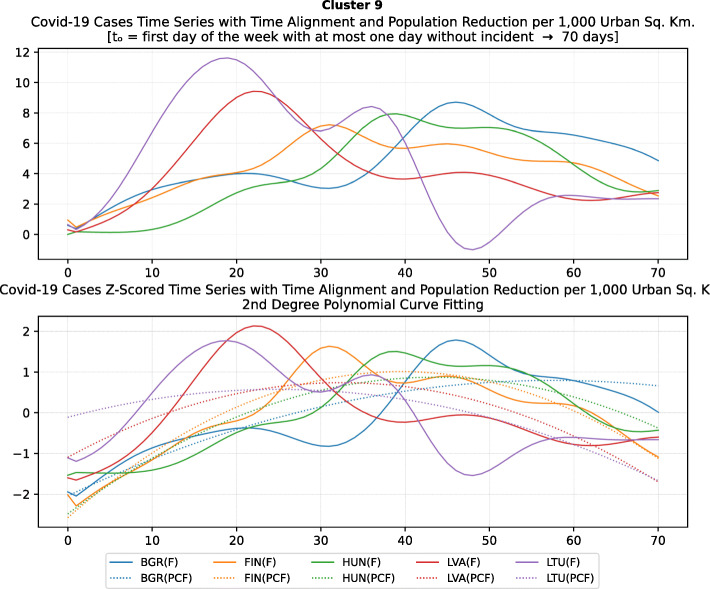
The trend of the cases from the day of the first reported case for the countries of the ninth cluster

The ninth cluster consists of five relatively small European countries: Bulgaria, Finland, Hungary, Latvia, and Lithuania (Fig. 17) with population ranging from 2 million to 10 million (Table 6). Finland is the largest country in terms of surface area, while it is very sparsely populated. Latvia and Lithuania, on the other hand, have the same size in terms of surface area and very similar urban population density per square kilometer (400.42 and 409.45, respectively). It is noteworthy that both countries had almost the same number of cases per million (523.22 and 552.42, respectively). In terms of cases per 1000 km^2^ of urban area, which is the criterion for clustering these countries together, Bulgaria and Lithuania have the most cases (334), and Hungary has the least (299) cases. From the trend diagram (Fig. 34), we can see that Finland, Latvia and Lithuania have managed to turn the curve down after 70 days from the first reported case with comparable trends in the number of cases, while Hungary has just started to turn down the curve and Bulgaria has just started stabilizing the curve.

Looking at the NPI measures, someone may say that countries in this cluster did not take the same measures even though they have very close number of cases per 1000 km^2^. However, if we analyze Table 5, we can observe that Latvia and Lithuania took very few measures such as closing the educational system, which was implemented 2 weeks after the first reported case. Both issued travel advice against traveling to China very early, then against traveling in general, and finally they closed their borders 2 weeks after the first reported case. Hungary took more measures than Latvia and Lithuania, however, the religious services were not suspended.

**Fig. 35:**
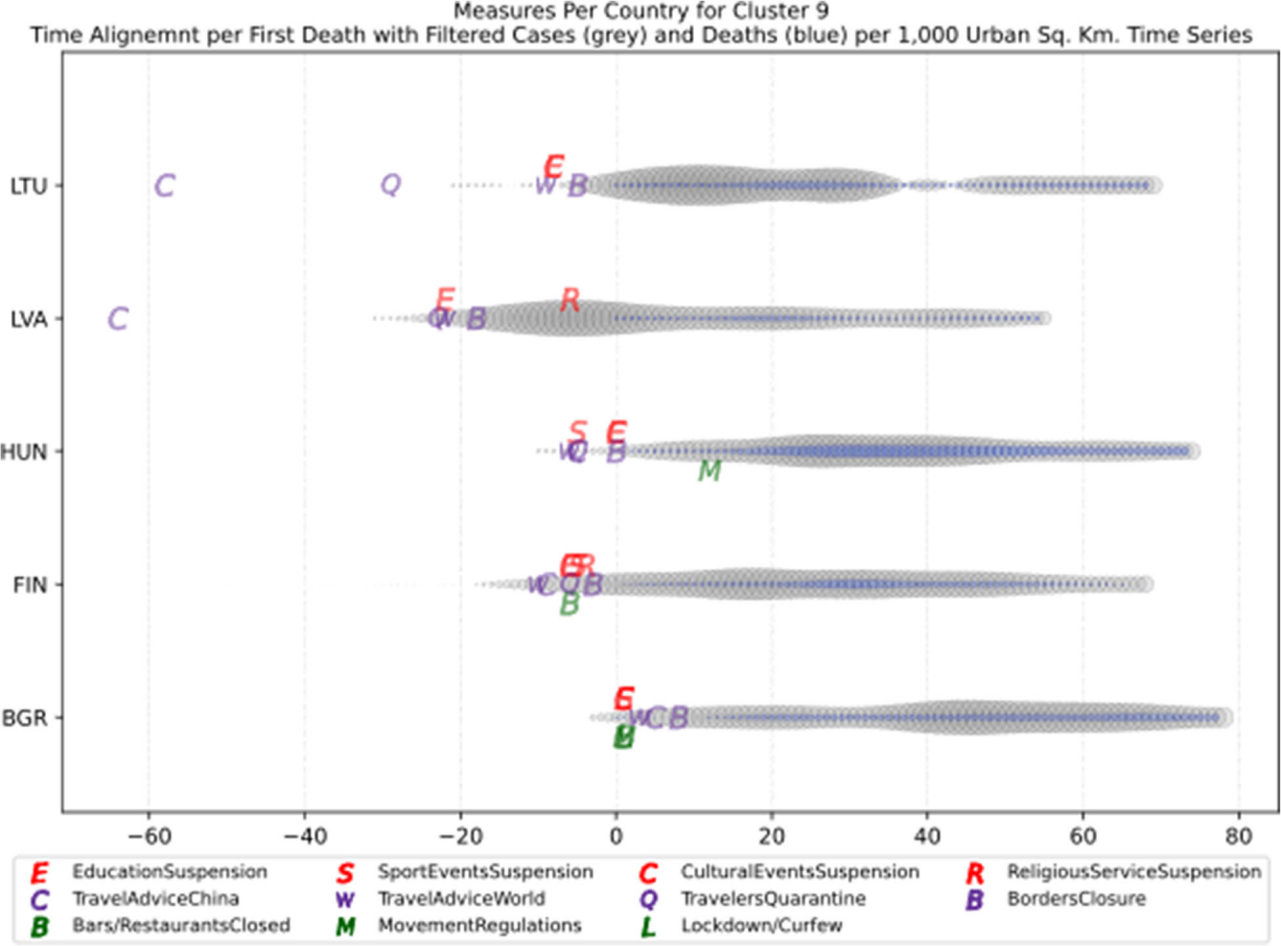
Measures first day aligned by the date of the first death in each country

Finland even though applied all the containment measures proposed by WHO, this was very late, namely six weeks after the first reported case. The government did not put the whole country in a lockdown and people were able to move without restrictions. However, Finland had a very low constant number of cases for weeks (like Germany) and practically took the measures soon before the first death cases. Finally, Bulgaria took all the proposed measures very early except the suspension of religious services, and incoming travelers were not initially put in quarantine upon arrival. However, the country closed the borders early just 2 weeks after the first reported cases. Having Bulgaria in this cluster with the other countries which took the measures late or which did not put in effect all the measures taken by Bulgaria may be attributed to the higher urban population density per square kilometer which is almost double than that of the other countries, except Hungary which has almost 80% of the Bulgarian urban population density per square kilometer. In terms of deaths, as reported in Fig. 25 and Fig. 35, the absolute numbers are low between 10 and 54 per million, while all the countries reacted fast and took all the measures before the first reported death except Bulgaria which took the measures 1 week after the first reported death. This may be another reason why Bulgaria clustered with this specific group of countries.

### Cluster 10: Greece, New Zealand and Slovakia

The tenth cluster contains Greece, New Zealand and Slovakia (Fig. 17). The population of the countries ranges from almost 11 million in Greece to 5 million in New Zealand and 5 and a half million in Slovakia. In terms of surface area, New Zealand is double the size of Greece, while Slovakia is the smallest with approximately 49 thousand square kilometers (Table 6). The population density of Slovakia is the largest followed by Greece, while New Zealand has the lowest population count.

**Fig. 36:**
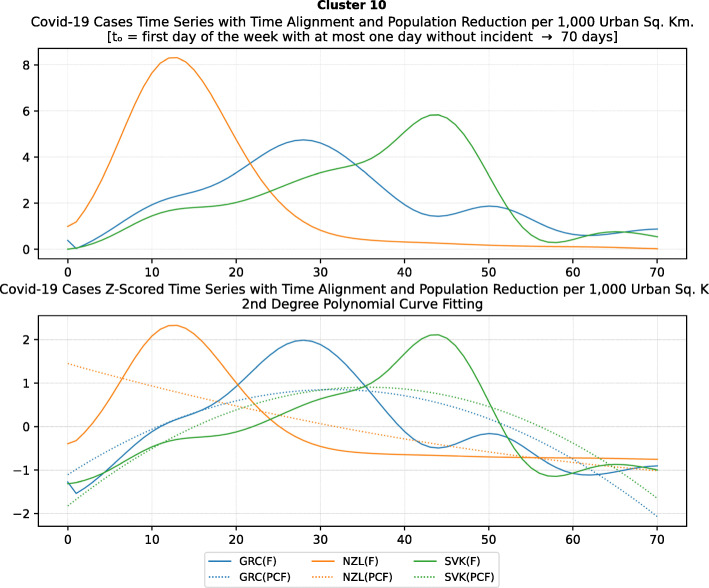
The trend of the cases from the day of the first reported case for the countries of the tenth cluster

Examining the population density over 1000 km^2^ in urban areas, New Zealand is the first with 520.90 people per 1000 km^2^, followed by Greece with 457.96, and lastly Slovakia with 312.71 persons per square kilometers. The first reported case of COVID-19 in Greece and New Zealand was at the end of February, while in Slovakia the first case appeared in the first week of March 2020 As shown in Fig. 36, the trend of the pandemic for the three countries has turned down after 70 days, and thus they managed to control the spread of the disease very successfully. The number of cases per 1000 km^2^ is 153.03 for Greece, 141.57 for New Zealand and 159.65 for Slovakia.

**Fig. 37:**
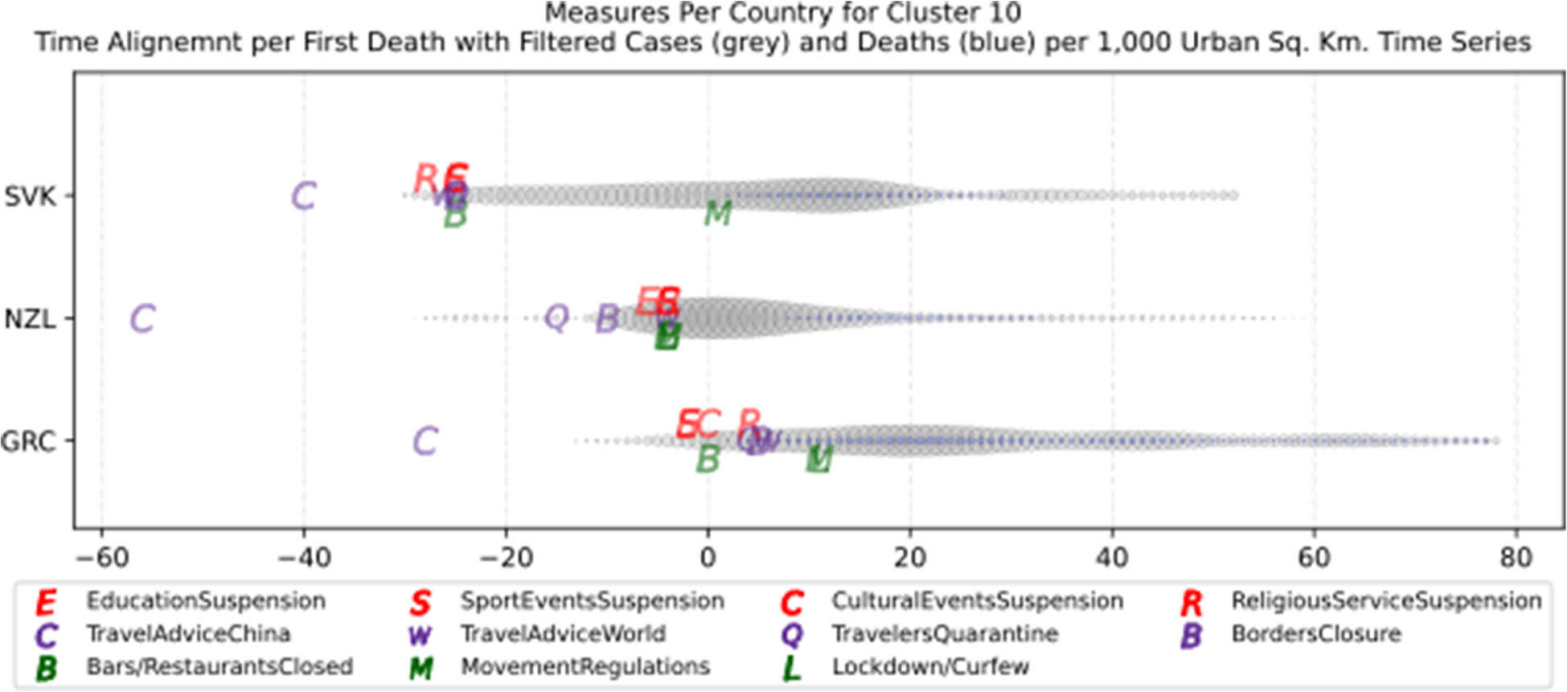
Measures first day aligned by the date of the first death in each country

The three countries in this cluster implemented all the NPI measures suggested by WHO (Table 5). Greece and Slovakia took the measures relatively early in the first or second week after the appearance of the first reported case of COVID-19, while New Zealand took the measures in the fourth week after the first case. As shown in Fig. 37, the NPI measures were decided in all the three countries before the first death except for Greece which imposed movement restrictions and a national lockdown a week after the first death. It is noteworthy, that movement restrictions and national lockdown were decided in all the three countries between the third and fourth weeks from the first reported COVID-19 case which seems to be the most decisive factor for these three countries to cluster together with relatively low number of cases per 1000 km^2^. Another observation that may have played a role in the low number of cases per 1000 km^2^ for all the countries is the travel advice against traveling to China which has been issued very early. The actual lockdown in Greece may be seen as substituted by having New Zeeland an isolated island. That is, both countries had their population isolated from the outside world and the culture of keeping distance by default in New Zeeland is again substituted by the lockdown in Greece, leading to similar trends in these countries.

### Interesting special cases: countries with distinct trend

From Table 5 and Fig. 7, it can be seen that there are more countries in the dataset that present some interesting cases since they were either hit by the disease hardly, such as Italy, or they did not take any measures such as Brazil or Sweden, or followed a hybrid approach such as Iran and Canada, or they managed to have very few cases even though they took only very precise measures such as Taiwan and Japan (Fig. 17). All these countries appear as special cases, failing to cluster with other countries in the clustering of cases per 1000 km^2^, but as we mentioned above, they are worth to study their approach in containing the pandemic.

Italy is one of the first countries in Europe where the first COVID-19 cases appeared very early in late January (Table 6). It is a very large country with population more than 60 million and population density 200.06. It was expected that controlling the spread of the disease would not be easy since it is the country with the highest aging population globally, and it was hit severely by the virus. In terms of cases per million, Italy has an extremely high number of cases (3730.44), and it has 3065.44 cases per 1000 urban square kilometers in 70 days.

**Fig. 38:**
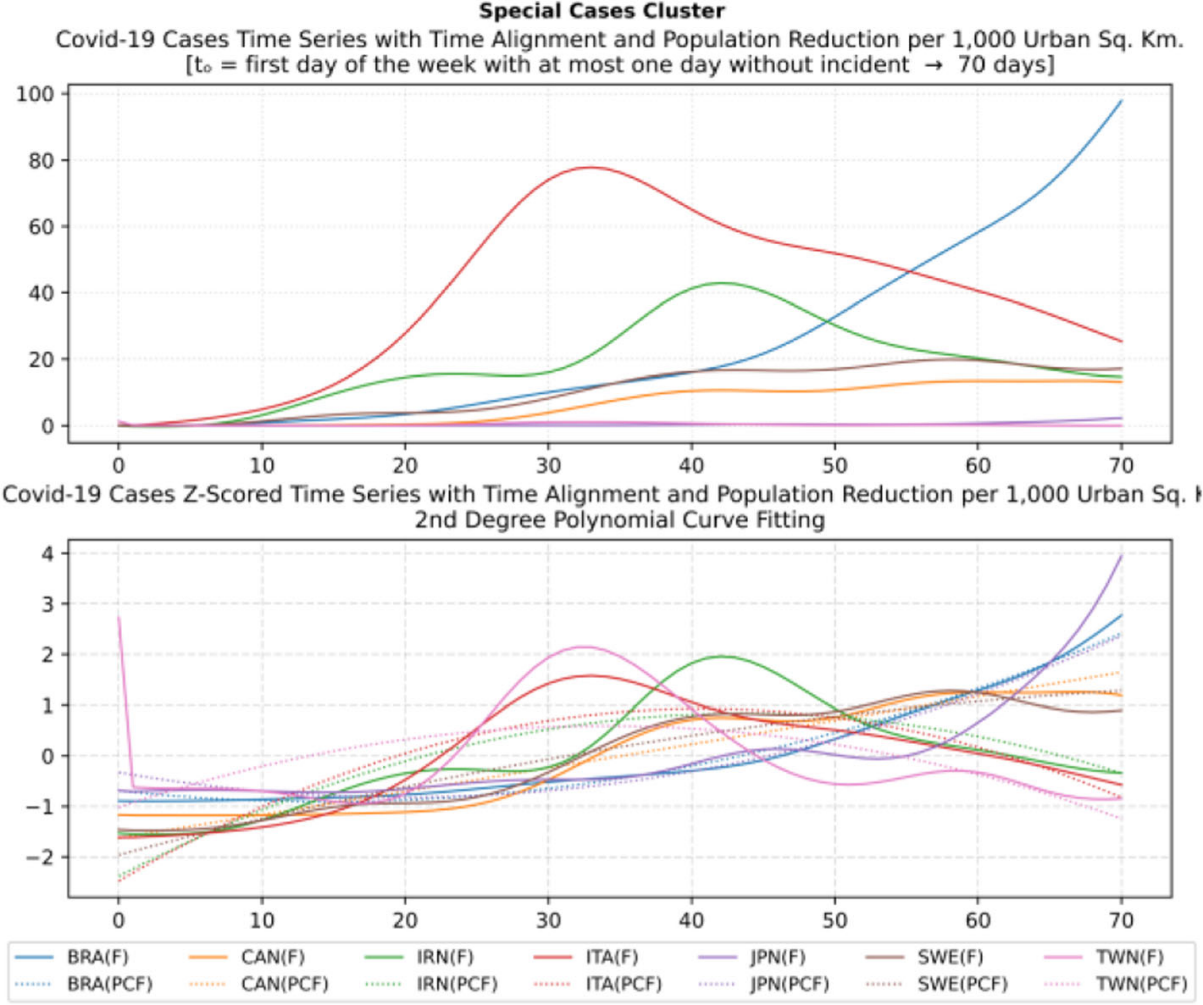
The trend of the cases from the day of the first reported case for the countries of the special cases cluster

**Fig. 39:**
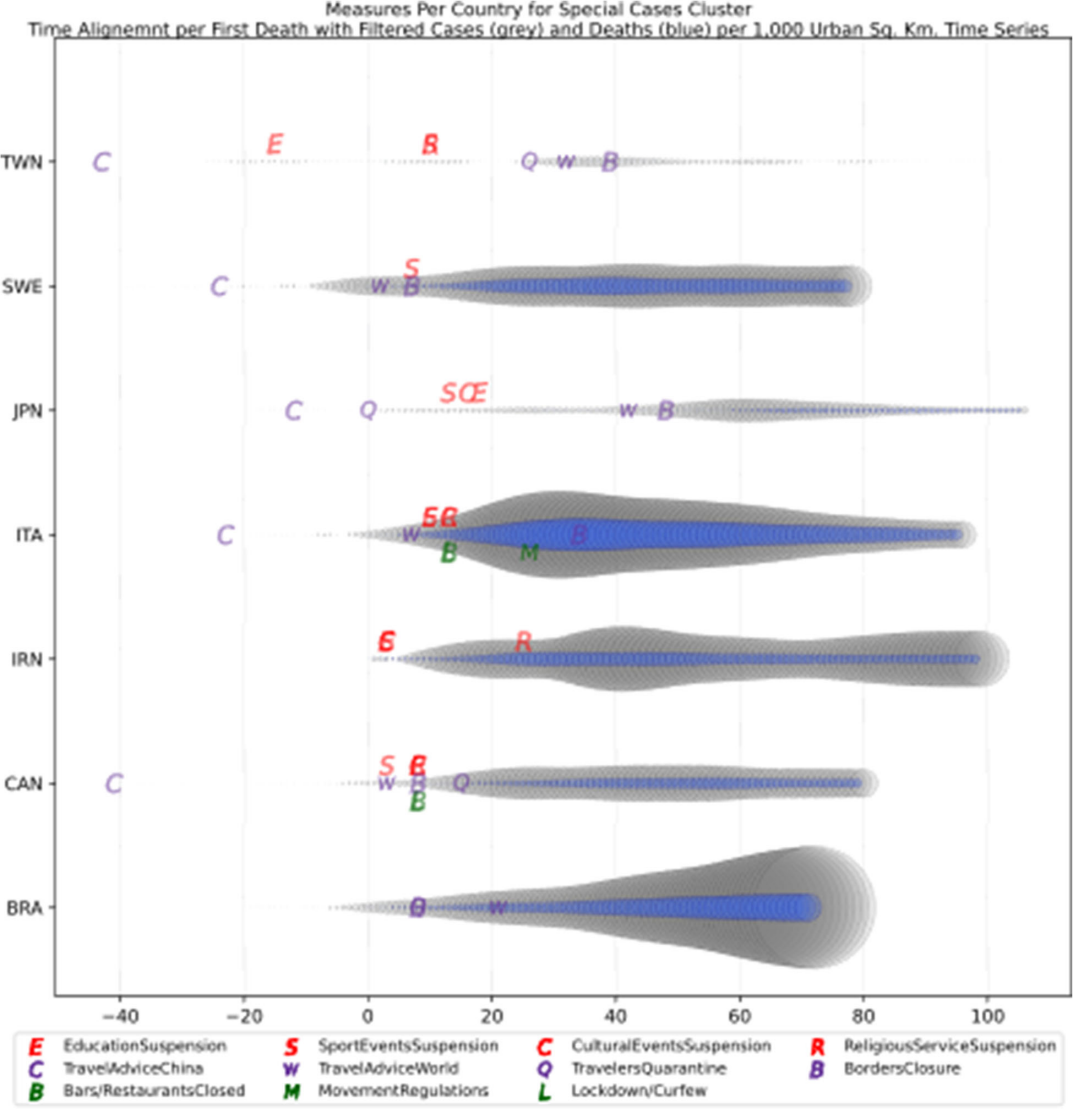
Measures first day aligned by the date of the first death in each country

Table 5 shows that Italy took more or less all the NPI measures suggested by WHO, but too late, namely five or 6 weeks after the first confirmed case. The delay in response to the pandemic seems to have greatly affected the number of cases and pushed Italy to pay a high toll in deaths with 528.00 deaths per million (Table 6). As one of the first European countries that was hit by the virus, it is justified for not taking measures immediately since there was no experience in handling the pandemic and the messages from the Asian countries which had already confirmed cases were not clear at the beginning. Figure 38 shows that after 70 days of the outbreak, Italy has managed to start stabilizing the curve. From Fig. 39 shows that Italy took the measures at least 2 weeks after the first reported death, and thus this seems to be the most crucial factor that affected the evolution of the disease with thousands of deaths over the initial weeks.

The next two countries that seem to have relevant statistics are Brazil and Iran (Fig. 17). Both countries diagnosed the first confirmed cases in late February (Table 5), however in Iran the first reported death is in the same day with the first confirmed case, while in Brazil the first death came several weeks later after mid-March. Regarding Iran, it seems that the health system of the country was not able to early detect the virus and as a result crucial time was lost. Checking the evolution of the pandemic in both countries, we can see that the cases per million for Brazil is 1150.91 while for Iran 1469.41. Brazil is five times larger than Iran, but more densely populated with 1343.41 people per urban area square kilometer, while Iran has 884.81 people per urban area square kilometer. The number of cases per 1000 km^2^ is almost the same 1786.02 in Brazil and 1735.89 in Iran. Checking the trend of the disease, Fig. 38 shows that Brazil is in the initial phase of the pandemic, while Iran has started to manage stabilizing the curve for some time before it turned again up during the days when this paper was written, may be Iran is getting the second wave. Interestingly, it appears in Table 5 that both countries did not take the majority of the NPI measures suggested by WHO. Brazil only closed the borders with the neighboring countries and issued travel advice against traveling to China, and later to the rest of the world after three and 4 weeks, respectively. On the other hand, Iran suspended sports and cultural events and closed schools. The main problem with the evolution of the pandemic in Iran seems to be the late diagnosis of the first confirmed case which means that the authorities did not have the time to react to, and hence they did not take the necessary measures to contain the virus. Brazil, on the other hand, not taking any significant social distancing measure appears to be in the beginning of a very steep uptrend in the cases contaminated with the virus, and it does not seem to have the ability to control the disease soon (Figs. 38 and 39).

Japan and Taiwan are two very interesting outliers because both have the lowest cases per 1000 km^2^ of urban area as shown in Fig. 7, which depicts the progress of the time series of the first 70 days since the first reported cases. Both countries are islandic with high population density of 334.80 in Japan and 657.05 in Taiwan. Since Taiwan does not appear as a sovereign country in The World Bank [55], the urban land area was calculated based on the Department of Land Administration, M.O.I. land utilization [77]. Another common characteristic of both countries is that they are closest neighbors of China, and thus very close to the source of the pandemic. However, both countries had been affected by the previous epidemics of the SARS virus, and thus they both had the experience to handle similar diseases. In Japan the number of cases per million is 128.86, while it is 18.50 in Taiwan, which is one of the lowest rates worldwide. Similarly, in our criterion Japan has 150.03 case per 1000 urban area square kilometers, while Taiwan is just only 24.05, which is again the lowest worldwide. Both countries, compared to the others, had the first reported case of virus contamination very early in mid-January.

As shown in Fig. 38, Taiwan seems to have turned the curve down and they have contained the disease close to zero cases at the time of writing this paper, while Japan appears to have a steep uptrend for the first 70 days. However, the full time series shown in Fig. 39 describes how Japan very quickly managed to control the outbreak and stabilized the spread of the virus to low levels. From Table 5, we can deduce that both countries took partial measures from those suggested by WHO. However, as mentioned before, because of their islandic nature, the training of the population to live with an epidemic such as SARS, and the population mentality which promotes the use of personal protective measure in their everyday life, the social distancing and personal hygiene measures as devised by WHO were already applied without the governments enforcing them. Another interesting observation is that both countries issued travel advice against traveling to China as the source of the pandemic, and both suspended sports events and closed educational institutions. Finally, they applied screening measures for incoming travelers at their points of entry.

The last two outliers are Sweden and Canada (Fig. 17). Both countries have some common characteristics such as very low population density of 23.22 and 3.71 persons per square kilometer, respectively. The urban population density is also similar at 285.80 and 238.48 persons per square kilometer of the urban area, respectively. In both countries, the first confirmed case appeared approximately at the same time, end of January with a gap of 6 days. The number of cases per million in Sweden is approximately 50% more with 2.960.08 cases in comparison to Canada with 2077.53 cases. Similarly, the number of cases in urban area per 1000 km^2^ for Sweden is 967.60 cases; this is 60% more than that of Canada which is 608.57 cases. After 70 days of the outbreak, they seem to have stabilized the curve at a high rate (Fig. 38), and as shown in Fig. 39, they continued at this high rate when this paper was written in June 2020. Concerning the NPI measures taken, as reported in Table 5, the only measures taken by Sweden were to suspend the sports events and to issue travel advice against traveling. These measures were also taken very late, 6 weeks or more after the appearance of the first case in the country. Canada on the contrary, applied all measures suggested by WHO six or more weeks after the first confirmed case of COVID-19 except for issuing a travel advice against traveling to China only 3 days after the first confirmed case.

### Further observations regarding mortality in European areas

**Fig. 40:**
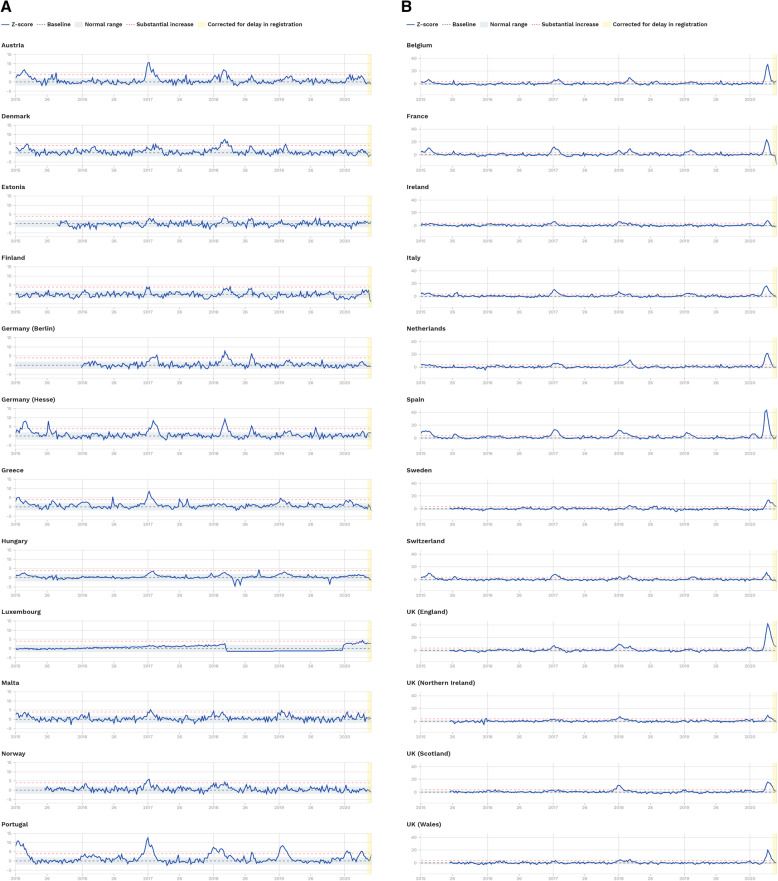
Z-Scored Mortality Rate by country [78]

In addition to our analysis and results, we can observe from Euromomo [78] some very interesting facts and figures regarding mortality in Europe for the first 20 weeks of 2020 compared to the previous 4 years (2016–2019). Euromomo uses data from some European countries and regions such as, Austria, Belgium, Denmark, Estonia, Finland, France, Germany (Berlin), Germany (Hesse), Greece, Hungary, Ireland, Italy, Luxemburg, Malta, Netherlands, Norway, Portugal, Spain, Sweden, Switzerland, UK (England), UK (Northern Ireland), UK (Scotland) and UK (Wales). It is interesting to observe as shown in Fig. 40 two groups of countries based on their Z-Scored mortality rates. As shown in Fig. 40(a), the first group of countries managed to maintain a normal or close to normal mortality rate, while the second group shown in Fig. 40 (b) has a substantially increased mortality rate. It is important to mention that for the first group the y-axis in Fig. 40 (a) is scaled between 0 and 15, while for the second group the y-axis in Fig. 40 (b) is scaled between 0 and 40. Cross-checking the countries in Fig. 40 with Table 5, it can be observed that countries which took the NPI measures reflected normal mortality rates in Fig. 40 (a), while countries which delayed taking the NPI measures or did not take any NPI measures reported have excessive peaks in mortality rates as shown in Fig. 40 (b).

## Discussion

### Strengths

Our motivation to analyze the confirmed and death cases attributed to SARS-CoV-2 in association with the NPI measures adopted by the countries worldwide is based on: (a) the diversity of the policies, (b) the lack of determination that showed some countries rapidly implemented the NPI measures, (c) the decision of some other countries to not take any measure at all in order to protect their social and economic life, and (d) the profound and severe results of the infection on the population. Utilizing data-mining techniques and machine learning methods, we have been able to provide as much as possible sound and justified answers to the research questions stated in the problem definition section.

More precisely, according to our findings, the response to the first two questions is that it seems there are two major trends about the NPI measures implementation regarding countries which took the measures early and strictly implemented them and countries which either took the measures with delay and without determination or they didn’t implement any policy at all. For example, there are countries such Greece, Israel, Luxembourg, and Slovenia (Table 5, Fig. 5.a) which took measures relatively fast and managed to have a low death rate per 1 million population, while other countries such as Belgium, Canada, France, Italy, Spain, United Kingdom, and USA (Table 5, Fig. 5.c, 5.d and 5.e) which took measures very late, or Brazil and Sweden (Table 5) which didn’t take any NPI measures have not managed to control the death rates. These findings can be further validated from the Euromomo data as presented in Fig. 40.

Based on the clusters presented in the analysis of the NPI measures, the third and fourth questions have been addressed by several useful observations. Specifically, regarding each category of the NPI measures, we report the following findings:

- Curfew/restrictions on movement seems to have directly and positively affected slowing down the spread of the disease and decreasing the number of cases since countries which did not take any such measure or took it very late suffered by having more confirmed cases than countries which took the specific measure early (see cluster 10).
- Travel advice to the country of origin of the pandemic seems important but needs to be taken in combination with other measures such as quarantine of the incoming travelers, etc. (see cluster 3 and 10). For example, Greece issued a travel advice and quarantined every traveler not only from China but also from neighboring countries like Italy which was the first European country suffered heavily from the spread of COVID-19.
- Quarantine and screening of the incoming travelers also appear to be very important for controlling the disease since countries that took the specific measure had lower rate of contamination by the virus (see clusters 6, 8 and 10).
- Taking measures before the first death seems to be very effective in controlling the transmission of the virus. In other words, it has been observed that limiting the transmission of the virus becomes feasible when the measures are taken during the first 2 weeks after the first case, and the overall number of cases per 1000 km^2^ becomes less compared to other countries that took measures after the first 2 weeks (see Table 5).
- Sport and cultural events suspension appear to have contributed towards the reduction of the number of cases since that helped the citizens to keep social distances easier. Especially in Europe where has significant sport leagues which involve multiple countries, such as Football Champions League and Basketball Euroleague, the temporal suspension of the leagues helped to control the spread among countries. However, some countries did not stop the football matches of the local leagues and that led to a high spread of the disease like in Italy.
- The suspension of schools or universities possibly affected positively the control of the transmission of the virus in combination with other NPIs measures taken in parallel. Logically, it should have direct effect because schools and universities are locations for daily gathering of large masses. Indeed, schools have always been identified as a main source for the spread of the seasonal flu. Further, because of the winter break in most central and north European countries, schools were already closed during the outbreak. Therefore, there was no clear evidence if the specific measure played an important role in controlling the spread of COVID-19.
- Similarly, locking malls, restaurants, coffee shops, etc., and restricting the number of persons inside a grocery store has reflected positively on the number of cases by indirectly imposing social distancing. However, this has direct negative effect on the economy and hence most countries tried to avoid this measure or managed to shorten its period. Indeed, this measure has shifted the trend from the tradition of in person presence to get service in these locations to online alternative by electronic shopping and ordering of commodities and services.
- Working from home has become a trend and both organizations and employees started to investigate ways of adapting this though the reaction to working from home ranges within the same community. However, in the case of COVID-19, working from home became an inescapable necessity because of the imposed circumstances such as lockdown and suspension of various businesses. The specific measure was delayed since it was relied to the governments for the public sector and employers of large companies for the private sector. Therefore, it is difficult to determine its implication in the case of COVID-19. Moreover, tracking down when this measure was applied by the individual organizations in a country is not practical and cannot be studied in correlation with the other NPI measures.

Regarding the final research question about the positive and negative lessons which could be learnt and possible restructure of the policies, we report some findings that could help decision makers to better plan national and global response in case of a second wave of COVID-19 or another highly contagious pandemic in the forthcoming months or years, especially if there will be no associated pharmaceutical measures in place. More specifically:

- It seems that delaying the NPI measures after a specific time interval from the first death has practically little effect on leveling down the spread of the disease rather than stabilizing the daily cases rate at high level (see Sweden, Canada, United Kingdom and United States). The specific cluster of countries can also be seen in Fig. 2 (a) and Figs. 21, 25 and 39, showing the percentage similarity of cases time series. Countries which did not take the measures had the same evolution of cases compared to countries that delayed taking the measures by approximately more than 3 weeks after the first death. The evolution of the number of deaths could depend on several factors such as ICUs availability, medical personnel, aging population, health and environmental factors, etc.
- The success of the NPIs measures also depends on the way each government monitored their application. Countries with more loose policing related to the measures may be less effective in controlling the transmission of the disease, and furthermore the cultural mentality could also affect the success of the measures because of self-discipline and social responsibility.

### Limitations

Even though our findings shed light for the first time on the relation of NPIs to the contagiousness and mortality of COVID-19, there are limitations that should be taken into consideration. The most significant limitation is related to the available data. Real time data stream, reporting confirmed cases and deaths from different sources and organizations, such as ECDC should accumulate, process, and provide to the public, could lead to temporary inconsistencies because of different reporting methodologies by individual countries. Therefore, thorough data cleansing and verification is important.

Moreover, some countries reported the NPI measures regarding COVID-19 in their domestic language only, making the acquisition of information difficult. In addition, some countries adopted different approaches in the implementation of the NPI measures, making the categorization hard. For example, there were countries which banned social gatherings using different threshold of people, e.g., above 5, 10, 50, 100, 500, etc. Additionally, some countries partially implemented several measures, such as closing bars, restaurants, etc. and allowing small open space places, like coffee-shops to operate. On top of this, some countries which have different federal organization and governmental systems such as USA and UK, applied the measures at the regional level only, making their categorization more complicated. Another limitation related to the NPI measures is the inconsistency found between having the government announcing the application date of a measure and the official date recorded in the governmental gazettes, which makes it difficult to understand the initial date when the measure was applied.

Regarding the data analytics methodologies used in this study, there are also a couple of limitations. First, the time series of the confirmed and death cases are very short, usually between 60 and 80 data points (days), and highly noisy because of the different types of recording as described above. Therefore, filtering is an important step to smoothen the corresponding curves of the time series under the analysis and to make it easier to detect the trends for the analysis, but it could distort the curves if not applied carefully. Second, machine learning clustering is not deterministic and could provide different results for any slight modification in the dataset length, either expansion or reduction, due to the small length of the time series. Here, it is worth mentioning that after the attempt to open the economy again, the NPI measures have been revoked in a different way by individual countries or restriction measures have been imposed again locally, making any further attempt for general analysis of the effectiveness of the NPI measures practically impossible. For example, in USA, some state governments have applied local lockdowns in some areas because of a rising in the number of infections while others have not.

### Implications

Despite the limitations, our study is the first to provide a comprehensive overview of the association between NPIs and contagiousness and mortality of COVID-19. Furthermore, it provides an extensive list of sources collected for the first time and provided as it is to other researchers that could significantly help them in their studies of NPIs implications by saving their time. Another contribution of the study is the collection and organization of the implemented NPIs in a large number of countries which is very useful for the scientific community. Data from many global and national institutions have been collected and analyzed that provide reliability to the results of our study. Data mining techniques used in our study, such as ARPaD and GPSC algorithms, provide deterministic results and, therefore, the findings are reproducible. Additionally, there is no need for a statistical hypothesis and test which cannot be easily validated due to the limited dataset. The study introduces a novel index, the urban population density, for the precise description of the situation among different countries and a more comprehensive comparison. Finally, a plethora of different visualization techniques could lead to easier interpretation of the findings.

## Conclusions

In this study, we have collected in one dataset, variables, and information that we consider related to the COVID-19 pandemic. The collected data help in understanding the evolution of the disease in relation to the NPI measures suggested by WHO. In this process, we used publicly available datasets from ECDC and other official sources, recording the time series of confirmed and death cases attributed to COVID-19 and profile data of the countries have been fetched from the World Bank and OECD, related to population demographics. We have manually collected and categorized from governmental institutions the preventive measures that several countries took to protect their citizens from the spread of the pandemic. The data collected was then projected to the protective measures suggested by WHO to better understand how the countries adopted the suggested measures and to what extent the specific measures affected the spread of the disease in those countries. Several data analyses were then conducted, such as clustering for confirmed and death cases time series to identify similarities between the evolution of the pandemic and the NPI measures applied. Clustering per 1000 km^2^ of the urban areas was then used to compare the NPI measures implemented by countries which were clustered together. We also examined how these NPI measures have affected the number of cases. A detailed presentation of the profile of each country in the cluster was presented and the basic indicators regarding the cases of COVID-19 were compared for the countries in each cluster. While statistical hypothesis testing was not applied, and, therefore, no statistical model that could be directly reused for other cases has been created, the proposed methodology could be smoothly re-implemented as a data-mining process for similar situations in the future.

Moreover, some very interesting observations were recorded, and together with the detailed dataset we believe that the specific work will be very useful to epidemiologists or decision makers. First, immediate application of the preventive measures such as travel advice against traveling to and from the origin of the pandemic, entry screening and quarantine to all incoming travelers have been proved to be very effective in avoiding the spread locally at the early stages. After the discovery of the first confirmed cases in a country, and, before, the first death, other measures such as social distancing, selective closures, suspension of social activities, etc. can significantly contribute to controlling the outbreak and keep the pandemic at low level that the health care systems can handle. More strict measures such as lockdown is inescapable when the pandemic burst gets out of control. In all cases, when the NPI measures are implemented, the government should thoroughly and constantly monitor them. Decision makers can further analyze the collected data in combination with no-public datasets of hospitalized patients, genetic data, or other data related to factors which could affect the respiratory system, such as smoking, air pollution in urban areas, etc. This may lead them to valuable conclusions regarding the impact of the NPIs measures in the evolution of the COVID-19 pandemic.

## Supplementary Information

**Additional file 1:.** Legends of the Figures

**Additional file 2:.** List of Websites

**Additional file 3: Appendix** - COVID-19 Time Series Analysis Plots

## Data Availability

Data and material used in this study can be obtained freely by the sources listed in “Additional file 2: List of websites”.

## Abbreviations

ARPaD: All repeated pattern detection
CDC: Centers for disease control and prevention
COVID: Corona virus disease
DBSCAN: Distance based spatial clustering of applications with noise
ECDC: European center for disease prevention and control
GPSC: General purpose sequence clustering
ICU: Intensive care unit
LERP-RSA: Longest expected repeated pattern-reduced suffix array
MERS: Middle East respiratory syndrome
M.O.I: Ministry of the interior
N1H1: Hemagglutinin type 1 and neuraminidase type 1
NPI: Non-pharmaceutical intervention
OECD: Organization for economic cooperation and development
PHSM: Public health and social measures
SARS: Severe acute respiratory syndrome
SARS-CoV-2: Severe acute respiratory syndrome-corona virus-2
SCG: Sequence commonality grouping
SCM: Sequence commonality matrix
UNESCO: United Nations educational, scientific and cultural organization
WHO: World health organization
