# Supplementary material for: COVID-19 pandemic spread against countries’ non-pharmaceutical interventions responses: a data-mining driven comparative study
Source: BMC Public Health. 2021 Sep 1;21:1607. doi: 10.1186/s12889-021-11251-4 (PMC8409702; doi:10.1186/s12889-021-11251-4)
Supplement: Supplementary file 1 — Additional file 1:. Legends of the Figures [file 12889_2021_11251_MOESM1_ESM.docx]

**Additional File #1: Legends of the Figures**

Fig. 1: Days Interval Histogram between First Case and Death

Fig. 2: COVID-19 Cases Clustering – Sequence Commonality Grouping (a) and (c)/Matrix (b) and (d) for 80% and 60% similarity

Fig. 3: COVID-19 Cases Original/Z-Scored Time Series with Time Alignment and Population Reduction per 1 Million with Second Degree Polynomial Curve Fitting (a)-(j)

Fig. 4: COVID-19 Deaths Clustering – Sequence Commonality Grouping (a) and (c)/Matrix (b) and (d) for 80% and 60% similarity

Fig. 5: COVID-19 Deaths Original/Z-Scored Time Series with Time Alignment and Population Reduction per 1 Million with Second Degree Polynomial Curve Fitting (a)-(e)

Fig. 6: Cases per 1 Million (Logarithmic Normalization)

Fig. 7: Cases per 1,000 Square Kilometers (Logarithmic Normalization)

Fig. 8: Days Interval Boxplot between Measures Imposed Date and First Case

Fig. 9: Days Interval Boxplot between Measures Imposed Date and First Death

Fig. 10: Educational measures and workplace and public place measures

Fig. 11: Social distancing and closure of restaurants

Fig. 12: Travel measures

Fig. 13: Number of countries per week which enabled education suspension

Fig. 14: Number of countries per week which enabled sports events suspension

Fig. 15: Number of countries per week which enabled religious events suspension

Fig. 16: Number of countries per week which enabled lockdown

Fig. 17: World map with clusters

Fig. 18: The trend of the cases from the day of the first reported case for the countries of the first cluster

Fig. 19: Measures first day aligned by the date of the first death in each country

Fig. 20: The trend of the cases from the day of the first reported case for the countries of the second cluster

Fig. 21: Measures first day aligned by the date of the first death in each country

Fig. 22: The trend of the cases from the day of the first reported case for the countries of the third cluster

Fig. 23: Measures first day aligned by the date of the first death in each country

Fig. 24: The trend of the cases from the day of the first reported case for the countries of the fourth cluster

Fig. 25: Measures first day aligned by the date of the first death in each country

Fig. 26: The trend of the cases from the day of the first reported case for the countries of the fifth cluster

Fig. 27: Measures first day aligned by the date of the first death in each country

Fig. 28: The trend of the cases from the day of the first reported case for the countries of the sixth cluster

Fig. 29: Measures first day aligned by the date of the first death in each country

Fig. 30: The trend of the cases from the day of the first reported case for the countries of the seventh cluster

Fig. 31: Measures first day aligned by the date of the first death in each country

Fig. 32: The trend of the cases from the day of the first reported case for the countries of the eight cluster

Fig. 33: Measures first day aligned by the date of the first death in each country

Fig. 34: The trend of the cases from the day of the first reported case for the countries of the ninth cluster

Fig. 35: Measures first day aligned by the date of the first death in each country

Fig. 36: The trend of the cases from the day of the first reported case for the countries of the tenth cluster

Fig. 37: Measures first day aligned by the date of the first death in each country

Fig. 38: The trend of the cases from the day of the first reported case for the countries of the special cases cluster

Fig. 39: Measures first day aligned by the date of the first death in each country

Fig. 40
