# Supplementary material for: COVID-19 pandemic spread against countries’ non-pharmaceutical interventions responses: a data-mining driven comparative study
Source: BMC Public Health. 2021 Sep 1;21:1607. doi: 10.1186/s12889-021-11251-4 (PMC8409702; doi:10.1186/s12889-021-11251-4)
Supplement: Supplementary file 2 — Additional file 2:. List of Websites [file 12889_2021_11251_MOESM2_ESM.docx]

Z-Scored Mortality Rate by country [75]

**Additional File #2: List of Websites**

|  | **Australia** |
| --- | --- |
| AUS1 | https://www.health.gov.au/resources/publications/australian-health-sector-emergency-response-plan-for-novel-coronavirus-covid-19-short-form |
| AUS2 | https://www.smartraveller.gov.au/news-and-updates/coronavirus-covid-19 |
| AUS3 | https://www.australia.gov.au |
| AUS4 | https://www.health.gov.au/news/health-alerts/novel-coronavirus-2019-ncov-health-alert |
|  | **Austria** |
| AUT1 | https://metropole.at/coronavirus-which-stores-are-open-which-are-closed-in-austria/ |
| AUT2 | https://infothek.bmvit.gv.at/coronavirus-reisehinweise-aktuelle-informationen/ |
| AUT3 | https://www.austria.org/coronavirus |
|  | **Belgium** |
| BEL1 | https://www.euractiv.com/section/coronavirus/news/belgium-enters-lockdown-over-coronavirus-crisis-until-5-april/ |
| BEL2 | https://diplomatie.belgium.be/fr/Newsroom/overview |
|  | **Brazil** |
| BRA1 | https://proinde.com.br/news/coronavirus-preventive-health-measures-in-brazilian-ports-and-airports/ |
| BRA2 | https://brazilian.report/coronavirus-brazil-live-blog/ |
|  | **Bulgaria** |
| BGR1 | https://www.inyourpocket.com/sofia/the-coronavirus-covid-19-in-bulgaria-travel-info_77571f |
| BGR2 | https://home.kpmg/content/dam/kpmg/us/pdf/2020/03/tnf-bulgaria-mar17-2020.pdf |
| BGR3 | https://www.euractiv.com/section/justice-home-affairs/news/bulgaria-bans-entry-to-foreigners-from-almost-all-the-world-over-covid-19/ |
| BGR4 | https://www.schengenvisainfo.com/news/bulgaria-bans-from-entering-citizens-of-13-european-countries/ |
| BGR5 | https://sofiaglobe.com/2020/04/04/bulgarias-coronavirus-crisis-staff-chief-on-easter-2020-do-not-go-to-church/ |
| BGR6 | https://www.mfa.bg/bg/news/24785 |
|  | **Canada** |
| CAN1 | https://www.canada.ca/en/global-affairs/news/2020/03/government-of-canada-advises-canadians-to-avoid-non-essential-travel-abroad.html |
| CAN2 | https://www.canada.ca/en/public-health/services/diseases/2019-novel-coronavirus-infection/canadas-reponse.html |
| CAN3 | https://www.canada.ca/en/public-health/services/publications/diseases-conditions/covid-19-government-canada-research-response.html |
| CAN4 | https://www.justice.gc.ca/eng/csj-sjc/covid.html |
|  | **Croatia** |
| HRV1 | http://www.mvep.hr/hr/mediji/priopcenja/,32781.html |
| HRV2 | https://www.koronavirus.hr/vladine-mjere/101 |
| HRV3 | https://civilna-zastita.gov.hr/vijesti/vijesti/priopcenje-za-medije-stozera-civilne-zastite-republike-hrvatske-od-9-ozujka-2020/2237 |
| HRV4 | https://pravosudje.gov.hr/vijesti/obavijest-strankama-o-nacinu-upucivanja-zahtjeva-upita-i-molbi-ministarstvu-pravosudja/21711 |
|  | **Cyprus** |
| CYP1 | http://www.mfa.gov.cy/mfa/mfa2016.nsf/All/540D4E2656EEF856C225852D002A942F?OpenDocument |
| CYP2 | https://in-cyprus.philenews.com/coronavirus-full-address-of-president-anastasiades-announcing-the-lockdown/ |
| CYP3 | http://www.cylaw.org/KDP/data/2020_1_117.pdf |
| CYP4 | https://www.pio.gov.cy/ανακοινωθέντα-άρθρο.html?id=12403#flat |
|  | **Czech Republic** |
| CZE1 | https://www.mvcr.cz/mvcren/article/state-of-emergency.aspx |
| CZE2 | http://www.msmt.cz/informace-k-vyhlaseni-nouzoveho-stavu-v-cr |
|  | **Denmark** |
| DNK1 | https://um.dk/da/rejse-og-ophold/rejse-til-udlandet/rejsevejledninger/kina/ |
| DNK2 | https://um.dk/da/nyheder-fra-udenrigsministeriet/newsdisplaypage/?newsID=6A907C87-94DF-4F0C-ACC2-59429CBAF5DC |
|  | **Egypt** |
| EGY1 | https://www.garda.com/crisis24/news-alerts/322876/egypt-country-to-close-schools-universities-for-two-weeks-from-march-15-over-covid-19-concerns-update-6 |
| EGY2 | https://www.worldnomads.com/travel-safety/north-africa/egypt/latest-egypt-travel-warnings-alerts |
| EGY3 | https://www.aa.com.tr/en/latest-on-coronavirus-outbreak/egypt-suspends-flights-over-coronavirus-outbreak/1768360 |
|  | **Estonia** |
| EST1 | https://vm.ee/et/uudised/valisministeerium-uuendas-seoses-koroonaviiruse-levikuga-reisisoovitusi |
| EST2 | https://vm.ee/et/uudised/valisministeeriumi-reisiinfo-eriteade |
|  | **Finland** |
| FIN1 | https://um.fi/ajankohtaista/-/asset_publisher/gc654PySnjTX/content/koronavirustilanne-ja-umn-matkustustiedotteet |
| FIN2 | https://um.fi/matkustustiedote/-/c/CN |
|  | **France** |
| FRA1 | https://www.thelocal.fr/20200315/france-coronavirus-lockdownwhats-closed-and-whats-open-in-france-following-the-new-coronavirus-restrictions |
| FRA2 | https://www.thelocal.fr/20200313/the-last-day-when-everything-will-be-normal-all-french-schools-will-close-on-monday-to-fight-the-coronavirus |
| FRA3 | https://www.france24.com/en/20200308-france-bans-gatherings-of-more-than-1-000-people-to-contain-coronavirus |
| FRA4 | https://www.politico.eu/article/emmanuel-macron-eu-external-borders-to-close-from-tuesday/ |
| FRA5 | https://www.catholicnews.com/services/englishnews/2020/churches-begin-canceling-masses-in-effort-to-stem-covid-19-pandemic.cfm |
| FRA6 | https://www.diplomatie.gouv.fr/fr/conseils-aux-voyageurs/conseils-par-pays-destination/chine/ |
|  | **Germany** |
| DEU1 | https://colombo.diplo.de/lk-en/coronavirus/2317268 |
| DEU2 | https://www.bundesgesundheitsministerium.de/presse/pressemitteilungen/2020/1-quartal/gesetzespakete-corona-epidemie.html |
| DEU3 | https://www.bundesregierung.de/breg-de/themen/coronavirus/besprechung-der-bundeskanzlerin-mit-den-regierungschefinnen-und-regierungschefs-der-laender-1733248 |
|  | **Greece** |
| GRC1 | https://www.iq-mag.net/2020/03/event-bans-enforced-in-czech-republic-greece/#.XonZlEXVI1I |
| GRC2 | https://www.euractiv.com/section/health-consumers/short_news/greece-update-covid-19/ |
| GRC3 | https://greece.greekreporter.com/2020/03/22/greece-goes-on-coronavirus-lockdown/ |
| GRC4 | https://twitter.com/GreeceMFA?ref_src=twsrc%5Etfw%7Ctwcamp%5Eembeddedtimeline%7Ctwterm%5Eprofile%3AGreeceMFA&ref_url=https%3A%2F%2Fwww.mfa.gr%2Fepikairotita%2Fenimerosi-syntakton%2F |
| GRC5 | https://www.mfa.gr/china/en/the-embassy/news/13022020-new-announcement-of-the-embassy-of-greece-on-the-coronavirus-cases-in-china.html |
| GRC6 | https://www.e-nomothesia.gr/kat-ygeia/astheneies/praxe-nomothetikou-periekhomenou-tes-20-3-2020.html |
| GRC7 | https://www.civilprotection.gr/el/simantika-themata/kiryxi-prosorinis-epivolis-periorismoy-kat-oikon-karantina-ton-katoikon-ton |
|  | **Hungary** |
| HUN1 | http://abouthungary.hu/news-in-brief/coronavirus-heres-the-latest/ |
| HUN2 | https://hungarytoday.hu/everyday-life-hungary-coronavirus-measures/ |
| HUN3 | https://konzuliszolgalat.kormany.hu/utazasra-nem-javasolt-tersegek |
|  | **Iceland** |
| ISL1 | https://www.viajesislandia.com/en/coronavirus-in-iceland |
| ISL2 | https://www.government.is/government/covid-19/ |
| ISL3 | https://www.government.is/diplomatic-missions/embassy-article/2020/03/09/response-to-COVID-19-in-Iceland/ |
|  | **India** |
| IND1 | https://www.cnbc.com/2020/03/24/coronavirus-indias-prime-minister-orders-nationwide-lockdown.html |
| IND2 | https://mea.gov.in/press-releases.htm?dtl/32535/Press_Release_by_Ministry_of_Health_and_Family_Welfare__13032020 |
| IND3 | https://www.investindia.gov.in/bip |
|  | **Iran** |
| IRN1 | https://verfassungsblog.de/the-iranian-legal-response-to-covid-19-a-constitutional-analysis-of-coronavirus-lockdown/ |
| IRN2 | https://reliefweb.int/report/iran-islamic-republic/covid-19-response-islamic-republic-iran-april-2020 |
|  | **Ireland** |
| IRL1 | https://www.gov.ie/en/press-release/510383-statement-from-the-national-public-health-emergency-team-on-monday-2/ |
| IRL2 | https://twitter.com/dfatirl |
| IRL3 | https://www.education.ie/en/Press-Events/Press-Releases/2020-press-releases/12-march-2020-statement-from-the-department-of-education-and-skills.html |
| IRL4 | https://www.gov.ie/en/press-release/20fc58-all-pubs-advised-to-close-until-march-29/ |
| IRL5 | https://www.gov.ie/en/news/510383-statement-from-the-national-public-health-emergency-team-on-monday-2/ |
| IRL6 | https://www.irishtimes.com/news/health/coronavirus-government-closes-non-essential-shops-as-seventh-death-confirmed-1.4210785 |
|  | **Israel** |
| ISR1 | https://www.timesofisrael.com/in-unprecedented-move-israelis-advised-to-avoid-all-travel-over-virus-fears/ |
| ISR2 | https://govextra.gov.il/ministry-of-health/corona/corona-virus-en/ |
|  | **Italy** |
| ITA1 | https://www.gazzettaufficiale.it/eli/id/2020/03/01/20A01381/sg |
| ITA2 | https://www.gazzettaufficiale.it/eli/id/2020/03/04/20A01475/sg |
| ITA3 | http://www.governo.it/it/approfondimento/coronavirus-la-normativa/14252 |
|  | **Japan** |
| JPN1* | http://www.asahi.com/ajw/articles/13203373 |
| JPN2 | https://www.japantimes.co.jp/liveblogs/news/coronavirus-outbreak-updates/ |
| JPN3 | http://japan.kantei.go.jp/ongoingtopics/_00013.html |
| JPN4 | https://jw-webmagazine.com/places-in-tokyo-closed-due-to-the-novel-coronavirus-covid-19/ |
|  | **Latvia** |
| LVA1 | https://www.mk.gov.lv/en/aktualitates/stricter-rules-physical-distancing-persons-are-introduced-limit-spread-covid-19 |
| LVA2 | https://eng.lsm.lv/article/society/health/covid-19-emergency-measures-in-english.a351617/ |
| LVA3 | https://likumi.lv/ta/en/en/id/313191-on-declaration-of-the-emergency-situation |
| LVA4 | https://eng.lsm.lv/article/economy/business/sports-centers-shopping-malls-to-close-at-weekends-in-latvia.a353097/ |
| LVA5 | https://www.lrt.lt/en/news-in-english/19/1167272/dispatch-from-riga-latvia-tackles-covid-19-without-hard-lockdown-or-closing-bars |
| LVA6 | https://www.mfa.gov.lv/aktualitates/zinas/65661-aktualizeti-celojuma-bridinajumi-vairakam-valstim |
|  | **Lithuania** |
| LTU1 | https://lietuva.lt/wp-content/uploads/2020/05/Lithuanias-response-to-COVID019-2.pdf |
| LTU2 | https://lietuva.lt/wp-content/uploads/2020/04/UPDATE-April-29.pdf |
| LTU3 | https://www.businessinsider.com/countries-on-lockdown-coronavirus-italy-2020-3#on-march-30-hungarian-prime-minister-viktor-orban-gained-the-power-to-rule-by-decree-indefinitely-and-suspended-elections-7 |
| LTU4* | http://eurireland.ie/2020/04/28/covid-19/ |
| LTU5 | http://keliauk.urm.lt/lt/location/kinija |
| LTU6 | http://www.urm.lt/default/lt/naujienos/uzsienio-reikalu-ministerija-rekomenduoja-atideti-visas-keliones |
| LTU7 | https://nvsc.lrv.lt/lt/naujienos/asmenu-grizusiu-is-uzsienio-prasome-pateikti-informacija |
|  | **Luxembourg** |
| LUX1 | https://www.vdl.lu/en/news/coronavirus-disease-covid-19-preventive-measures |
| LUX2 | https://lu.usembassy.gov/covid-19-information/ |
| LUX3 | https://msan.gouvernement.lu/en/dossiers/2020/corona-virus.html |
| LUX4 | https://gouvernement.lu/fr/actualites/toutes_actualites/communiques/2020/03-mars/16-avis-voyage.html |
| LUX5 | https://gouvernement.lu/en.html |
| LUX6 | https://maee.gouvernement.lu/en/actualites.html |
| LUX7 | https://today.rtl.lu/news/luxembourg/a/1477965.html |
| LUX8 | https://today.rtl.lu/news/luxembourg/a/1480703.html |
| LUX9 | https://today.rtl.lu/news/luxembourg/a/1487151.html |
| LUX10 | https://chronicle.lu/category/at-home/32086-luxembourg-government-announces-futher-restrictions-on-travel-restaurants-non-essential-activities |
| LUX11 | https://luxtimes.lu/luxembourg/40100-germany-border-checks-add-to-luxembourg-virus-lockdown |
| LUX12 | http://legilux.lu/eli/etat/leg/amin/2020/03/16/a149/jo |
| LUX13 | https://fra.europa.eu/sites/default/files/fra_uploads/luxembourg-report-covid-19-april-2020_en.pdf |
| LUX14 | https://maee.gouvernement.lu/fr/actualites.gouvernement%2Bfr%2Bactualites%2Btoutes_actualites%2Bcommuniques%2B2020%2B01-janvier%2B31-avis-de-voyage.html |
|  | **Malta** |
| MLT1* | https://covid19malta.info |
| MLT2* | http://www.justiceservices.gov.mt/DownloadDocument.aspx?app=lp&itemid=30021&l=1 |
| MLT3* | http://www.justiceservices.gov.mt/DownloadDocument.aspx?app=lp&itemid=30004&l=1 |
| MLT4* | http://www.justiceservices.gov.mt/DownloadDocument.aspx?app=lp&itemid=30003&l=1 |
| MLT5* | http://www.justiceservices.gov.mt/DownloadDocument.aspx?app=lp&itemid=30024&l=1 |
| MLT6* | http://www.justiceservices.gov.mt/DownloadDocument.aspx?app=lp&itemid=30030&l=1 |
|  | **Netherlands** |
| NLD1 | https://www.government.nl/topics/coronavirus-covid-19/tackling-new-coronavirus-in-the-netherlands |
| NLD2 | https://www.netherlandsandyou.nl/latest-news/news/2020/03/17/covid-update-17-march |
| NLD3 | https://dutchreview.com/news/coronavirus-netherlands/ |
| NLD4 | https://www.iscresearch.com/cornavirus-covid-19-update |
| NLD5 | https://www.government.nl/latest/news/2020/03/12/new-measures-to-stop-spread-of-coronavirus-in-the-netherlands |
| NLD6 | https://www.government.nl/topics/coronavirus-covid-19/tackling-new-coronavirus-in-the-netherlands/faqs-about-approach-to-tackling-coronavirus |
|  | **New Zealand** |
| NZL1 | https://www.businessinsider.com/washington-post-rave-review-new-zealand-coronavirus-response-2020-4 |
| NZL2 | https://safetravel.govt.nz/news-features |
| NZL3 | https://www.beehive.govt.nz/release/new-zealand-restrict-travel-china-protect-against-coronavirus |
| NZL4 | https://www.forbes.com/sites/kaeliconforti/2020/03/23/alert-level-4-restrictions-to-begin-in-new-zealand-this-week/#21eae0c966a0 |
|  | **Norway** |
| NOR1 | https://www.regjeringen.no/en/topics/foreign-affairs/reiseinformasjon/travel_coronavirus/id2691821/ |
| NOR2 | https://www.regjeringen.no/no/tema/utenrikssaker/reiseinformasjon/velg-land/reiseinfo_kina/id2414833/ |
|  | **Poland** |
| POL1 | https://gis.gov.pl/wypoczynek/koronawirus-wypoczynek/informacje-i-zalecenia-pl-koronawirus-wypoczynek/komunikaty-dla-podrozujacych-archiwum/ |
| POL2 | http://prawo.sejm.gov.pl/isap.nsf/download.xsp/WDU20020620558/U/D20020558Lj.pdf |
| POL3 | http://prawo.sejm.gov.pl/isap.nsf/download.xsp/WDU20200000492/O/D20200492.pdf |
|  | **Portugal** |
| PRT1 | https://expresso.pt/coronavirus/2020-03-13-Covid-19.-Estado-de-alerta-ate-pelo-menos-9-de-abril |
| PRT2 | http://www.presidencia.pt/?idc=22&idi=176060 |
| PRT3 | https://www.theportugalnews.com/news/covid-19-portugal-update/53286 |
| PRT4 | https://www.garda.com/crisis24/news-alerts/324811/portugal-government-implements-state-of-emergency-measures-due-to-covid-19-march-19-update-5 |
| PRT5 | https://www.lusa.pt/article/Hg9xOvEPmRViWg_AilznaTMSZM5iuSI1/portugal-government-approves-30-measures-to-help-prevent-spread-of-covid-19 |
| PRT6 | https://www.portugal.gov.pt/pt/gc22/comunicacao/noticia?i=o-primeiro-dever-e-protegermo-nos-uns-aos-outros-do-contagio |
|  | **Romania** |
| ROM1 | https://www.romania-insider.com/coronavirus-romania-football-suspended |
| ROM2 | https://www.mai.gov.ro/wp-content/uploads/2020/03/ordinance-3-EN-1.pdf |
| ROM3 | http://www.mae.ro/travel-alerts/51435 |
| ROM4 | http://www.mae.ro/node/51759 |
|  | **Russia** |
| RUS1 | https://www.aljazeera.com/news/2020/03/coronavirus-sporting-events-affected-outbreak-200310084205890.html |
| RUS2 | https://tass.com/world/1116027 |
| RUS3 | https://home.kpmg/xx/en/home/insights/2020/04/flash-alert-2020-154.html |
|  | **Singapore** |
| SGP1 | https://www.gov.sg/features/covid-19 |
| SGP2 | https://www.tech.gov.sg/products-and-services/responding-to-covid-19-with-tech/ |
| SGP3 | https://www.moh.gov.sg/covid-19/past-updates |
|  | **Slovakia** |
| SVK1 | https://www.mzv.sk/web/en/news?rok=2020&mesiac=2 |
| SVK2 | https://www.svf.stuba.sk/sk/dianie-na-svf/opatrenia-na-zamedzenie-sirenia-ochorenia-covid-19-v-sr.html?page_id=7961EKp_redirect=%2Fweb%2Fen%2Fnews%3Frok%3D2020%26mesiac%3D2 |
| SVK3 | https://www.mzv.sk/web/en/news/current_issues/-/asset_publisher/lrJ2tDuQdEKp/content/krizovy-stab-aktualne-opatrenia-v-suvislosti-s-koronavirusom/10182?_101_INSTANCE_lrJ2tDuQdEKp_redirect=%2Fweb%2Fen%2Fnews%3Frok%3D2020%26mesiac%3D1 |
|  | **Slovenia** |
| SVL1 | https://en.wikipedia.org/wiki/2020_coronavirus_pandemic_in_Slovenia |
| SVL2 | https://www.gov.si/en/news?org%5B0%5D=34&start=20 |
|  | **South Africa** |
| ZAF1 | https://www.gov.za/speeches/statement-president-cyril-ramaphosa-measures-combat-covid-19-epidemic-15-mar-2020-0000 |
| ZAF2 | https://www.gov.za/speeches/statement-minister-basic-education-mrs-angie-motshekga-inter-ministerial-media-briefing-0 |
| ZAF3 | https://www.sciencemag.org/news/2020/04/south-africa-flattens-its-coronavirus-curve-and-considers-how-ease-restrictions |
| ZAF4 | https://en.wikipedia.org/wiki/COVID-19_pandemic_in_South_Africa#Isolation_and_lockdown |
| ZAF5 | https://theintercept.com/2020/03/15/african-nations-turn-the-tables-imposing-travel-restrictions-against-u-s-europe-and-china-to-stave-off-coronavirus/ |
|  | **South Korea** |
| KOR1 | https://www.japantimes.co.jp/news/2020/03/01/asia-pacific/science-health-asia-pacific/south-korea-coronavirus-4/#.Xq88movVLwc |
| KOR2 | https://www.straitstimes.com/sport/coronavirus-south-korea-postpones-k-league-football-season-table-tennis-world-cships-in-busan |
| KOR3 | https://english.visitkorea.or.kr/enu/AKR/FU_EN_15.jsp?cid=2648107 |
| KOR4 | http://english.hani.co.kr/arti/english_edition/e_national/933281.html |
|  | **Spain** |
| ESP1 | https://www.mscbs.gob.es/profesionales/saludPublica/ccayes/alertasActual/nCov-China/documentos/Actualizacion_80_COVID-19.pdf |
| ESP2 | https://gogoespana.com/en/blog/coronavirus-situation-in-spain/ |
| ESP3 | https://twitter.com/SpainMFA |
|  | **Sweden** |
| SWE1 | https://se.usembassy.gov/covid-19-coronavirus-information/ |
| SWE2 | https://www.government.se/government-of-sweden/ministry-for-foreign-affairs/ |
|  | **Switzerland** |
| CHE1 | https://www.ncregister.com/blog/solenetadie/the-church-in-switzerland-adopts-strict-measures-to-address-coronavirus |
| CHE2 | https://www.swissinfo.ch/eng/coronavirus-crisis-_has-switzerland-got-enough-hospital-beds--/45671704 |
| CHE3 | https://www.swissinfo.ch/eng/coronavirus_intensive-care-beds-could-run-out-on-thursday--study-predicts/45647658 |
| CHE4 | http://www.corona-data.ch |
| CHE5 | https://www.eda.admin.ch/beijing |
|  | **Taiwan** |
| TWN1 | https://www.thestar.com.my/news/regional/2020/02/27/big-gatherings-canceled-across-taiwan-due-to-coronavirus-scare |
| TWN2 | https://www.cdc.gov.tw/En |
| TWN3 | https://www.boca.gov.tw/cp-220-5081-c06dc-2.html |
| TWN4 | https://www.taiwan.gov.tw/news.php |
|  | **Turkey** |
| TUR1* | http://eurireland.ie/2020/04/28/covid-19/ |
| TUR2 | https://www.aa.com.tr/en/health/turkey-issues-coronavirus-travel-advisory-for-public/1762540 |
| TUR3 | https://www.garda.com/crisis24/news-alerts/319626/turkey-restrictions-implemented-for-travelers-from-china-south-korea-iran-iraq-and-italy-march-3-update-3 |
|  | **United Kingdom** |
| GBR1 | https://www.gov.uk/government/news/travel-advice-foreign-secreatary-statement-17-march-2020 |
| GBR2 | https://www.gov.uk/government/topical-events/coronavirus-covid-19-uk-government-response%20 |
| GBR3 | https://www.gov.uk/coronavirus |
|  | **United States of America** |
| USA1 | https://www.usa.gov/coronavirus |
| USA2 | https://www.state.gov/coronavirus/ |
| USA3 | https://ar.usembassy.gov/u-s-government-action-plan-to-support-the-international-response-to-covid-19/ |
| USA4 | https://www.coronavirus.gov |
|  | **Global Resources** |
| GLR1 | https://en.wikipedia.org/wiki/Travel_restrictions_related_to_the_2019–20_coronavirus_pandemic |
| GLR2 | https://www.nytimes.com/article/coronavirus-travel-restrictions.html |
| GLR3 | https://www.dw.com/en/coronavirus-what-are-the-lockdown-measures-across-europe/a-52905137 |
| GLR4* | https://www.iscresearch.com/cornavirus-covid-19-update |
| GLR5 | https://www.iatatravelcentre.com/international-travel-document-news/1580226297.htm |
| GLR6 | https://www.bbc.com/sport/51605235 |
| GLR7 | https://en.wikipedia.org/wiki/2019–20_coronavirus_pandemic |
| GLR8 | https://fra.europa.eu/en/publication/2020/covid19-rights-impact-april-1#TabPubStudies |

* The content at the specific links is no longer available
