# Supplementary figures and images for: COVID-19 pandemic spread against countries’ non-pharmaceutical interventions responses: a data-mining driven comparative study
Source: BMC Public Health. 2021 Sep 1;21:1607. doi: 10.1186/s12889-021-11251-4 (PMC8409702; doi:10.1186/s12889-021-11251-4)

# **Additional File #3: Appendix - COVID-19 Time Series Analysis Plots**


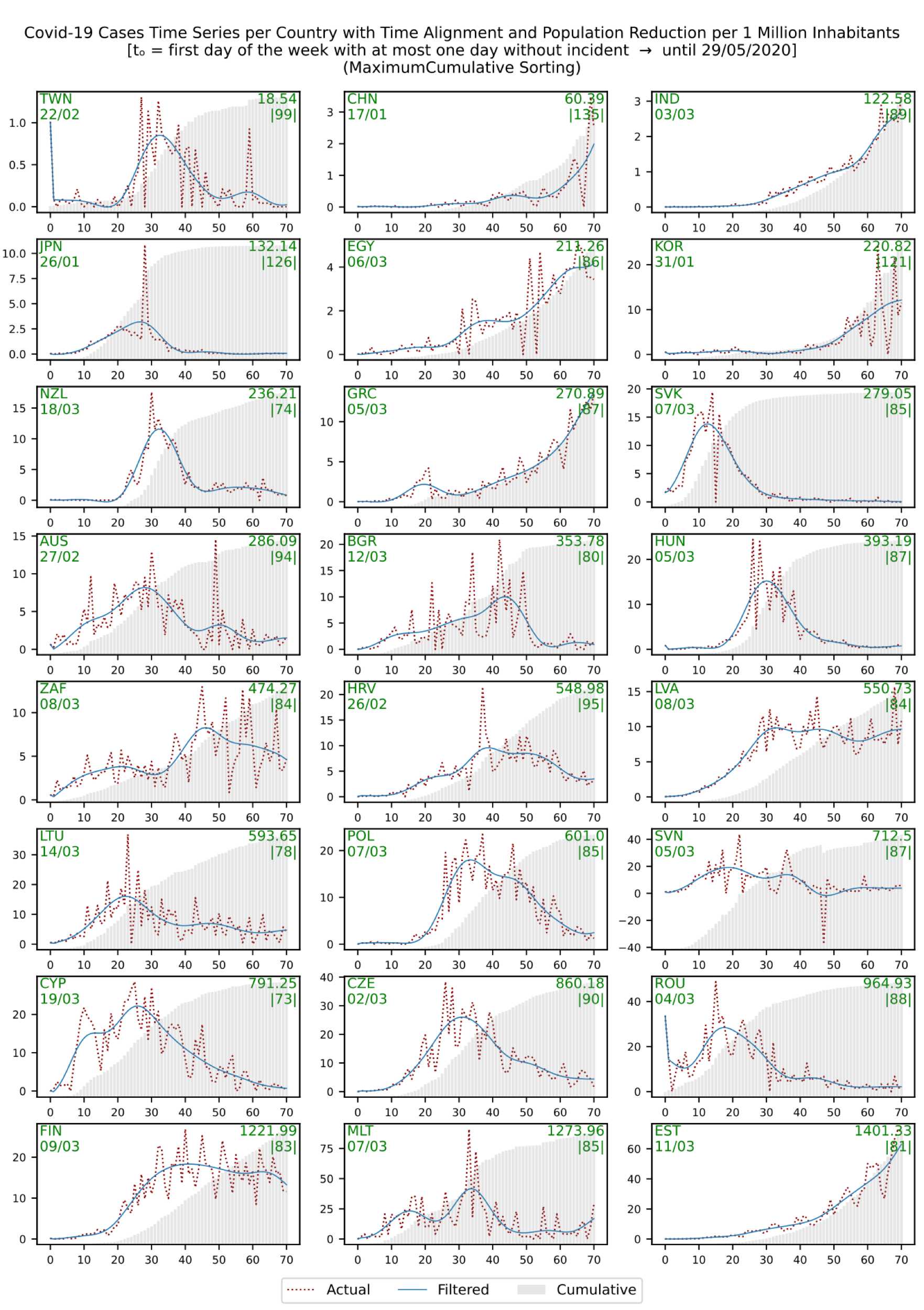

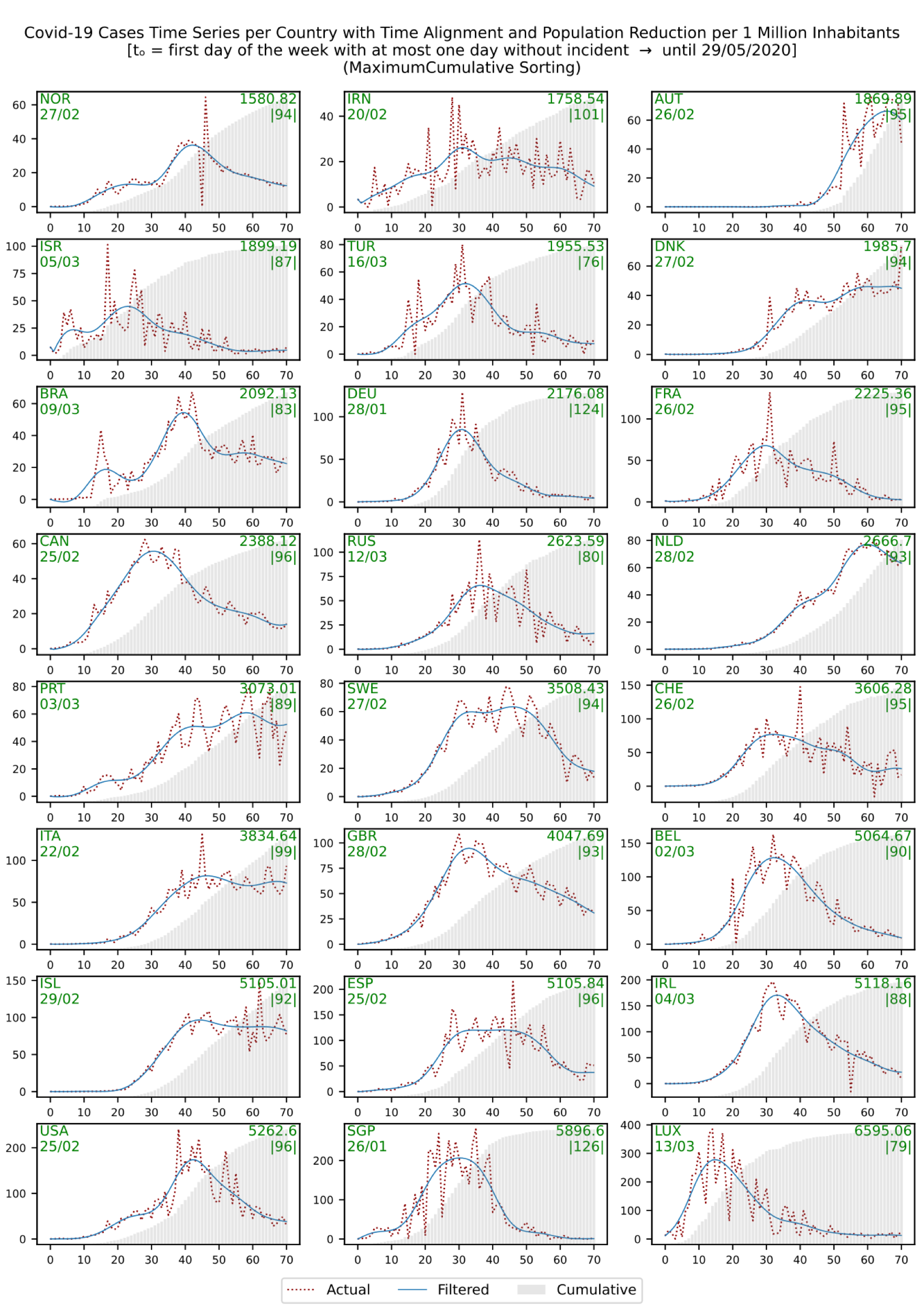

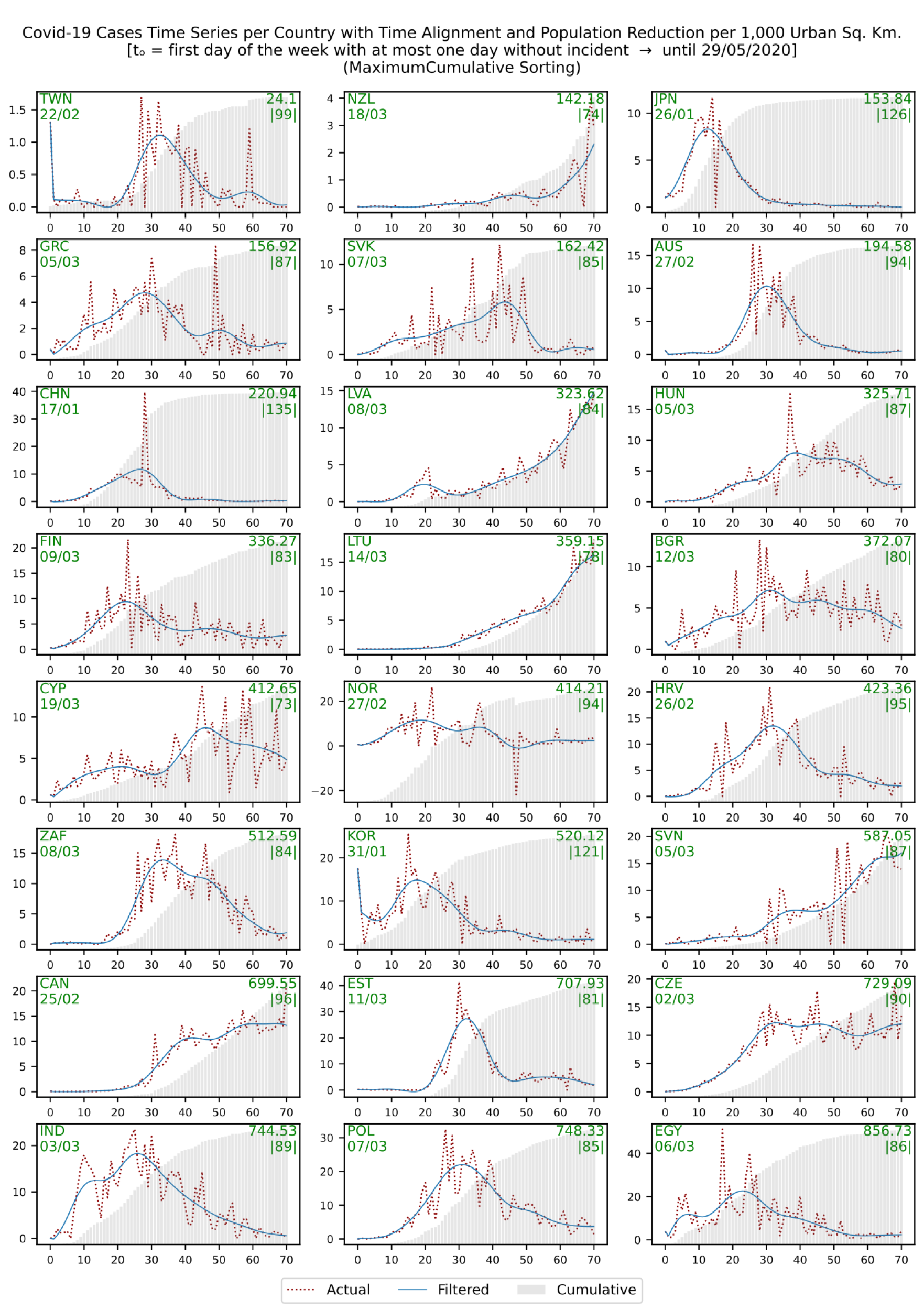

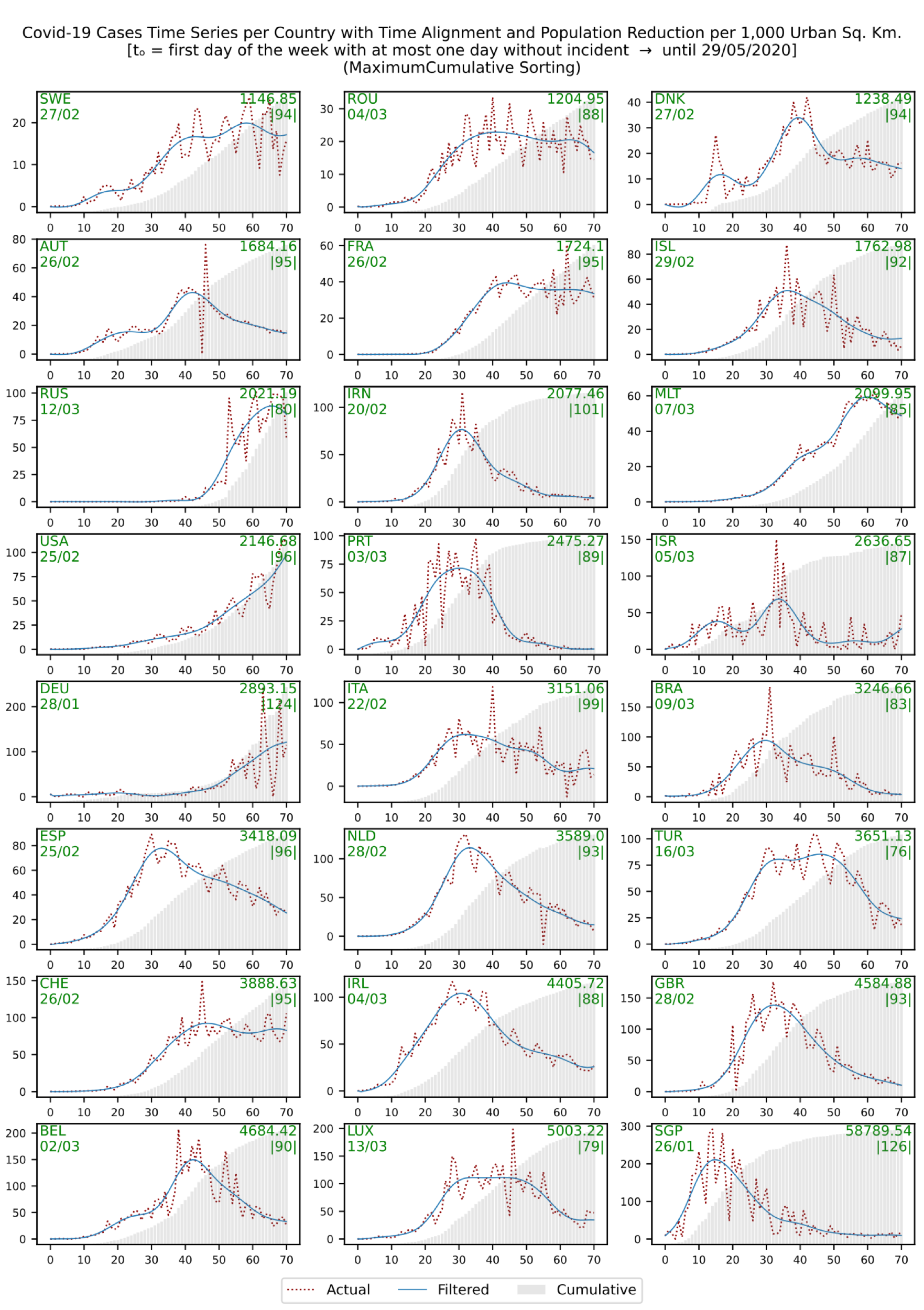

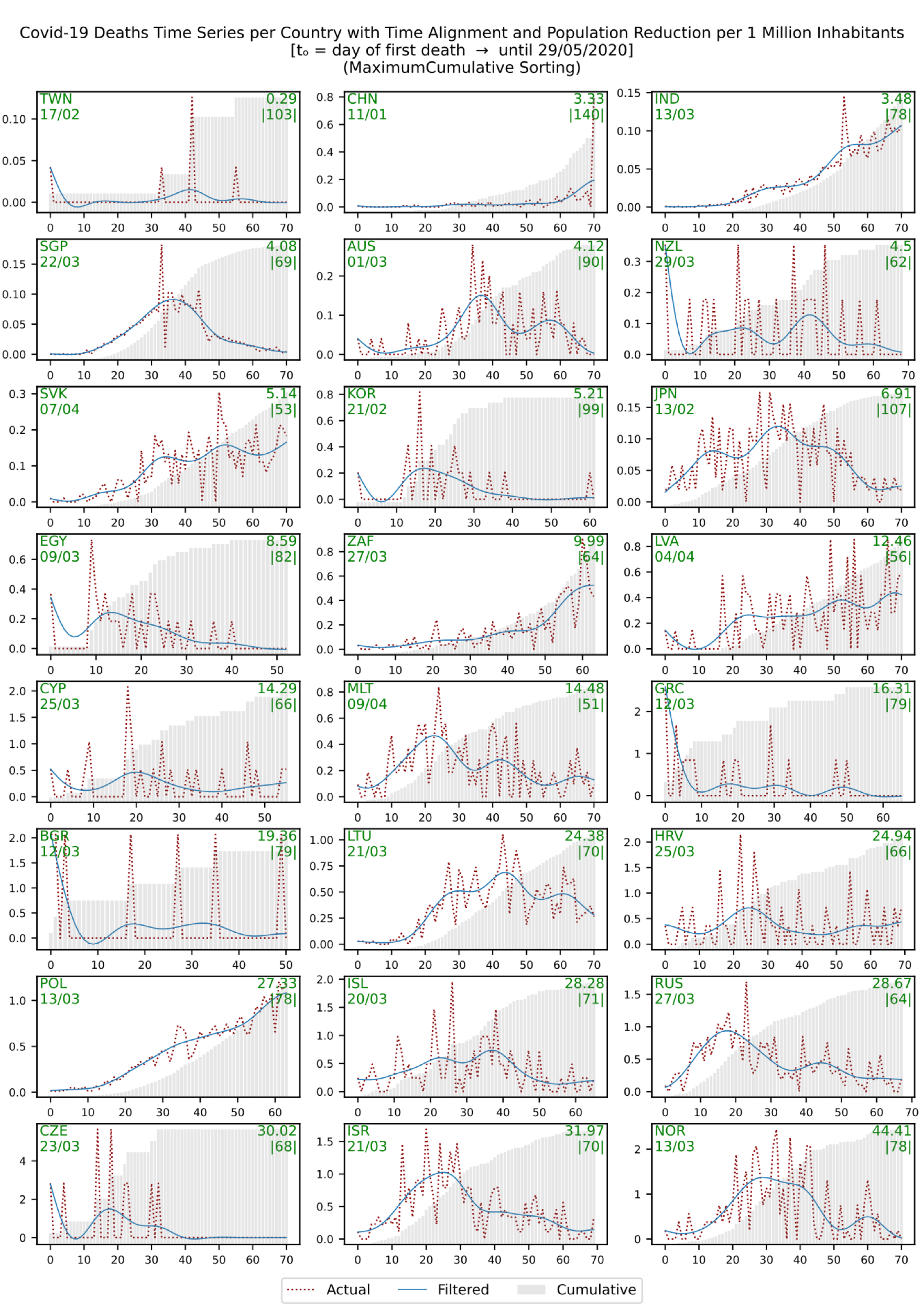

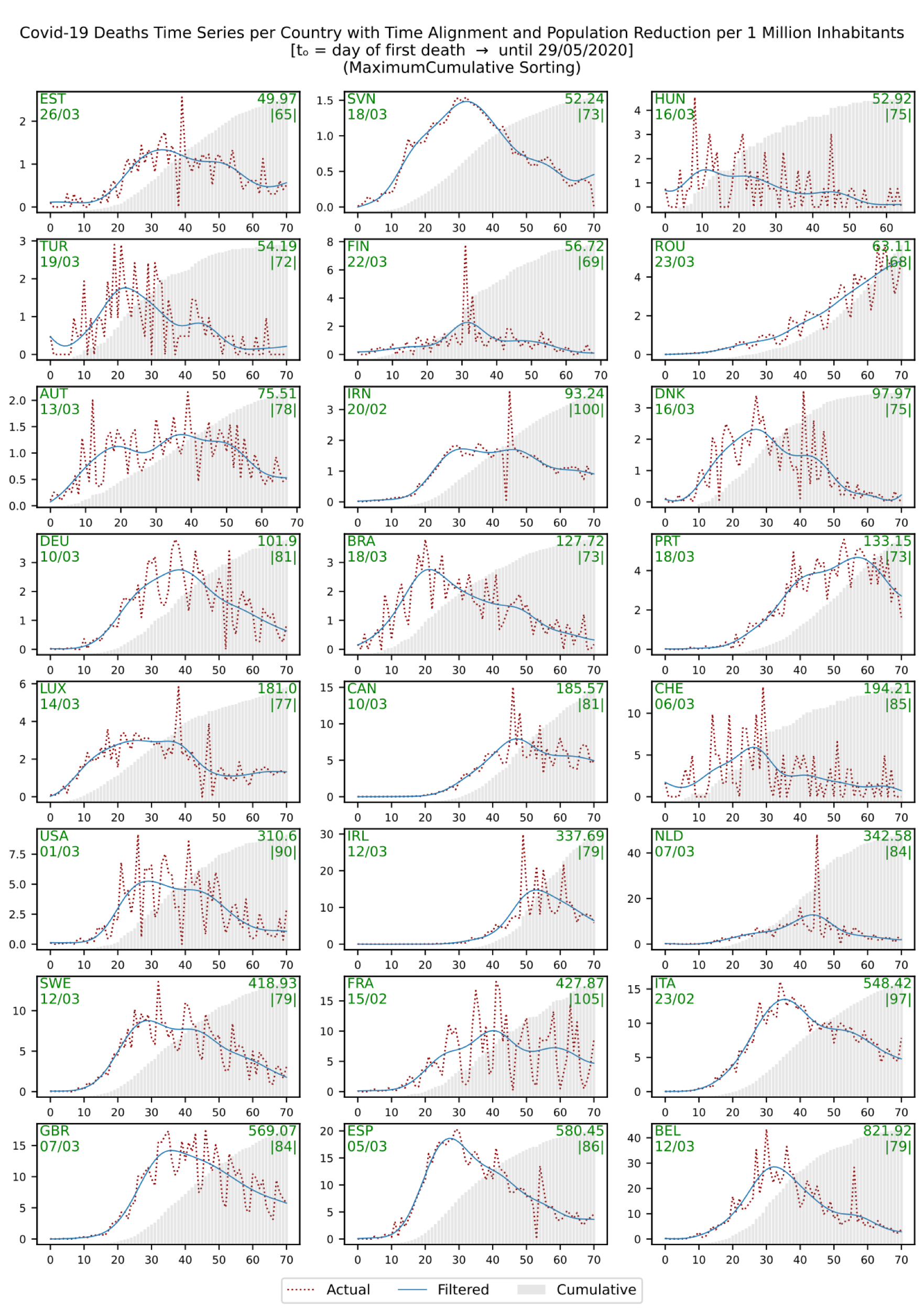

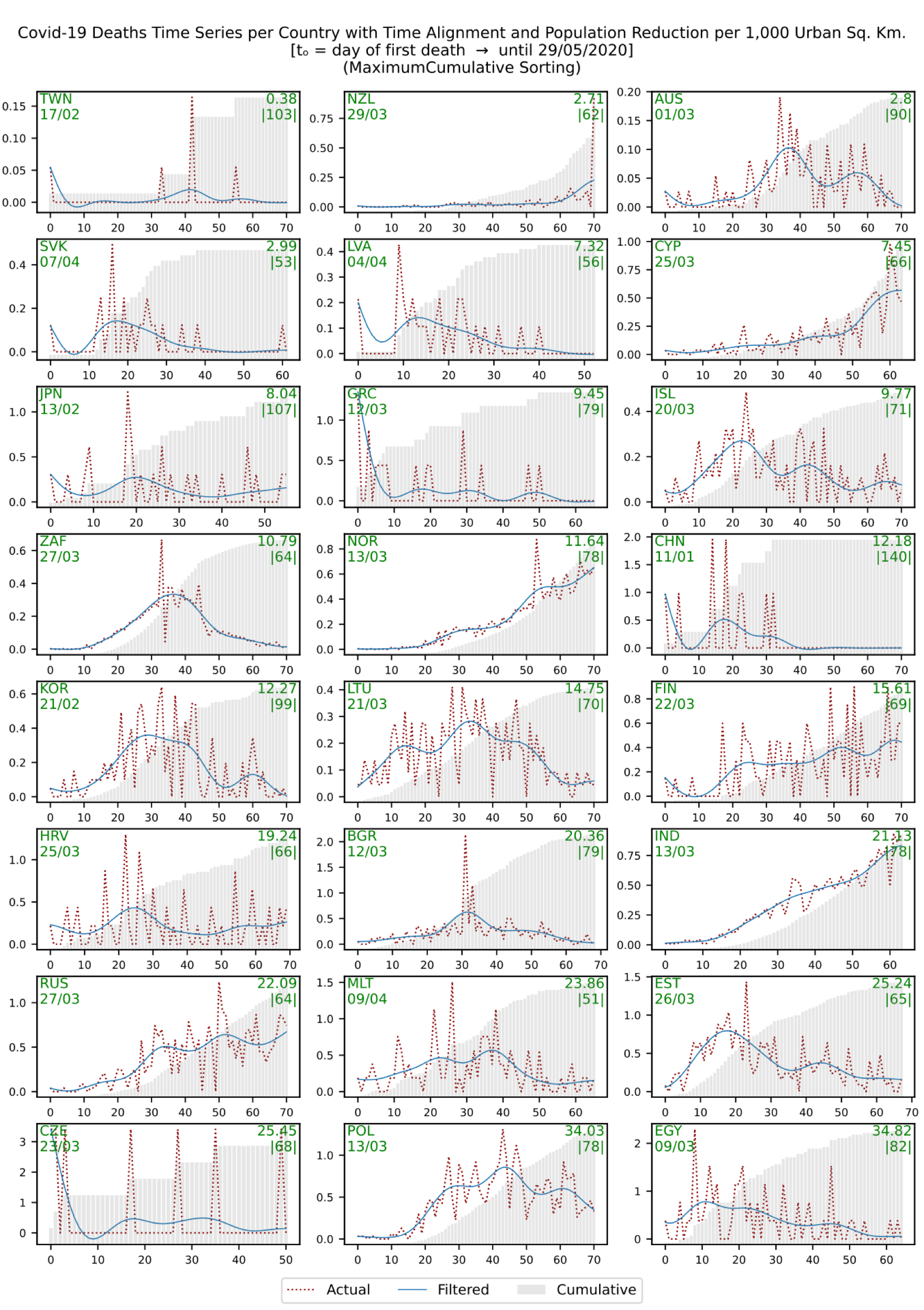

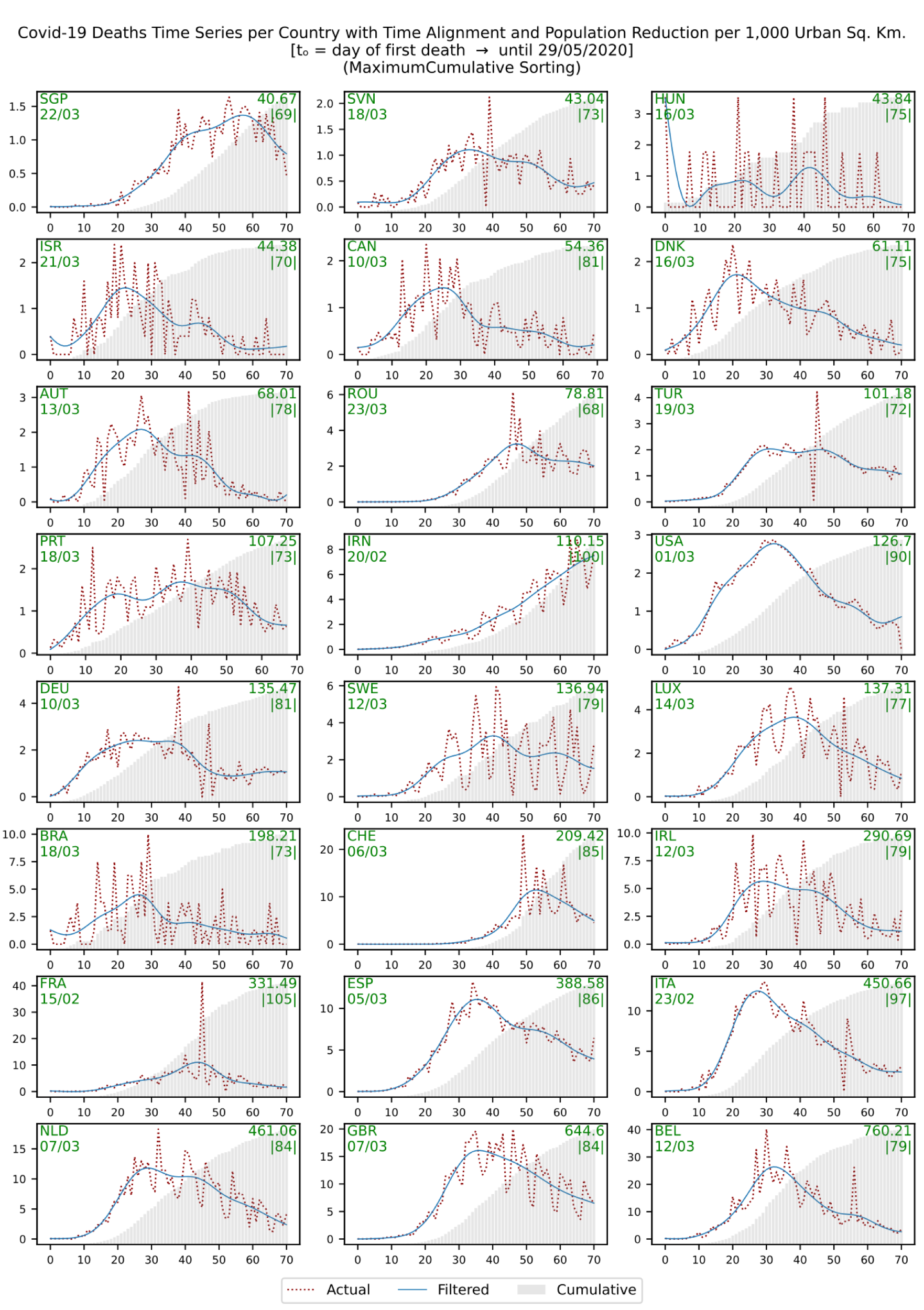

Supplement: Supplementary file 3 — Additional file 3: Appendix - COVID-19 Time Series Analysis Plots [file 12889_2021_11251_MOESM3_ESM.docx]
